# Thin Conducting Films: Preparation Methods, Optical and Electrical Properties, and Emerging Trends, Challenges, and Opportunities

**DOI:** 10.3390/ma17184559

**Published:** 2024-09-17

**Authors:** Razia Khan Sharme, Manuel Quijada, Mauricio Terrones, Mukti M. Rana

**Affiliations:** 1 Division of Physics, Engineering, Mathematics and Computer Sciences, and Research on Nanomaterial-Based Integrated Circuits and Electronics (NICE), Delaware State University, Dover, DE 19901, USA; khansharme@gmail.com; 2 NASA Goddard Space Flight Center, 8800 Greenbelt Road, Greenbelt, MD 20771, USA; manuel.a.quijada@nasa.gov; 3 Department of Physics, The Pennsylvania State University, 104 Davey Lab, PMB 196, University Park, PA 16802, USA; mut11@psu.edu

**Keywords:** thin conducting film (TCF), preparation/methodology, optical properties, electrical properties, future trends in TCFs, thin film

## Abstract

Thin conducting films are distinct from bulk materials and have become prevalent over the past decades as they possess unique physical, electrical, optical, and mechanical characteristics. Comprehending these essential properties for developing novel materials with tailored features for various applications is very important. Research on these conductive thin films provides us insights into the fundamental principles, behavior at different dimensions, interface phenomena, etc. This study comprehensively analyzes the intricacies of numerous commonly used thin conducting films, covering from the fundamentals to their advanced preparation methods. Moreover, the article discusses the impact of different parameters on those thin conducting films’ electronic and optical properties. Finally, the recent future trends along with challenges are also highlighted to address the direction the field is heading towards. It is imperative to review the study to gain insight into the future development and advancing materials science, thus extending innovation and addressing vital challenges in diverse technological domains.

## 1. Introduction

Thin conducting films play a crucial role in various technological applications, spanning industries such as optoelectronics, materials science, and more [1,2]. Essentially, they have a high surface-to-volume ratio and nanoscale thickness, which enable them to display increased features like mechanical flexibility, optical transparency, and electrical conductivity. These unique characteristics impart distinct and often enhanced properties to the materials, setting them apart from their bulk counterparts. Due to their unique qualities, including tailored properties [3], surface modification [4], miniaturization [5], improved efficiency [6], versatility [7], cost-effectiveness [8], research and development, and adaptability [9], they are highly advantageous, making them essential to contemporary industry and technology. Therefore, thin conducting films (TCFs) are widely used in various sectors and technological fields. They are employed in electronics and semiconductor production as functional layers in sensors, thin-film transistors, and integrated circuits, making electronic devices smaller and helping them to perform better [10,11]. To control light transmission, absorption, and reflection, there are several thin-film coatings in optical and electronic applications, such as photovoltaic cells, anti-reflective coatings, optical filters, etc. [12,13]. TCFs are used in the automobile sector to improve the endurance and visual appeal of, for example, anti-scratch, corrosion-resistant, and self-cleaning coatings [14]. They are also essential to energy-related technologies, including fuel cells, batteries, and solar cells, which function as active layers for electrochemical reactions, light absorption, and charge storage [15]. Furthermore, TCFs provide biocompatible and functional surfaces for treatments and diagnostics in biomedical applications such as drug delivery systems, biosensors, and medical implants [16,17]. Moreover, thin films have uses in a wide range of industries, including packaging [13], aerospace, NASA [18], telecommunications [19], and many other areas, demonstrating their versatility and influence in a variety of industries. As TCFs have widespread use in various technological applications, we need to understand more about their preparation as well as their optical and electrical properties. Precise control over the film composition, thickness, structure, and crystallinity can be developed with a profound grasp of TCFs’ preparation techniques, providing customized properties for targeted applications. Designing devices with the appropriate light absorption, transmission, and reflection properties also requires understanding the optical properties of thin films. However, optimizing the device performance for electrical and optoelectronic applications requires a thorough grasp of TCFs’ properties, such as resistivity, bandgap, and conductivity [20]. It is crucial to keep expanding our understanding of thin-film science and technology since it has the potential to spur innovation and transform several sectors.

A significant study has already been completed in the broad area of thin-film review. Reviews have been conducted on thin films for photovoltaics [21], organic thin-film transistors (OTFTs) [22,23], thin-film composite membranes [24], thin-film growth [25,26], TiNi-based thin films [27], thin-film solar cell technologies and their limitations [28,29], conductive metal nanomaterials [30], thin-film transistors from oxide semiconductors [31], flexible oxide semiconductor thin-film transistors [32], piezoelectric thin films [33,34], a-Si-based thin-films [35], printed oxide thin-film transistors [36], thin-films for sensing terahertz waves [37], and ferroelectric thin films [38], among many others. Additionally, Nagdhi et al. [30] reviewed various conductive metal nanomaterials and examined the difficulties associated with thin-film deposition techniques and their applications as conductive coatings. Adedokun [39] reviewed transparent conductive oxide thin films, where he highlighted the effectiveness of aluminum-doped zinc oxide (AZO), aluminum-doped tin oxide (ATO), and fluorine-doped tin oxide (FTO) as long-term substitutes for indium-doped tin oxide (ITO). A similar review was conducted by Afre et al. [40], where they investigated transparent conductive oxide films for numerous applications. Elanjeitsenni et al. [41] reported a review of thin-film technology to create conducting polymer-based sensors that can be used in various applications. Each of the above-mentioned studies has provided a concise overview of the specific area that they have investigated in their respective reviews. This suggests that this field of study is acknowledged for its significance and influence in the broader context of thin-film research. It also demonstrates its applicability to scientists, engineers, and industry professionals in various fields, including electronics, photonics, coatings, and renewable energy. This makes it possible to use this as a benchmark or reference point for future studies. Owing to the extraordinary diversity of thin conducting films and their wide range of uses, it is not easy to delve into further detail about every area. Yet, it is possible to emphasize the most significant and widely utilized thin films (conducting) and address each area’s specific aspects. However, as discussed, reviewed research studies are lacking that focus on providing a brief general overview of TCFs without concentrating on any particular area. This absence leaves a substantial gap in the literature as there is a need for reviews that provide insights into a range of important thin-film areas. These reviews would make it simple for researchers to obtain data from a single study on various thin-film materials and applications, TCFs in particular.

Herein, this review offers insights into some of the most common and significant TCFs used. The most popular and commonly used methods to deposit TCFs are explained, focusing on the fundamentals, advantages, and drawbacks associated with each of them. It has offered insightful analysis, synthesized the prior research, and suggested new approaches to address the challenges in the field. This study has also demonstrated the characteristics of various TCFs in terms of both their optical and electrical properties. We have also highlighted the factors that influence these properties and how they can impact the behavior of the films. By examining these parameters, we gain a better understanding of how to optimize the performance of the TCFs for specific applications. The possible future trends of these thin films in the area of two-dimensional (2D) materials are also discussed briefly. This work establishes a baseline for ongoing thin-film science and technology research and development endeavors, offering a framework on which these investigations might grow and expand.

## 2. Preparation/Methodology

Deposition for TCFs can be achieved in various ways. Further post-treatment or annealing procedures could improve the structural integrity and conductivity even more [42]. This choice in process depends on the available resources, cost, accuracy, and specific applications. The article discusses some of the deposition techniques, including the most and least commonly used methodologies. Different materials are used in this coating process depending on the technique to be followed. Depending on the coating, the thin films show different characteristics. Sometimes, foreign elements are introduced to alter or improve the thin film’s performance. The techniques for deposition can be broadly divided into vacuum-based and solution-processed (non-vacuum-based). A regulated vacuum environment is necessary to produce uniformly high-quality thin films for vacuum-based techniques. Non-vacuum-based techniques, such as solution-processed methods, operate at atmospheric pressure and have simpler setups than the vacuum processes, although their film quality attributes are usually different. Table 1 below shows the three types of methods: vacuum-based, solution process, and mixed category.

### 2.1. Vaccum-Based Depositions

#### 2.1.1. Physical Vapor Deposition (PVD)

The PVD process is a widespread and well-known coating technique. Having low maintenance and cost-effectiveness, it is well-established [43]. PVD deposition can help to achieve durability and toughness in machining, increasing hardness in extreme environments, chemical reaction stability, abrasion resistance, and stiffness [44]. It has a great deal of applications, such as enhancement of resistance against wear and corrosion, functional applications, such as tools and decorative pieces, and optical enhancement [45]. It has the unique feature of creating homogeneous layers, which is difficult to achieve by other processes [45]. Due to its friendly nature and characteristics, it is widely used for TCF deposition. As a result, scientists are working on enhancing and improving the techniques.

##### Evaporation

Among the techniques used in PVD processes, evaporation is the primary method of depositing TCFs. In this technique, the source material, which is in a condensed phase, is heated to its vapor phase. Most of these source materials are used in liquid rather than solid states [46]. These vaporized atoms form a thin film into a substrate in a vacuum environment. The substrate used in this process may maintain a required temperature depending on the properties needed. This technique’s vacuum environment helps to reduce impurities, maintain homogeneity, and lower evaporation temperature [47]. To ensure a straight line while evaporating from the 10–50 cm distance of the source to the substrate, a pressure of 0.013332 Pascals is usually maintained [48]. The maximum number of evaporated vaporized atoms can be approximated using Equation (1) [49],

(1) $$\frac{dN_{e}}{A_{e}dt}=\frac{P_{e}}{\sqrt{2\pi KT}}$$

Here, the rate of change in the number of evaporated atoms ($N_{e}$) per unit area (*A*) per unit time (*t*) is represented by $\frac{dN_{e}}{A_{e}dt}$. It explains the rate at which atoms or molecules are leaving a surface or a volume. $P_{e}$ is the equilibrium pressure of the gas, *K* is the Boltzmann constant, and *T* is the system’s absolute temperature. If the saturated vapor pressure is represented by $P_{v}$ and the bounding surface is composed of liquid or solid condensate in equilibrium with its saturated vapor, then the rate at which the vapor molecules strike the surface may be determined using Equation (1) with $P_{e}$ = $P_{v}$. From the Langmuir expression, the rate of free evaporation of the vaporized atoms in a vacuum can be calculated using Equation (2) [50],

(2) $$m_{e}=5.83\times 10^{-2}\;P_{e\;}{\left(\frac{M}{T}\right)}^{\frac{1}{2}}\;g\;{\mathrm{cm}}^{-2}\;s^{-1}$$

Here, $m_{e}$, *T*, *M*, and $P_{e\;}$ represent each evaporating species’ evaporation rate, temperature, molar mass, and equilibrium pressure (0.013332 Pascals), respectively. This formula is a particular use of the Langmuir expression that has been modified for practical use. In the case of molecules, the evaporation rate can be written as the following Equation (3) [51]. This version of the equation has been tailored for computations involving moles of evaporated species per unit area per second. Here, $N_{e}$, $P_{e\;}$, M, and T represent the evaporation rate in moles per unit area per second, equilibrium vapor pressure of the substance, molar mass of the evaporating species, and absolute temperature,

(3) $$N_{e}=3.513\times 10^{22}\;P_{e\;}{\left(\frac{1}{MT}\right)}^{\frac{1}{2}}\;\mathrm{mol}\;{\mathrm{cm}}^{-2}\;s^{-1}$$

**Figure 1:**
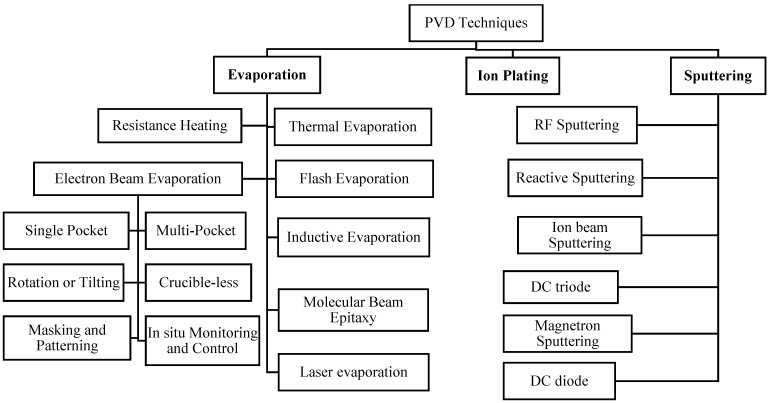
Different types of PVD techniques for depositing TCFs.

However, different evaporation techniques are available for thin-film deposition. Some of the important processes are shown in Figure 1. These techniques are straightforward and convenient. Moreover, the rate of vaporized deposition of the atom on the substrate in each technique varies with the geometry of the source, distance, positioning of the substrate to the source, and condensation coefficient [52].

##### Thermal Evaporation

**Figure 2:**
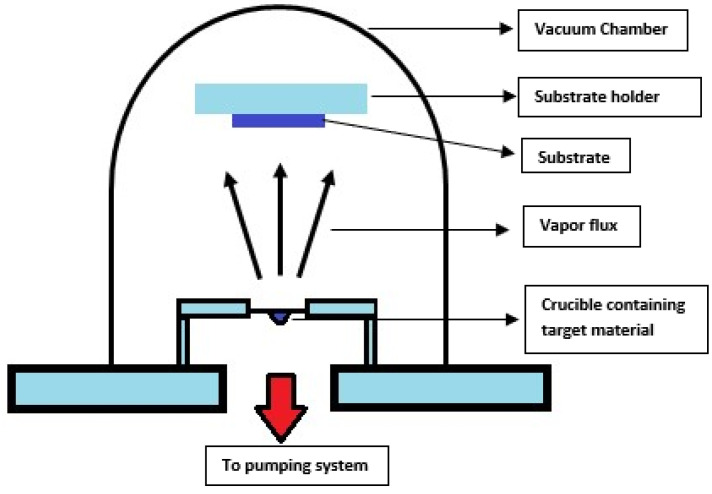
The thermal evaporation phenomenon.

Among different evaporation techniques, thermal evaporation is the simplest available form. In this process, a resistive heating source is employed to heat the source material, causing the evaporated material to condense onto the substrate by generating vapor flux and forming a thin film, as illustrated in Figure 2. A vacuum chamber is utilized for this procedure, within which a substrate holder is responsible for holding the substrate in place. For thin-film transistors, solar cells, and organic light-emitting diodes (OLEDs), thermal evaporation deposits metals such as gold (Au), silver (Ag), copper (Cu), and aluminum (Al) [53]. In insulation layers and capacitors, various dielectric materials are used as thin films using thermal evaporation, and those are composed of oxides and nitrides. For example, TCFs, such as CuI films [54], are prepared using thermal evaporation and reported by different authors, Shi et al. [55], Moditswe et al. [54], and Kaushik et al. [56]. ITO thin films are also deposited using this technique.

##### Electron Beam Evaporation (EBE)

**Figure 3:**
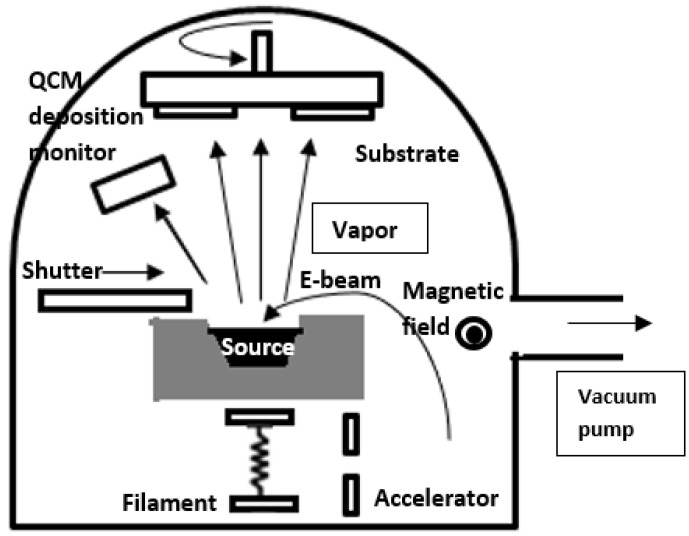
EBE technique.

EBE is used to ensure good quality and purity in thin films. In the EBE, the source material is evaporated by a highly charged electron beam performed under a vacuum environment. The process enables directional deposition, which helps to obtain precise texture and film characteristics [57]. The process produces a very high-purity thin film for this accurate control as it has little contamination. Materials with high melting points are ideal for this technique [58]. For most of the fabrication of thin films, this process is commonly used in the application of electronic, optical, and other research areas. The schematic diagram for this process is shown in Figure 3. Electrons are accelerated toward the source material by an accelerator when emitted from a filament in EBE. The source material evaporates into vapor when heated by the electron beam (E-beam). Next, the vapor moves in the direction of the substrate, where it condenses to produce a thin layer. A QCM (quartz crystal microbalance) monitor keeps track of film thickness, while a shutter manages the deposition procedure. A vacuum pump facilitates the system’s operation under vacuum, guaranteeing ideal deposition conditions. A diversity of TCFs is prepared using this synthesis process; for example, zinc oxide (ZnO) as transparent coating [59], ITO as transparent conductive material [60], Au in decorative coatings [61], doped TiO_2_ as optical coatings [62], and others. Some of the most common classifications of EBE techniques are shown in Figure 1. In EBE, a single pocket represents a particular place or crucible containing the source material to evaporate, often loaded into a crucible. The multi-pocket crucible is comparable to a single pocket but includes several crucibles or pockets holding a different substance for a particular case. Therefore, this makes it possible to deposit composite or multilayered films without disrupting the vacuum. In some cases, the EBE technique enables the rotation of the substrate during the time of deposition and a more accurate and uniform result is achieved; this process is called rotation or tilting EBE [63]. However, when the source material used in this process is in the form of a rod or wire, it does not generally need a crucible to evaporate, and the electron beam impinges directly so that the thin film can be deposited. This process is less expensive as it does not require any crucible. Moreover, the masking and patterning EBE process refers to creating specific feature patterns on a substrate. With the growing technology and advancements in monitoring tools and devices, in situ control has become increasingly sophisticated and precise. We can now use real-time monitoring tools for deposition. The in situ monitoring and control EBE technique is used in spectroscopic analysis, where we measure the thickness of thin film [64]. Additionally, we can achieve the desired thin-film properties by making the required modifications.

##### Molecular Beam Epitaxy (MBE)

**Figure 4:**
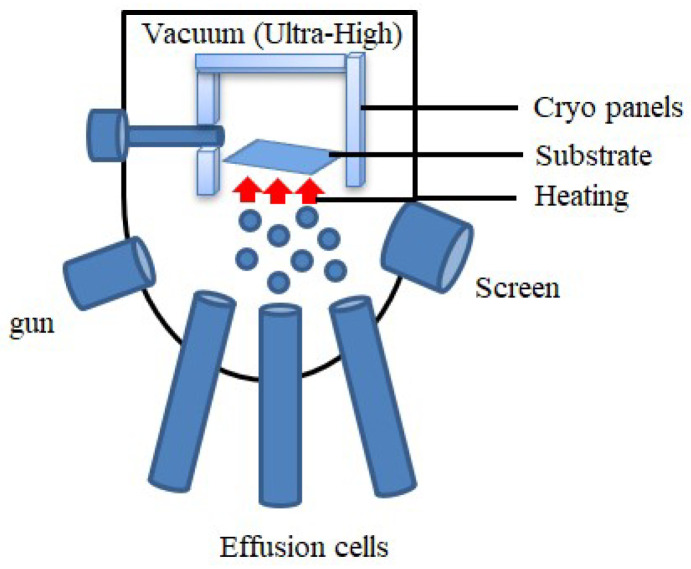
MBE technique.

MBE produces high-quality thin films where precise control over the material’s crystalline layers is crucial. It is one of the techniques developed in the late 1960s for depositing single crystals [65]. This technique is widely used for fabricating semiconductor devices, quantum dots, etc. This is one of the vacuum deposition processes where the source material is sublimated or evaporated, forming an atomic molecule beam and eventually condensing and forming a thin film. This process is unique because of its high vacuum condition. Typically, MBE contains effusion cells that act as a solid source of elements, as shown in Figure 4. As a result, a molecular beam or directed atomic beam is produced by the evaporation of the material in these cells due to heating. These beams move in an ultra-high vacuum chamber toward the heated substrate, making it possible to deposit thin films layer by layer precisely. A screen helps to focus the beams, while cryopanels condense stray molecules to maintain the ultra-high vacuum. The guns could represent sources for additional processing, such as ion beams for surface modification. This epitaxy process makes it convenient to form intricate heterostructures, and high-purity thin film is achieved [65]. MBE is ideal for producing complicated materials and heterostructures needed in cutting-edge semiconductor devices because of its high precision. On the other hand, thermal evaporation involves heating a substance until it evaporates and then condenses onto a substrate. Although less control over layer thickness and composition is available, this method is easier to understand and less complicated [66]. Thermal evaporation is frequently used for more straightforward coatings where exact control is less important. MBE offers more control and precision than thermal evaporation, which qualifies it for complex and high-end thin-film applications. Several studies [66,67,68] showed the fabrication of high-quality crystalline gallium arsenide (GaAs) doped with silicon thin films using the MBE process. This is completed by depositing individual Ga and As atoms layer by layer. These films are used in high-speed transistors, solar cells, and laser diodes.

##### Laser Evaporation (or Laser Ablation)

**Figure 5:**
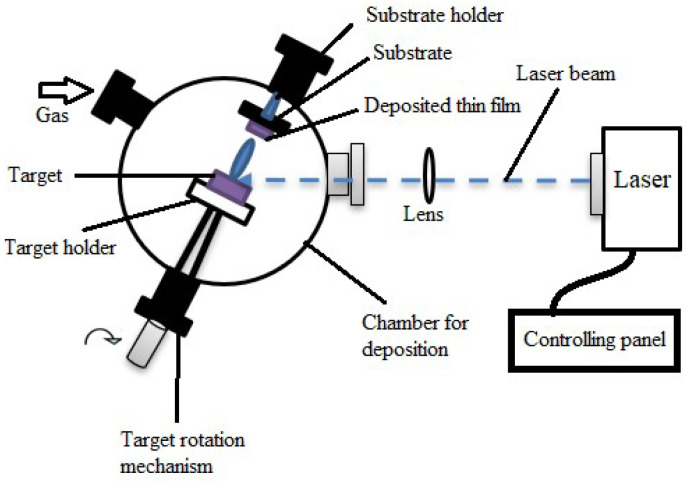
Schematic representation of laser ablation.

Laser evaporation/laser ablation is one of the most commonly used processes where precise stoichiometry and composition can be attained. This technique uses a high-energy laser to rapidly heat and evaporate the target material (primarily solid) to create vaporization, eventually forming the desired thin film. As the laser-induced vapor spreads away from the target, it produces a plume or cloud of atoms, ions, and clusters of material. This vapor plume is eventually directed toward the substrate so that it can produce the required thin film, as shown in Figure 5. Gas is frequently added to aid in the deposition process. To guarantee consistent ablation, the target is fixed on a holder that rotates. The substrate is placed before the target, where the thin film forms. A lens focuses the laser beam, and a panel controls the laser and other settings. Laser evaporation is conducted at a controlled temperature [66]. This process is used in many applications, such as semiconductors, devices, superconductors, electronics, and other advanced materials [69,70]. In the [68] study, laser ablation was used to deposit thin films of yttrium barium copper oxide, which is used in applications for high-temperature superconductors. We obtained high purity and good adhesion in materials from this technique. This technique fabricates thin-film solar cells where cadmium telluride (CdTe) or copper indium gallium selenide (CIGS) were used as deposition [71,72]. Some transparent conductive films (TCFs) are fabricated using these techniques and are used in applications such as touch displays. According to several authors [73,74,75,76], ITO thin films vastly used this technique for fabrication.

##### Resistance Heating Evaporation

**Figure 6:**
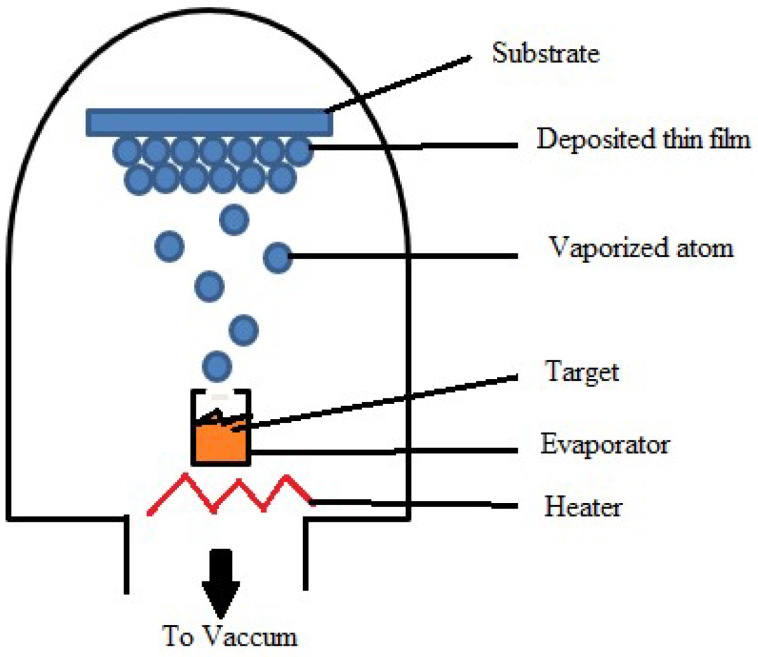
Resistance heating evaporation technique.

Another PVD process for creating thin films is resistance heating evaporation, where the material source (typically a wire) is heated resistively to evaporate and produce the thin film [72]. Another technique where rapid film growth is advantageous is the flash evaporation technique. This thin-film deposition technique is commonly combined with other deposition processes to obtain the desired thin-film properties [72]. However, in the case of the inductive evaporation technique, induction heating is used to evaporate the source element. This heating is generated using an induction coil that produces an alternating magnetic field. The source materials experience electric current due to electromagnetic induction as the coil is placed near the source material. This technique has precise temperature control and helps to obtain a quality deposited film. Figure 6 represents the resistance heating evaporation technique [73]. 

Table 2 below compares five distinct evaporation methods from PVD techniques, emphasizing features pertinent to the deposition of TCFs. This table also compares the suitability of five different deposition procedures for creating TCFs, considering substrate heating, vacuum requirements, uniformity, energy source, and deposition rate variables. Every method has benefits and drawbacks that vary based on the particular needs of the thin-film deposition application.

##### Ion Plating

**Figure 7:**
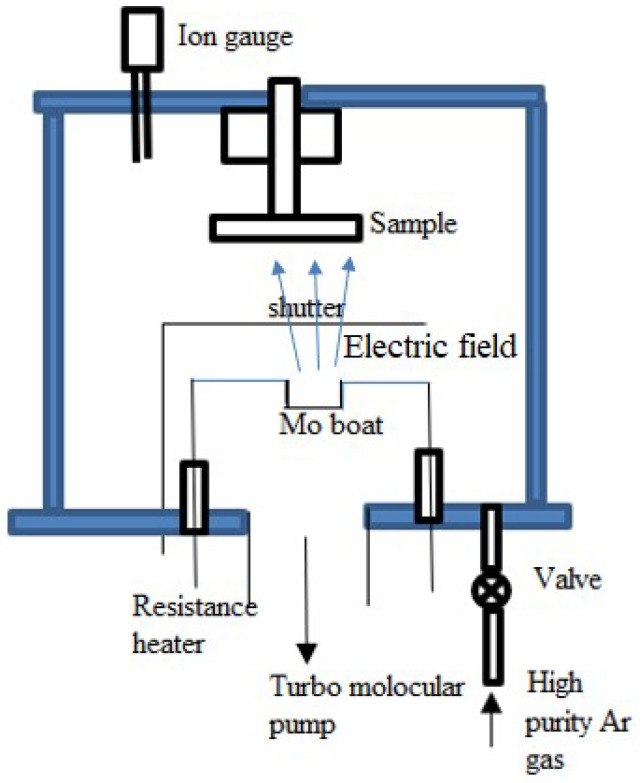
Ion plating deposition technique.

Ion plating, or ion-assisted deposition, vaporizes the target materials and directs high-energy ions toward them. Other methods, such as EBE, resistance heating, or sputtering, also vaporize these target materials, where a reactive gas is introduced to the evaporated material to ionize it, usually performed in a vacuum chamber. The ion plating method directs high-energy ions toward the vaporized target material [72]. The characteristics of the deposited thin film are altered, and the ion bombardment improves the deposition process. These ions move in the direction of the substrate by an electric field. Figure 7 shows that the source material is heated in a Mo boat using a resistive heater to evaporate, and the vaporized substance travels toward the sample (substrate). The procedure occurs in a vacuum chamber that is kept in check by an ion gauge and maintained by a turbo molecular pump. A shutter manages the deposition rate, and a valve facilitates the introduction of high-purity Ar gas to aid in the ionization process. When the ions collide with the evaporated material and substrate, they experience improved adhesion and cause reduced porosity and property changes [89]. As a result, a hard coating is formed as a thin film. This process is widespread when improved adhesion and wear resistance are critical. It can be used in cutting tools, coatings, and aerospace applications [90]. Several factors contribute to the thin films produced by ion-plating deposition. Better film adherence and more excellent density result from the process of acceleration of ions towards the substrate [91]. This process produces films with smooth surfaces and few imperfections by precisely controlling the thickness and homogeneity of the film. Furthermore, the deposited film’s electrical and mechanical properties can be further enhanced by the high energy of the ions by increasing its crystallinity. Because of these qualities, Ion plating is the best option for applications that call for strong, high-performing thin films.

In many studies, ion plating deposits thin films with enhanced properties. Different studies [92,93,94] showed the deposition of titanium nitride (TiN) coating using ion plating to improve hardness and wear resistance. These materials can be used as cutting tools and machine components as well. Other studies [95,96] show that it is possible to effectively construct a hard homogeneous TiN coating using ion plating on top of an intermediate alumina with a low coefficient of friction and better adhesion that provides excellent load support [93]. However, some studies [97,98,99,100] have used Au, which was synthesized using ion plating for decorative coating. Ion plating techniques are also used in synthesizing optical coatings, tribological coatings, medical devices, and aerospace coatings [90].

##### Sputtering

Among the most popular PVD methods used in thin-film deposition on substrates is sputtering. The fundamental difference between sputtering and evaporation is that sputtered methods have different particle emission kinetics than the other methods [101,102]. As discussed earlier, the evaporation methods are based on the evaporation conducted by applying heat to any specific macroscopic portion of the source material equal to or beyond the temperature of its melting. In this matter, sputtering is a non-equilibrium process at an appropriate energy range. A sputtering event occurs when a particle with high energy impacts a target, which is the surface. The target material is bombarded with energetic ions, which eject the target’s neutral atoms, molecules, any ion, or even photon, and a thin layer is deposited to a substrate [103,104,105,106]. When a high-energy particle collides with a surface, it can knock loose one or more atoms from the surface or just beneath, known as kinetic dislodgement. With immense kinetic energy from the first particle, these dislodged atoms go through the target material and knock out more atoms. The local temperature increases in phonon when the residual energy is absorbed [107]. The total kinetic energy is distributed among the target atoms, so the atoms are not displaced further. During the kinetic impact process, atoms that stay close to the surface can be dislodged with enough energy to easily exceed the surface binding energy, which can later be released from the target. These atoms are called sputtered atoms, and the process of their depositing is known as sputter deposition [108,109].

**Figure 8:**
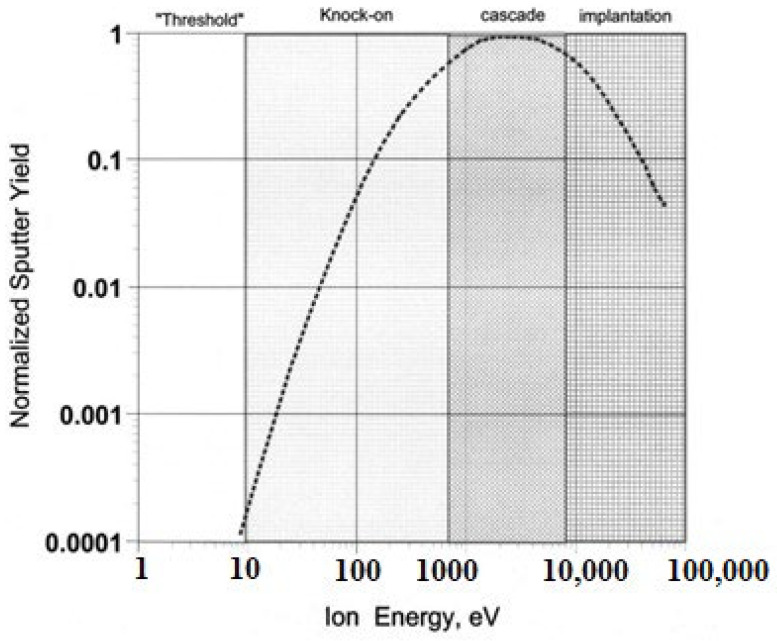
Physical sputtering’s energy regimes. Reproduced from [98] under permissions from copyright clearance center.

Many aspects of the resultant collision processes have been discussed in [98], which was determined by the incident particle’s mass and energy shown in Figure 8. We can see from Figure 8 that shifting too many atoms is impossible as there will not be enough energy at very low incidence energies. Initially, this was referred to as a sputter threshold, which is used to determine the lowest particle energy that could generate an atom that is released [99]. The development of the idea of this sputter threshold also contributed to the measurement of sputter yield, which fell off at energies in the tens of electron volts range and can be stated by Equation (4).

(4) $$\mathrm{Yield}=\frac{number\;of\;emitted\;atoms}{incident\;particle}$$

The idea of a sputter threshold was and continues to be widely accepted until very high-density plasma sources started to produce the evidence. It seemed like sputtering was occurring at energies well below the classical threshold. This was initially noted with electron cyclotron resonance plasma generators, which had metallic and dielectric components in their construction [110,111]. Following several minutes of operation at approximately one kilowatt of microwave power into the plasma, metallic coatings were produced on the dielectric surfaces within the source. These sources have easily measurable plasma potentials of just a few volts, but their ion currents are roughly 20 A/kW of incident microwave radiation [101]. As previously described, the impact point causes a rough sputtering process with a random collision sequence, resulting in atom emission near the surface, with incident energy in the range of 50–2000 eV [102]. This range, often called “collisional” or “knock-on” sputtering, is the foundation for nearly all thin-film applications. When the incident particle reaches higher energies between 2 and 50 keV, it generates a collision cascade close to the impact point, breaking all the bonds of many atoms and being nearly as dense numerically as a gas. The incident particle enters the target deeply before depositing energy at energies greater than 50 keV. The sputter yields drastically decrease when less energy is near the surface, and the process is better understood [98].

Sputtering is a prevalent technique used in many sectors to deposit thin films for magnetic storage devices, TCFs, optical coatings, semiconductor production, and other applications. It has benefits, including excellent purity, good adherence, and the capacity to deposit various materials [109,111]. Based on available resources, costing, purity required, characteristics, and more, several techniques are shown in Figure 1 to uphold sputtering.

##### Radio-Frequency (RF) Sputtering

**Figure 9:**
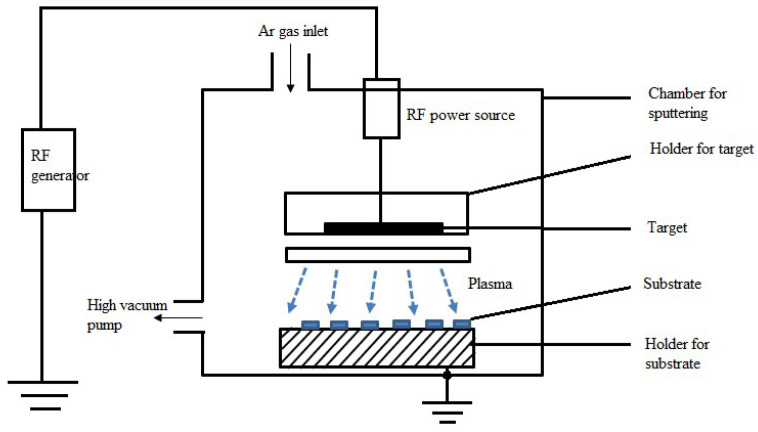
RF sputtering system’s working chamber.

RF sputtering is a prevalent sputtering process nowadays. This procedure involves applying a high-frequency electromagnetic field in a vacuum chamber to a target material. Direct current (DC) sputtering poses challenges if the target to be sputtered is insulating [112]. This is because maintaining discharge between the electrodes would require the application of an unusually high voltage (>10^6^ V). The typical voltage range for DC sputtering is 100 to 3000 V [113,114] Nevertheless, if a high-frequency lower voltage is provided, the anode and cathode alternately change polarity, resulting in enough ionization from oscillating electrons. Figure 9 shows the laboratory-scale RF sputtering system’s working chamber. Argon gas is introduced into the sputtering chamber via an Ar gas input during the RF sputtering process, where it ionizes. These ions hit the target material, pushing atoms out of it and onto the substrate below, which is kept in place by the substrate holding. The RF generator powers the RF power source, which creates an electromagnetic field across the target. A high vacuum pump keeps the entire system operating at a high vacuum to maintain a controlled environment. By applying an RF electromagnetic field across the target, insulating materials can sputter and block charge accumulation. In theory, electronics can be insulated and used at frequencies between 5 and 30 MHz. Nonetheless, as it is globally designated for this use and other frequencies are available for communication, 13.56 MHz is frequently utilized for deposition [104]. RF sputtering creates thin coatings with excellent adherence and homogeneity, making it possible to precisely manage the composition and thickness of the film, which is essential for modifying its characteristics. Smooth surfaces and small microstructures can be achieved in films produced by RF sputtering, which is especially useful for depositing films on insulating substrates. Low-temperature deposition is another feature of the technique that is advantageous for heat-sensitive materials. To attain the appropriate film properties, though, rigorous optimization is needed because factors like power, pressure, and gas composition can have an impact on the film quality. RF sputtering is another form of alternating current sputtering. When sputtering with AC power, an RF power source creates the alternating electric field that drives the process. In contrast, the target material is continuously exposed to a voltage in DC sputtering. After the atoms are ejected, they condense to form a thin layer on a substrate. This method can be used in insulating materials despite the meager deposition rate [115]. A DC-like diode result can be obtained from this technique when a magnetic field is used with this process in balanced and unbalanced configurations. This technique is vastly used in the electronic device fields, such as integrated circuits, transistors, etc. [116]. Many studies [117,118] have been conducted using RF sputtering thin films, which have been developed to enhance solar cell efficiency. However, RF sputtering can be used to create coatings for optical purposes, such as anti-reflective coatings on lenses or mirrors [12]. In microelectromechanical systems (MEMSs) and sensors, thin films like ZnO, which is produced by RF sputtering, are used [117,119]. Some studies used radio-frequency sputtering to create TCFs, such as ITO, for touchscreen and display applications. These illustrations highlight the wide range of uses for which RF sputtering is essential for creating thin films with the required properties [109].

##### Reactive Sputtering

**Figure 10:**
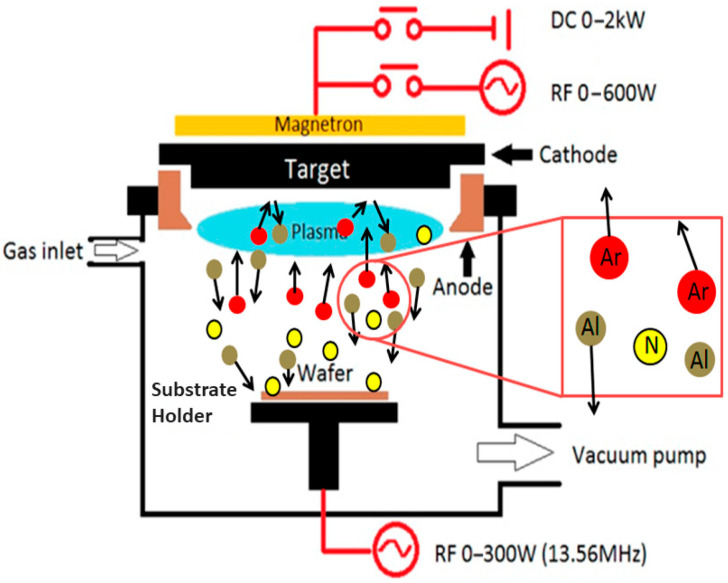
Reactive sputtering technique. Reproduced from [111] under permissions from copyright clearance center.

A target material is sputtered during the thin-film deposition while introducing a reactive gas known as reactive sputtering. Figure 10 shows the schematic diagram for the reactive sputtering technique. During sputter deposition, a reactive gas species in the chamber can integrate into the target material and deposited films, similar to reactive evaporation [98]. The latter has a quantifiable impact on film characteristics and significantly alters the nature of the deposition process. Reactive gases are introduced into the chamber via a gas inlet and ionized to generate plasma using the reactive sputtering technique. These ions attack the target material, expelling atoms as a result. The wafer (substrate), kept in place by the substrate holder, is coated with a compound created by these atoms’ subsequent reaction with the gas injected. A vacuum pump maintains the system under vacuum. The complete setup is connected to a magnetron that improves the ionization of the plasma. An RF power source (13.56 MHz and up to 300 W) and DC power (up to 2 kW) provide the energy to sustain the plasma and manage the sputtering process. Sputtered in an inert gas (like Ar), a pure metallic target is the simplest example of reactive sputtering. If a decent chamber base pressure and high-purity Ar are maintained, a deposition of metallic sheets of reasonable purity without adding any reactive gas species can be achieved. If a reactive species is subsequently introduced into the chamber, it has the potential to become embedded in the chamber walls, the growing films on the sample, and the metallic target [120]. Both RF and reactive sputtering yield superior thin films with outstanding adherence and homogeneity. Reactive sputtering, on the other hand, enables fine control over film composition by explicitly allowing the production of compound films by introducing reactive gases. Reactive sputtering is especially useful for producing films with specific chemical properties, providing more control over stoichiometry and material properties, even if RF sputtering is adaptable and efficient for various materials. Both techniques necessitate meticulous parameter tuning; nonetheless, reactive sputtering is recommended when precise compound synthesis is crucial. Some thin films produced using reactive sputtering are TiN for wear resistance and tungsten nitride (WN) for microelectronics [117,121].

##### Ion Beam Sputtering (IBS)

**Figure 11:**
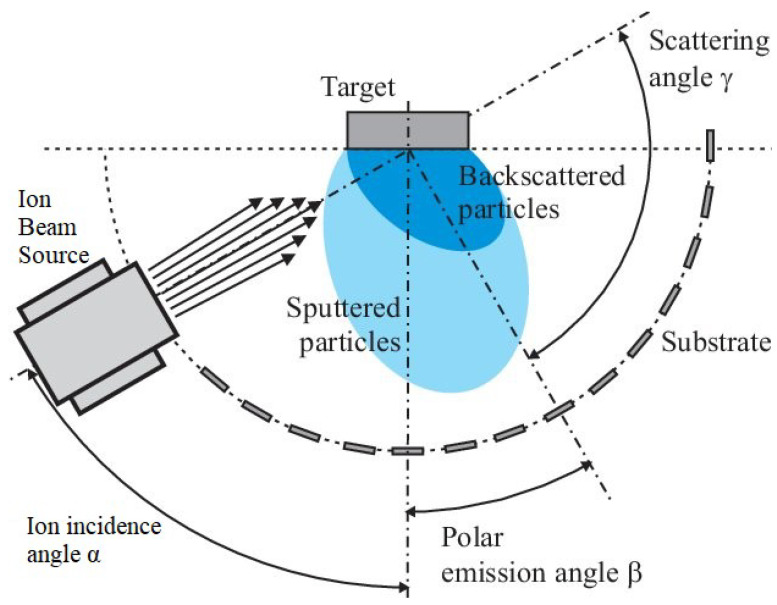
Setup for IBS deposition. Reproduced from [114] under permissions from copyright clearance center.

A material is sputtered off a target using an ion beam in the thin-film deposition technique known as IBS, and the deposited material is placed onto a substrate. This technology enables precise control over the composition and thickness of the film. This method forms the thin film by sputtering the target material, which can be dielectric or metallic, onto the substrate using an ion source. Because a monoenergetic ion beam is used during the sputtering process, the thin films produced are of excellent quality and possess extremely accurate thickness [112]. When the highest level of precision is needed, this procedure is frequently employed to create coatings for semiconductors and precision optics. A strong electric field is directed towards the surface of the substance to be vaporized by an ion beam. This results in ionizing the metal vapor vapors, whose momentum is transferred and then guided toward the desired production portion [113]. Multilayer optical coatings for beam splitters, interference filters, and anti-reflective coatings are frequently deposited by ion beam sputtering. Numerous optical and electrical applications make use of these films. Ion beam sputtering can create metallic thin coatings such as Ag, Au, and Al [112]. Applications for these films can be found in sensor technology, electronics, and optics. Using ion beam sputtering, thin coatings with magnetic characteristics such as iron (Fe), nickel (Ni), and cobalt (Co) can be deposited. These films have applications in magnetic sensors and storage systems. Moreover, ion beam sputtering is advantageous for various materials science, optical, and electronics applications because of its precision, controllability, and homogeneity [122,123,124]. A schematic diagram for this process is shown in Figure 11. Using an ion beam source, ions are directed toward a target at a particular ion incidence angle in the IBS process. Atoms from the target are released as sputtered particles upon collision, and these particles land on a substrate. Backscattered ions are also present. The procedure involves the backscattered ions’ scattering angle (*γ*) and the polar emission angle (*β*) for the released particles. Tailored thin-film deposition on the substrate is made possible by accurately managing these angles. Comparable to reactive sputtering, ion beam sputtering yields thin coatings with remarkable accuracy and control over composition and thickness. Although both techniques produce films with smooth surfaces and excellent uniformity, ion beam sputtering offers much more control over film qualities because of the directed ion beam, which makes it the best choice for applications needing films that are highly uniform and devoid of defects. Reactive sputtering works well for compound film generation, whereas ion beam sputtering works better to create extremely thin, precisely controlled layers [124].

##### DC Diode and Triode Sputtering

**Figure 12:**
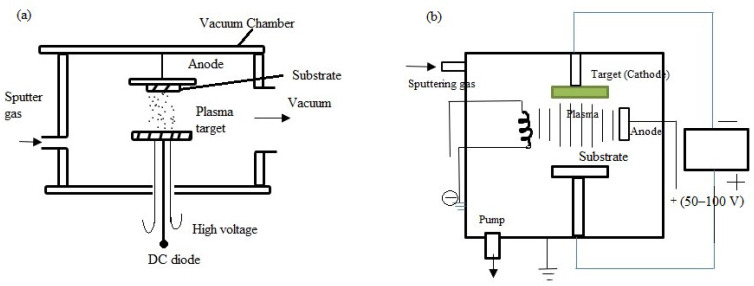
(**a**) Diode sputtering; (**b**) triode sputtering.

In DC triode sputtering, the target, the substrate, and an extra electrode (the triode electrode) are the three electrodes utilized. The triode electrode, the third electrode in DC triode sputtering, is essential to improving the sputtering process. This electrode, placed in the vacuum chamber between the target and the substrate, aids in controlling ionization and enhancing plasma density. The triode electrode attracts electrons and aids in maintaining a stable plasma by providing a positive voltage, which raises the sputtering gas’s ionization. As a result, the target material sputters more effectively, the film quality improves, and the deposition process is easier to manage. Conversely, two electrodes are used in DC diode sputtering: the substrate and the target. Because there is not a third electrode, the configuration is more straightforward and appropriate for some uses [115]. Figure 12 shows the schematic representation of both DC diode and triode sputtering techniques. A high voltage is placed between the anode and the plasma target (cathode) in a vacuum chamber filled with sputter gas using the DC diode sputtering technique (Figure 12a). The gas is ionized by this voltage, which produces a plasma that bombards the target and ejects atoms that fall onto the substrate. Using a pump to maintain the vacuum and an extra electrode (the anode) to increase plasma density, the DC triode sputtering technique (Figure 12b) enables more effective sputtering onto the substrate. A vacuum chamber is used in both configurations to regulate the thin-film deposition environment. The DC triode sputtering is used in thin films, such as oxides with transparent conductivity, such as ITO, employed in electronics [116]. Additionally, metal films have a range of uses, including Cu and Al. However, both methods can be implemented in many thin films, for example, Al, Cu, titanium (Ti), and germanium (Ge). These are used in solar cells, optoelectronics, electronics, and other sectors. The film material selected is determined by the intended use and the specifications needed to build the device or component. A magnetic field can be introduced to the vicinity of the cathode to address the inherent shortcomings of both DC and RF diodes [117]. Similar to RF sputtering, DC diode and triode sputtering yield thin films with good uniformity and adherence, but they generally work better with conductive materials. While triode sputtering provides more control over ionization and film characteristics, DC diode sputtering is more straightforward and efficient for simple applications. DC techniques are effective for producing conductive films where complicated stoichiometry is not needed. However, they are less exact in controlling film composition and structure than reactive and ion beam sputtering.

##### Magnetron Sputtering

**Figure 13:**
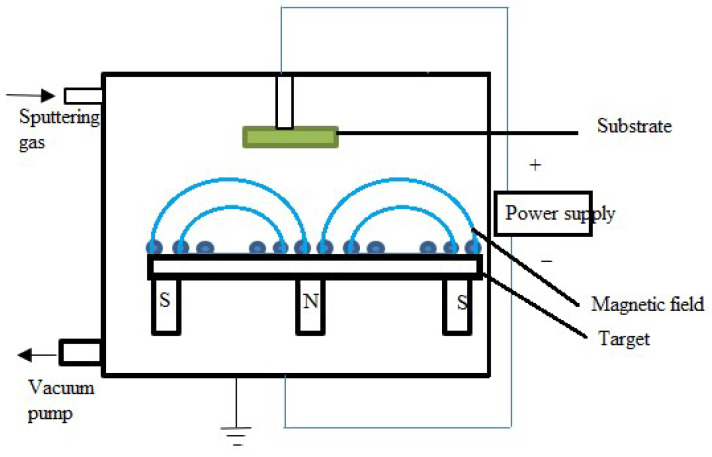
Magnetron sputtering technique.

In the magnetron sputtering process, positively charged ions from the plasma are propelled toward a target material in a vacuum chamber. This plasma is generated by combining electric and magnetic fields, which is more efficient than DC diode and triode sputtering. Electrons are accelerated toward the target by an electric field created when a voltage is introduced between the target and the chamber walls. In the meantime, a magnetic field produced by magnets behind the target captures and concentrates these electrons, increasing the sputtering gas’s ionization and forming a dense plasma. With more efficient control and increased sputtering efficiency, this technique outperforms DC diode sputtering, which requires higher voltages to insulate targets, and triode sputtering, which adds a second electrode to increase plasma density. These ions dislodge atoms from the target material upon collision, and the atoms deposit onto a substrate to produce a thin film, as shown in Figure 13. A sputtering gas—typically argon—is introduced in the chamber and kept under vacuum by a vacuum pump. N and S, the magnets below the target (source material), apply a magnetic field (shown by blue lines) that increases the plasma density near the target. Ejecting atoms onto the substrate (highlighted in green), the power supply generates an electric field that induces ions in the plasma to hit the target. A magnetron is a unique instance of magnetic plasma enhancement in which the magnetic field is set up so that the electrons’ E × B drift paths, also called the Hall effect, form a closed loop. The same closed-loop phenomenon underlies a variety of geometries, such as planar, conical, cylindrical, and hollow shapes [118]. Planar cases are the most prevalent in which a transverse B field is distributed throughout the cathode surface. At the cathode surface, this field usually has a strength of a few hundred gauss. The closed-loop path in the circular-planar geometry is a broad, circular, co-axial band close to the magnetron surface, and the B field is radial. The B field in the rectangular-planar device has a long rectangular drift loop tangential to the surface [118,119]. For 300 mm wafer deposition, circular-planar cathodes typically reach their maximum diameter of 45–50 cm, while rectangular targets with lengths of several meters have been built [120,121]. Metal thin films like Au, Ag, Cu, and Al are frequently deposited by magnetron sputtering [122,123,124]. In addition to magnetron sputtering, it is a technique that can be used to deposit thin films of ITO. This method can create insulating or dielectric films [125,126]. Magnetron sputtering effectively produces thin films with magnetic properties, such as those in magnetic storage media. Materials such as ITO, utilized in solar cells and touchscreens, are examples of TCO films [127]. Magnetron sputtering is comparable to RF sputtering but offers better ionization and target usage control. It yields thin coatings with excellent homogeneity, outstanding adhesion, and efficient deposition rates. By enabling lower substrate temperatures, the thermal stress that materials experience is lessened. Magnetron sputtering is superior to reactive and ion beam sputtering in terms of efficiency and scalability, which makes it perfect for coating vast areas while preserving high film quality. On the other hand, similar to reactive sputtering, it could take more intricate setups to obtain accurate control over film composition. Table 3 provides a comprehensive comparison of various thin films, focusing on their respective thin-film quality.

#### 2.1.2. Low-Pressure Chemical Vapor Deposition (LPCVD)

**Figure 14:**
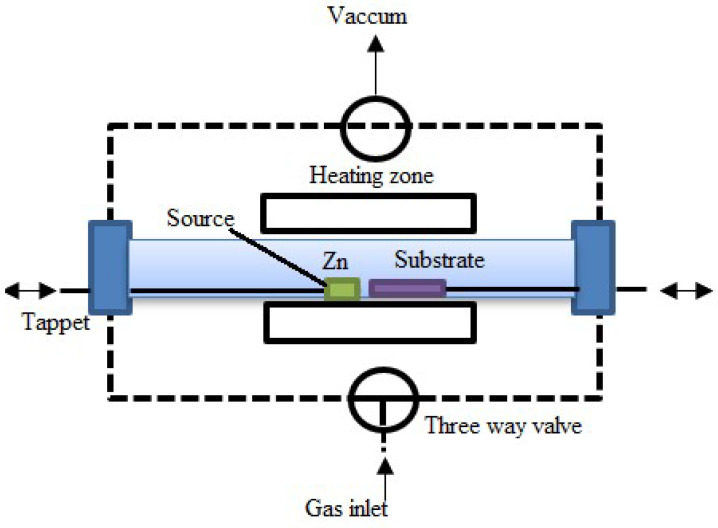
LPCVD system for ZnO nanowires.

With low pressure in a high vacuum environment, low-pressure CVD, also known as LPCVD, deposits thin films based on the absorption of precursor solutions and subsequent surface reactions. This CVD process differs from conventional CVD methods as it requires low pressure. The deposition chamber in LPCVD is usually kept between a few millibars and a few hundred millibars. This alteration makes it possible to form high-quality thin films, which also offers advantages in terms of control over the deposition process. This technique adds gaseous precursors to a deposition chamber, frequently as volatile chemicals or metal–organic compounds. A substantial drop in air pressure occurs in the chamber pressure. This low-pressure setting improves consistency in the deposition process and aids in reaction control. In the gas phase, the precursors undergo chemical reactions that produce reactive species. A thin film forms when reactive species are deposited on a substrate. The substrate is typically set up on a heated stage to regulate the deposition temperature. By reducing the pressure, the kinetics of the chemical reactions may be better controlled, which improves the uniformity and quality of the film. Figure 14 represents the schematic diagram for this process. Precursor gases are introduced into a temperature-controlled heating zone via a gas inlet in the LPCVD system for ZnO nanowires. ZnO is created when the heated Zn source material vaporizes and reacts with the gases. This substance settles on the substrate inside the chamber, close to the Zn source. A three-way valve-operated vacuum pump is used to maintain the system under vacuum.

The LPCVD-prepared perovskite thin film, with its long carrier diffusion length, improved crystallinity, increased stability, and strong absorption propensity, is a significant development. Luo et al. initially used the LPCVD technique in 2015 to create the perovskite thin film [130]. In the semiconductor industry, thin films are frequently deposited on silicon wafers using LPCVD to construct different layers in integrated circuits [131]. This underscores the importance of LPCVD in the industry. Thin-film solar cells, one type of photovoltaic device, are created using this technique [122]. Applications like protective layers and optical coatings that call for exact control over film properties are a good fit for LPCVD [123]. High-quality thin films with outstanding homogeneity, conformality, and controlled thickness are produced via LPCVD. It works especially well for uniformly covering complex geometries and laying coatings over them. Lower pressures during operation improve film density and minimize flaws, which makes the process perfect for applications needing accurate and dependable thin films.

Compared to certain other CVD methods, LPCVD offers advantages in film homogeneity, step coverage, and the capacity to deposit films at lower temperatures [132]. It plays a crucial role in the creation of electrical and photonic devices, demonstrating its significance in the industry. However, typical results of LPCVD are high residual stresses and gradients, which can harm MEMS devices.

#### 2.1.3. Plasma-Enhanced Chemical Vapor Deposition (PECVD)

PECVD, a technique that uses plasma to alter and improve the chemical processes during deposition, is a variation of CVD. In PECVD, the precursors are energized and activated by plasma, enhancing the characteristics of the film. This low-temperature vacuum thin-film deposition method holds a significant place in the semiconductor industry because it can coat surfaces where more traditional CVD methods may not be capable of tolerating the temperatures. The unique role of plasma in PECVD is a fascinating aspect that sets it apart from other deposition methods.

In traditional CVD, heat is applied to the substrate to be coated or to the immediate vicinity of the substrate to initiate chemical reactions. In the deposition chamber, precursor reactive gases are introduced. These gases react with the surfaces to be coated instantly or combine to generate new compounds that develop into films on the substrate surface. In PECVD, gaseous precursors are added to a deposition chamber, frequently in the form of inorganic or organometallic compounds. A plasma is produced inside the chamber using various techniques, including RF or microwave energy. Ions, electrons, and radicals are among plasma’s highly energetic and reactive species. Precursors are broken down into reactive species by the plasma energy, which enables more efficient and controlled chemical reactions. After undergoing gas phase chemical reactions, the activated precursors deposit onto the substrate material. The reactive species produced in the plasma produces a thin layer on the substrate. The substrate is frequently heated to improve adhesion and regulate the film’s characteristics.

**Figure 15:**
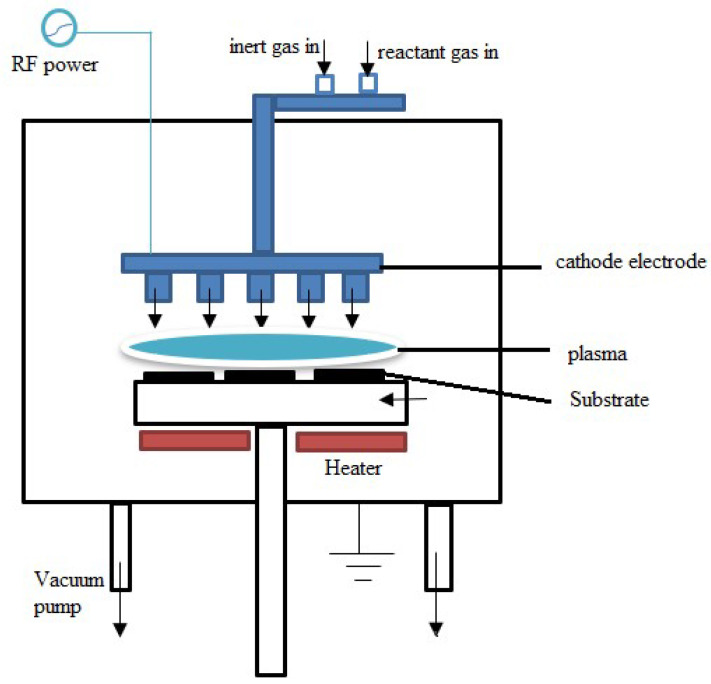
PECVD process.

PECVD is a vacuum-based technology that can handle relatively low substrate temperatures of ambient temperature to 350 °C because it is frequently conducted at pressures lower than 0.1 Torr [124]. PECVD makes stronger bonding possible, enabling substrates to be coated at lower temperatures while putting less stress on the thin-film interfaces. This is made possible because some of the energy needed for these deposition reactions is provided by plasma, as compared to the substrate that requires heating to extremely high temperatures to drive these reactions [125]. PECVD is frequently used in the semiconductor industry to deposit thin films to create integrated circuits and microelectronic devices. It is used to create a variety of coatings, including coatings on polymers or glass that reduce reflection. Thin-film deposition for solar cells, display technologies, and other electrical and optoelectronic devices is accomplished by PECVD. Moreover, with its adaptability and ability to regulate film properties precisely, PECVD is a technology that may be applied to various materials science and device fabrication applications [126]. Figure 15 depicts the working principle for the PECVD technique. In using PECVD technology, the precursors for the thin film that will be deposited onto the substrate are the reactant gases brought into the chamber and referred to as the source. These source gases enter the chamber through the reactant gas line at the top. The reactive species from ionizing the plasma gases are subsequently deposited onto the substrate. The substrate, placed on a heater at the bottom of the chamber, and the cathode electrode form a plasma when an RF power source stimulates the gases. A vacuum pump maintains the system under vacuum. The deposition process depends on the RF power’s electric field to keep the plasma active.

PECVD is appropriate for materials sensitive to temperature since it permits deposition at lower temperatures than some conventional CVD techniques. PECVD operates between ambient temperature and 350 °C, whereas traditional CVD operates between 600 °C and 800 °C [127]. This enables practical applications in scenarios where higher CVD temperatures could harm the coated device or substrate. Working at a lower temperature reduces tension between thin-film layers with varying thermal expansion/contraction coefficients. This technique makes high-quality bonding and high-efficiency electrical performance possible [127]. On complicated surfaces, the use of plasma aids in improving step coverage. On the other hand, since PVD coatings are a line-of-sight deposition, if imperfections protect areas from coating, there may be more significant variations in thin-film depth. Because the plasma stream may encircle the substrate, PECVD significantly lessens the line-of-site problem for high-conformance thin-film applications [127]. The plasma’s reactive species and activated precursors produce films with stronger adhesion and denser structures. Compared to LPCVD, PECVD generates thin films with better homogeneity and permits lower deposition temperatures, making it appropriate for sensitive substrates. By raising the reaction rate, the plasma augmentation results in improved film adhesion and controlled composition, which enhances film quality. While PECVD offers greater material choice freedom and is best suited for applications where processing at lower temperatures is critical, LPCVD offers superior conformality and film density. However, for best utilization, it is essential to consider application-specific needs. PECVD has advantages in thin-film deposition but also has problems, such as complicated equipment, probable precursor degradation, and difficulties establishing uniformity [126].

#### 2.1.4. Hot-Wall and Cold-Wall CVD

CVD techniques with “hot wall” and “cold wall” refer to differences in the reactor’s architecture and arrangement that impact the system’s temperature distribution. These words refer to the heating and cooling processes used on the reaction chamber walls.

A hot-wall CVD system evenly heats the reaction chamber’s walls and interior. The chamber is filled with the substrate, and high temperatures throughout the reaction encourage the necessary thin film to be deposited on the substrate. Because of its ease of use, hot-wall CVD is frequently employed in research and development environments [128]. Only the substrate is heated with a cold-wall CVD system; the reaction chamber walls are maintained at a lower temperature. The heated substrate is the primary site of the chemical reactions leading to film deposition when the reactant gases are injected into the chamber. Cold-wall CVD offers improved control over the deposition process by reducing undesired reactions on the chamber walls [129]. Several variables influence the decision between hot-wall and cold-wall CVD, including the materials being deposited, the required film qualities, and the overall process requirements. Each arrangement is beneficial and appropriate for various thin-film deposition applications [130].

The selection between hot-wall and cold-wall configurations is contingent upon various aspects, including the particular elements to be deposited, the film’s intended qualities, and the process’s overall requirements. These procedures are frequently employed in the following thin-film applications:

1. Industry of semiconductors: The semiconductor industry uses both hot-wall and cold-wall CVD to create thin coatings on silicon wafers. Hot-wall CVD is frequently utilized due to its ease of use and adaptability. Cold-wall CVD is used when exact control over film characteristics and little contamination from undesired reactions on chamber walls are essential [130].
2. Solar energy photovoltaics: Solar cells and gadgets cannot be produced without thin sheets. Layers can be deposited using hot- and cold-wall CVD techniques to fabricate solar cell structures [131].
3. Optical layers: Lenses, mirrors, and other optical components can have optical coatings applied via hot-wall and cold-wall CVD. These coatings can improve optical elements’ transmissive or reflective qualities [132].
4. Protective coatings: Electrochemical vapor deposition is used in both hot-wall and cold-wall applications to provide barrier coatings that shield electronic equipment from moisture and oxygen. Hot-wall and cold-wall CVD are crucial to creating thin films for energy, optical, and electronic-related uses. The demand for temperature control, the quality of the film, and specific material requirements all influence the choice between various designs [132].

#### 2.1.5. Atomic Layer Deposition (ALD)

A more precise kind of CVD is ALD. Both processes use chemical reactions to produce thin films, whereas ALD uses surface-controlled responses instead of flux-controlled reactions. This method involves depositing ingredients on the substrate surface layer-by-layer as single-atom films based on chemical vapor phases. Distinct substance components are deposited on the substrate surface using one or more precursor chemicals. Every precursor forms a monolayer of material by saturating the surface [133].

ALD’s growth principle is comparable to conventional CVD. During the deposition process, reaction precursors are deposited alternately, resulting in one layer of atoms deposited per reaction. Each new layer of atoms undergoes a chemical reaction directly linked to the previous layer. Consequently, precise control over layer thickness can be obtained by adjusting the number of deposition cycles [134]. The initial precursor gas enters the substrate and reacts chemically or adsorbs onto the substrate surface. By using an inert gas, the residual gas is flushed. A chemical reaction using the first precursor gas adsorbed on the substrate’s surface or a subsequent response using the byproducts of the first precursor’s reaction with the substrate can create the coating. Once more, inert gas is used to flush away the extra gas [129]. By regulating the reaction cycle, the thickness of the substrate can be readily and precisely controlled, and the film’s thickness can be as fine as an atom’s thickness. ALD can deposit a film uniformly on a surface that resembles a concave, giving the film the same shape as the original substrate [134]. The unique benefits of ALD technology are uniform three-dimensional films, consistent shape and originality, and conformability. The pinhole-free character of the film is determined by the bottom-up growth mechanism and pinhole-free surface, making it useful for passivation and blocking applications [135].

These techniques must have low thermal budgets, high film thickness accuracy, and excellent conformality on three-dimensional structures. Nevertheless, the conventional PVD and CVD methods have yet to adapt entirely to this trend. Because of its high controllability of deposition parameters (thickness, composition, and structure), outstanding homogeneity, and conformality, ALD technology has many applications in micro- and nanoelectronics and nanomaterials [136].

The deposition process’ sequential, self-saturating, gas-surface reaction control is the source of ALD’s main benefits. When selecting ALD over alternative deposition methods like CVD or sputtering, the conformality of the films produced by ALD is frequently the deciding factor [137]. However, when the precursor pulse times are long enough, it can spread into deep pits, enabling complete surface response. On high-aspect-ratio structures, subsequent cycles enable uniform development, while CVD and PVD may experience non-uniformity due to quicker surface responses and shadowing effects, respectively [138]. Another obvious benefit of ALD is regulating the thin-film thickness after deposit. The number of ALD cycles in layer-by-layer deposition enables a film’s thickness customization [139]. The growth per cycle of numerous ALD films has been compiled in earlier reviews and, depending on the specific technique, is usually less than one Å/cycle [140]. Composition management is one of ALD’s other prominent benefits. Materials like zinc tin oxide (ZTO) and strontium titanate (SrTiO_3_), among many others, have been used to demonstrate composition control [141,142,143].

#### 2.1.6. Pulsed Laser Deposition (PLD)

A pulsed laser is used to ablate (remove) material from a target, which is deposited onto a substrate to produce a thin film. This process is known as PLD. This technique creates the target material’s required composition for the thin film. A high-purity solid that serves as a representation of the material to be deposited is often the target. The PLD procedure is conducted in a vacuum chamber to reduce atmospheric gas influence [142]. The substrate is meticulously prepped and placed inside the vacuum chamber to receive the thin film. The target material’s surface is exposed to a high-energy pulsed laser beam. A plume of vaporized material is produced due to the target being rapidly heated and ablated by the powerful laser pulse. A plume of the vaporized material forms and spreads outward from the target. Ions, atoms, and target material clusters comprise this plume. The material that has evaporated and condensed to form a thin film on the substrate surface is guided toward the substrate by the plume [78]. As the process proceeds, layer by layer, the thin film grows. Film growth kinetics depend on several variables, including substrate conditions, target characteristics, and laser parameters.

**Figure 16:**
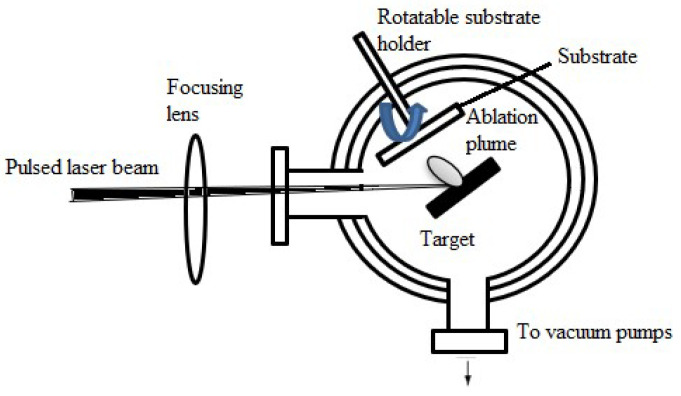
PLD process.

Because PLD replicates the desired material composition precisely, it provides precise control over the composition of the deposited thin film [78]. Vacuum environments reduce contamination and produce thin films with excellent purity. Because of its versatility, PLD may deposit many different types of materials, including metals, nitrides, oxides, and complex compounds [143]. Depositing high-temperature superconducting thin films using PLD is common [78]. Semiconductor thin films for electronic purposes can be deposited using PLD. Thin films with optical characteristics, including anti-reflective coatings, are created using PLD [144]. PLD can be used to deposit magnetic thin films for use in sensors and data storage [78]. Figure 16 shows the schematic of a PLD deposition system. A pulsed laser beam is focused onto a target material inside a vacuum chamber using a focusing lens in this process. After the laser energy ablates the target, a plume of vaporized material is produced and deposited onto the substrate, which a rotating substrate holder supports. Vacuum pumps are attached to the system to sustain the required vacuum levels for accurate film deposition. These essential elements and their functions during the deposition process are depicted in Figure 16.

### 2.2. Solution-Processed (Non-Vacuum) Depositions

Liquid-based precursors are applied to a substrate, usually at atmospheric pressure, in solution-processed (non-vacuum) deposition processes. These techniques are easy to use and reasonably priced, making them perfect for creating thin layers on intricate surfaces or over large regions. Despite being generally less precise than vacuum-based techniques because they are simple and scalable, solution-processed methods are frequently employed in organic electronics, photovoltaics, and coatings [145].

#### 2.2.1. Atmospheric Pressure Chemical Vapor Deposition (APCVD)

**Figure 17:**
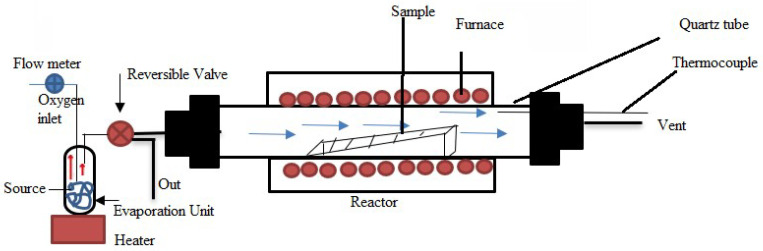
Schematic representation of APCVD technique.

APCVD, known as atmospheric pressure chemical vapor deposition, forms thin films at atmospheric pressure. In this procedure, precursor gases are injected into a substrate-containing chamber, and a chemical reaction starts to deposit a thin layer on the substrate surface, as shown in Figure 17. Gas is introduced into the evaporation unit via an oxygen input, where it combines with other precursors. A reversible valve is then used to guide this mixture into the reactor. The material is deposited onto the substrate due to a reaction in a quartz tube within the furnace containing the sample (substrate). The gases are vented out while the thermocouple monitors the temperature. The evaporation unit’s precursor material serves as the source in this configuration. Because the procedure is carried out at atmospheric pressure, it is easier to use and less expensive than many other deposition techniques that call for vacuum settings [146]. Applications for APCVD are widespread in several industries, including material synthesis, coatings, and semiconductor production.

The earliest and most used technique for thin-film deposition is atmospheric-pressure CVD from hydrides, which uses N_2_-diluted SiH_4_, B_2_H_6_, PH_3_, and O_2_ at temperatures between 240 and 500 °C [74]. This technique is commonly used in sectors like semiconductor production, where integrated circuits and other electronic devices are created depending on thin films. Because of its affordability, ease of use, and capacity to deposit films over vast regions, APCVD is preferred [147]. Applications for the method include coating, optical films, and other thin-film technologies. It can be applied to a variety of materials. However, because of the increased pressure and less control over the deposition environment, APCVD films may have more flaws and a lower density than LPCVD films. While APCVD is frequently utilized for less essential applications where speed and simplicity are more important than the most excellent film quality, LPCVD excels in quality, with superior conformality and fewer flaws.

#### 2.2.2. Chemical Bath Deposition (CBD)

**Figure 18:**
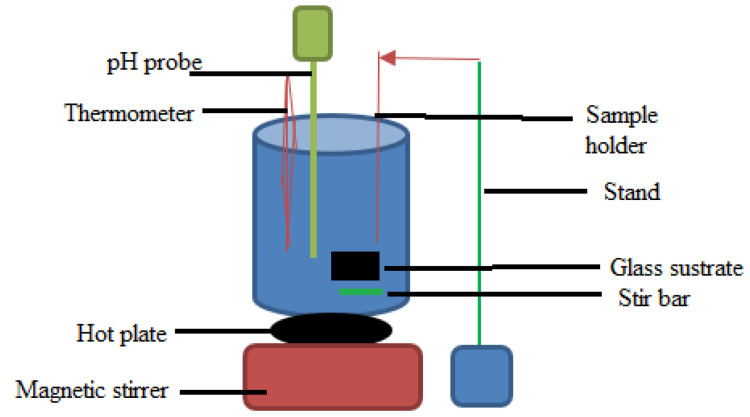
An illustration of the CBD process.

The CBD technique is a solution-based method where a substrate is submerged in a chemical bath that contains precursors for the substance that needs to be deposited. This technique dissolves metal salts or precursors in a solvent to create a chemical bath. A glass substrate is submerged in a solution in a beaker set on a heated plate using the CBD method, as shown in Figure 18. A thermometer is used to track the temperature of the solution, and a pH probe is used to detect its pH. The solution is uniformly mixed thanks to the magnetic stirrer under the beaker, and the stir bar within the beaker helps with the stirring action. The substrate is held in place by the stand and sample holder. The material will settle out of the solution and be placed on the substrate. The chemical solution, which includes the thin film’s precursor materials, is the source. The intended thin film’s material composition determines which precursors to use. The substrate is submerged in the chemical bath; this substrate is usually a solid surface or one with a seed layer already applied. The thin layer nucleates and grows due to chemical reactions on the substrate’s surface [148]. Typically, the concentration of the precursors, pH, and temperature are changed to regulate the reactions. As the reactions proceed, the thin film expands on the substrate layer by layer. The deposition conditions can be altered to monitor and regulate the growth process. For some applications, CBD is appropriate because it may offer good consistency over large-area substrates. CBD frequently deposits different substances, including metal oxides, sulfides, and chalcogenides [149].

CBD is the most straightforward chemical thin-film deposition technique. These procedures require a substrate to be de-deposited and a vessel to hold the solution, often an aqueous solution of common chemicals. Sulfuride can be generated gradually in the deposition solution to achieve this rate control [150]. Most CBD reactions have occurred in alkaline solutions, even though CBD can react in both acidic and alkaline solutions. Compared to other deposition procedures, CBD is frequently carried out at very moderate temperatures, making it appropriate for heat-sensitive substrates. Applications for CBD include anti-reflective coatings, sensors, solar cells, and TCFs [148]. Applications for ZnO thin films produced with CBD include transparent conductive coatings for optoelectronic devices, including screens and solar cells [151]. The potential use of CBD-deposited copper selenide thin films in thermoelectric and solar devices has been studied [152]. Due to their unique electrical qualities, PbS thin films created by CBD are used to create infrared detectors and sensors. CBD methods can deposit CuInSe_2_ thin films, frequently utilized in thin-film solar cells. Applications in optical coatings have been investigated for SrCO_3_ thin films generated by CBD. These illustrations demonstrate how versatile CBD is when depositing thin films for various uses, such as energy storage, sensors, solar energy, and optoelectronics. The material selection for a given application is contingent upon the intended characteristics and capabilities of the thin film.

#### 2.2.3. Spin-Coating

**Figure 19:**
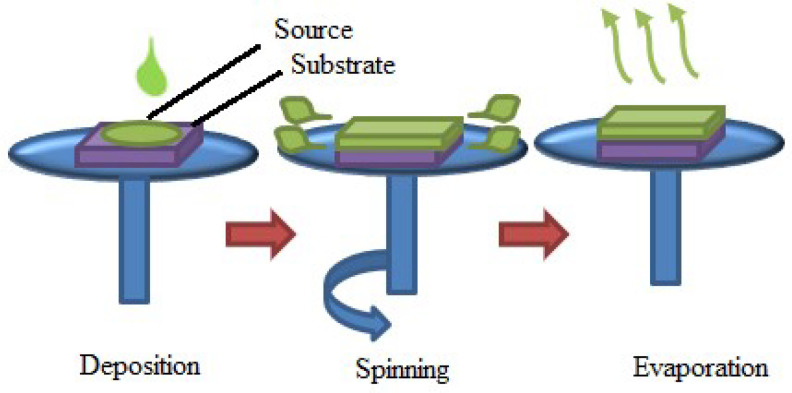
Spin-coating deposition technique.

Uniform thin-film applications to flat substrates are accomplished using spin-coating. Usually, a small amount of resin is deposited in the center of a substrate, which is then spun at high speeds, ideally around 3000 RPM [153]. Due to centrifugal force, the resin will gradually spread to the substrate’s edge and peel off, leaving a thin layer of resin on the outside. The final film thickness and other attributes are determined by resin type, drying rate, particle percentage, surface tension, different characteristics, and the spin process parameters. A few examples of the variables that affect the properties of coated films are acceleration, fume exhaust, and final rotational speed [154]. Figure 19 shows the spin-coating process. During the deposition stage of spin-coating technology, a liquid source material is coated with a substrate. Next, the substrate is quickly rotated (spun), which uses centrifugal force to disseminate the liquid evenly throughout the surface. A thin homogeneous layer is left on the substrate by the liquid’s solvent evaporating during the evaporation stage. These steps are depicted in the illustration, where the liquid drop serves as the source material, the substrate acts as a platform for the liquid’s deposit, and neither electric nor magnetic fields are used during the process.

The substrate, often a smooth, flat surface like a glass slide or silicon wafer, is properly cleaned to eliminate impurities. The substance to be deposited is added to a solution. Usually, this solution is a liquid that has the precursor components suspended in a solvent. A tiny amount of the solution is dropped into the middle of the substrate. After that, the substrate is quickly spun on the spinning chuck. The liquid spreads outward due to the centrifugal force, covering the substrate in a thin homogeneous layer. A thin layer of the intended material is left behind as the solvent in the solution evaporates during the spinning process. The spun film is let to dry thoroughly, and further procedures like annealing could improve the thin film’s qualities even more.

The spin-coating can create highly uniform thin layers across a substrate. The method can be used for high-throughput production because it is comparatively quick. It can be applicable to numerous materials, such as inorganic materials, organic molecules, and polymers [155]. Variations in spinning speed, solution concentration, and viscosity can all be undertaken to regulate the final film’s thickness. Spin-coating is widely utilized in microelectronics, optics, and the semiconductor industry for integrated circuit manufacture, thin-film deposition, and photoresist coating. Repeatability is one of the most crucial aspects of spin-coating [154]. Significant changes in the coated film can arise from subtle changes in the parameters defining the spin process. Although there have been few studies on the spin-coating technique, it does have some advantages, including low cost, manageable control and handling of chemicals and substrates, and the ability to fabricate thin films more quickly and with less vigor at high annealing temperatures [155]. 

During photolithography, photoresist materials are typically spin-coated onto semiconductor wafers [156]. This facilitates the pattern-making process on the wafer used to fabricate integrated circuits. Polymer thin films, which are utilized in organic electronics, sensors, and protective coatings, are frequently deposited using spin-coating. Organic layers are spin-coated onto substrates to form thin films used in OLED production. This is essential for the advancement of OLED device emissive layers [154]. Spin-coating is frequently employed to deposit perovskite materials used in solar cell applications across substrates to produce thin homogeneous layers that facilitate effective light absorption [153]. Spin-coating is used in bioelectronics to deposit sensing layers for biosensors or thin films of biomaterials. The spin-coating can be applied to optical surfaces, including lenses or camera sensors, to deposit thin films for anti-reflection coatings. Spin-coating can produce thin homogeneous films on substrates for electrolyte materials used in fuel cells or battery components [25]. These illustrations demonstrate the adaptability of spin-coating to a broad spectrum of materials and fields, including biotechnology, electronics, optics, and energy. TCFs can be produced by spin-coating substrates with silver nanowires (AgNWs) [157]. As TCFs for flexible devices, several ITO substitutes, such as carbon nanotubes, graphene, conductive polymers, and metal nanowires, have recently received much attention. AgNWs are the most promising of these materials because of their great flexibility, optoelectrical qualities, and solution-processed production [158].

#### 2.2.4. Electrodeposition

**Figure 20:**
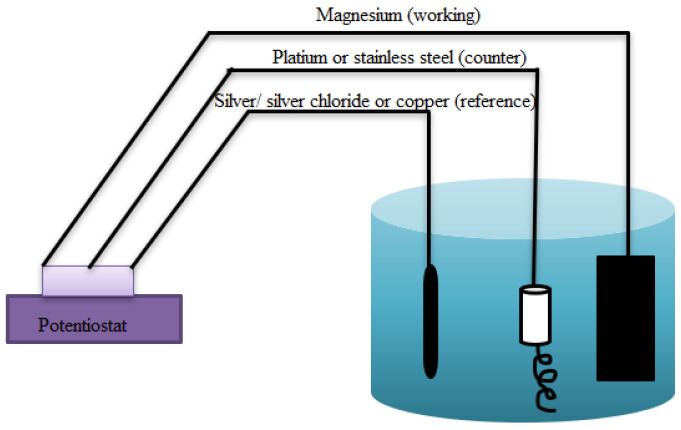
Schematic for the direct electrodeposition of conductive polymer onto a magnesium working electrode in an electrochemical cell.

The technique known as electrodeposition entails employing an electrolytic cell to deposit a small layer of material onto a substrate. Metal films are frequently deposited onto conductive or semiconductive surfaces using this technique. In this technique, the substrate or item to be coated is cleaned and may require a coating of a conductive substance. In the electrochemical cell, the substrate functions as the cathode. The metal ions that will be deposited are added to an electrolyte solution. Usually, the metal ions come from a metal salt that has been dissolved in the electrolyte [159]. The electrochemical cell receives an electric current that is applied directly. At the cathode or substrate, this current reduces the metal cations in the electrolyte, causing them to deposit as a thin layer on the substrate. A solid metal layer forms on the substrate due to the reduction that occurs when the metal cations obtain electrons at the cathode. The deposited layer’s various current densities and deposition duration can manage thickness and properties. The coated substrate may undergo further processes or post-treatment procedures, like drying and cleaning, after electrodeposition to obtain the desired qualities. Figure 20 shows the schematic diagram for this deposition process. The substrate for the conductive polymer to be placed in this configuration is the magnesium (working) electrode. The solution in the beaker, which has the polymer’s precursor ingredients, is the source. While the reference electrode (copper or silver chloride) senses potential, the potentiostat regulates the electric field generated between the working electrode (magnesium) and the counter electrode (plateau or stainless steel). This electric field drives the conductive polymer to deposit onto the magnesium substrate.

Applications for electrodeposition are numerous and include the creation of thin films, electronic components, and metal coatings for various industries. It is a popular and reasonably priced technique for producing thin metal coatings that adhere well to the substrate. It is possible to customize the selection of metal, electrolyte, and deposition conditions to fulfill particular needs for various applications. Chalcogenide materials such as cadmium sulfide are frequently employed in thin-film solar cells as window layers. Among many deposition processes available to create chalcogenide thin films, electrochemical deposition is favored for its inherent benefits, such as easy scalability and straightforward operating conditions [160]. Moreover, for investigating inorganic/organic hybrid thin film, electrodeposition is one of the intriguing potential study areas, with limitless possibilities for creating novel materials [161]. The hybrid system based on ZnO is a prime example as it has low maintenance costs, high deposition rates, stabilizes near room temperature, as well as flexibility in modifying its deposition conditions [162]; electrodeposition is also the preferred method for creating nanoengineered thermoelectric materials [163].

#### 2.2.5. Sol–Gel Deposition

**Figure 21:**
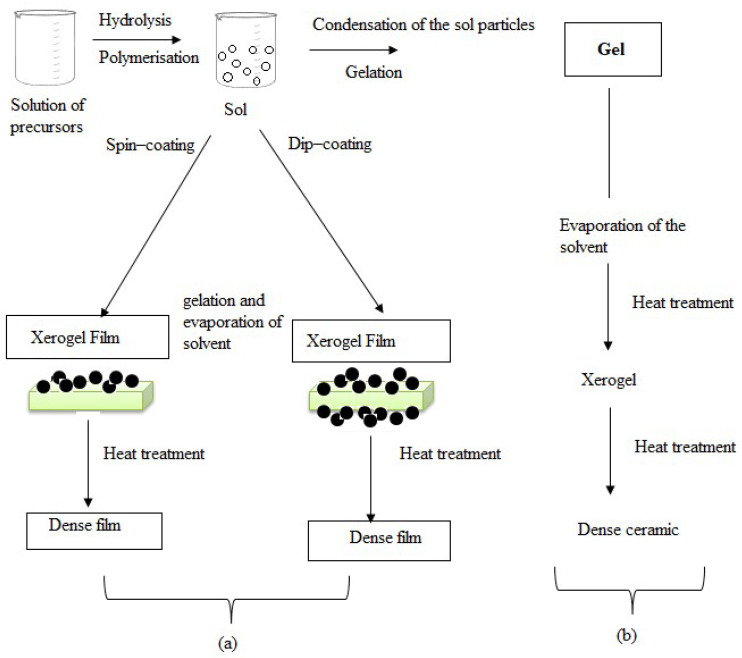
Overview showcasing two instances of sol–gel synthesis: (**a**) colloidal sol films and (**b**) colloidal sol powder that has been gelled.

The process of transforming a solution (sol) into a gel, which is subsequently deposited on a substrate and goes through additional processing to form a solid thin film, is known as the sol–gel deposition process. Creating a sol, or colloidal suspension of nanoparticles in a liquid, is the first step in the process. Usually, the sol comprises metal chlorides or alkoxides capable of condensation reactions and hydrolysis [164]. Alcohol and metal hydroxides are created when the sol’s alkoxides and water undergo hydrolysis. The metal precursor must be hydrolyzed for hydroxide groups to form. Metal oxide clusters are created due to condensation processes between the metal hydroxides. This mechanism turns the sol into a gel by generating a three-dimensional network [165]. The sol proceeds through a gelation phase, creating a network of interconnected nanoparticles in three dimensions. The viscosity of the gel is rubbery or semi-solid. The gel is applied to a substrate by spin-casting, spin-coating, or dip-coating methods. The particular needs of the thin film determine which deposition technique is best. The coated substrate is dried to expunge the solvent to solidify the gel fully into a thin layer. Heating or other methods may be used throughout the drying process. Heat treatment, known as annealing, is sometimes used to improve the thin film’s general characteristics, density, and crystallinity [164]. The end product is a thin film composed of metal oxide, or, depending on the precursor materials employed in the sol–gel process, a combination of metal oxides. An overview of the sol–gel process is shown in Figure 21. This sol–gel process creates a sol by hydrolyzing and polymerizing a precursor solution. Applying this sol to a substrate can be completed by spin-coating or dip-coating. Heat treatment is applied to eliminate solvents and densify the resulting xerogel film on the substrate, producing a dense ceramic or film. This graphic shows the steps in creating the desired film or ceramic: starting with the precursor solution, forming the sol, applying the sol to the substrate, and ultimately heating the substrate.

On a variety of substrates, protective coatings are created using sol–gel deposition. Gas sensors and biosensors employ thin films produced by sol–gel techniques [164]. Optical filters, waveguides, and anti-reflective coatings use sol–gel films [165]. Additionally, the creation of ZnO films has received much attention due to their potential uses [166]. For this reason, several sol–gel synthesis techniques have been developed [167]. Hydrophobic thin-film fabrication from a liquid phase is another popular topic with critical technological challenges. The vast range of industrial domains to which hydrophobic surfaces can be applied has led to a steady increase in interest in their manufacturing. Numerous methods can be used to deposit thin films from liquid phases on various surfaces, and the precursor solution’s composition enables the customization of the hydrophobic coating layers’ characteristics. The sol–gel process is one of the most adaptive techniques in creating hydrophobic surfaces of thin films [165]. The highly adaptable sol–gel process makes it possible to develop hydrophobic films resistant to chemicals and mechanical stress. Another most popular outcome of the sol–gel method is silica-thin films [168]. These films are used in sensors, optical devices, and coatings. Sol–gel methods yield TiO_2_ thin films, which find use in solar cells, photocatalysis, and self-cleaning coatings, among other applications [166,167,168]. Ferroelectric thin films generated from sol–gel are used in actuators, sensors, and microelectronics. Using the sol–gel process, perovskite minerals, including SrTiO_3_, can be produced as thin sheets [169].

It is clear from a thorough investigation into the sol–gel process and its uses that it is a low-temperature, sluggish process with a moderate cost. However, there are many benefits to the sol–gel technique that outweigh the drawbacks. These properties could be explained by low viscosity, which makes it easier to cover and access hard-to-reach places in complex geometries [164]. Another beneficial aspect of this process is that it allows complete control over the deposition layer’s composition [165]. The applications are likely endless since various organic and inorganic materials and their mixtures can be employed to create the appropriate deposition layer. In addition to coating, this procedure enables the production of fibers, spheres, grains, and other composite materials that may be difficult or expensive to create using sophisticated techniques like plasma sputtering [168]. However, this technique may have limitations, such as unintended fissures in the solidified portion and a sluggish pace of the process. Comparatively speaking, this approach remains a dependable and undiscovered way since it enhances the quality of components and devices in a variety of optical, sensing, and powder manufacturing applications.

#### 2.2.6. Spray Pyrolysis

In the spray pyrolysis procedure, a solution is transformed into tiny droplets and sprayed onto a heated substrate to create a precursor solution. The desired substance is left in a thin coating when the solvent evaporates. The deposited film’s qualities largely depend on the substrate temperature and the precursor solution’s chemical composition. A metal salt or a solution containing metal complexes can be this precursor. Fine droplets of the precursor solution are atomized [170]. After that, a heated substrate is sprayed with the droplets. Usually, the substrate temperature is kept at a certain level so that the precursor breaks down and forms the necessary thin film. A thin film is deposited on the substrate due to the solvent evaporating and the precursor going through chemical changes as the droplets strike the heated substrate [171]. Post-deposition treatments, including annealing, can improve the thin film’s crystallinity and other properties depending on the particular use and material.

**Figure 22:**
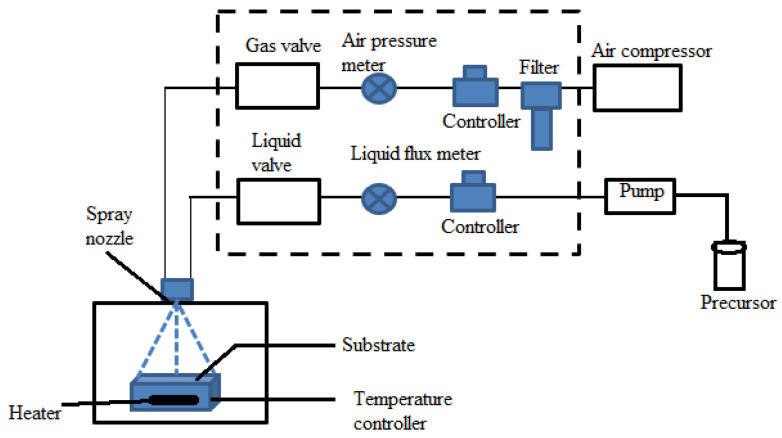
Schematic of a spray pyrolysis deposition system.

Semiconductor films, such as those formed from metal oxides like ZnO or indium oxide (INO), used in electrical devices, are deposited by spray pyrolysis [172]. Spray pyrolysis is a technique that can be used to deposit thin films for use in gas sensing applications, such as metal oxide films for gas detection. This method can deposit materials such as ITO, which is frequently used in transparent conductive coatings for electrical devices [173]. Using spray coating, silver nanowires may be effectively deposited onto substrates to form conductive films [174]. The fitting precursor solutions can create thin coatings with corrosion-resistant characteristics [170]. Figure 22 shows the schematic of a spray pyrolysis deposition system. A precursor solution is pumped out of the precursor container and atomized using a spray nozzle during the spray pyrolysis process. Droplets that have been atomized are guided onto a heated substrate within a chamber, where they break down and create a thin layer. The system has a temperature controller to keep the substrate at the proper temperature. Gas and liquid valves, flux meters, and an air compressor manage gas and fluid flow. The substrate’s heater maintains the appropriate temperature for pyrolysis.

#### 2.2.7. Anodization Methods

Anodization is a common electrochemical procedure that alters metal surfaces, mainly to improve the oxide layer’s thickness and characteristics. Anodization is essential in creating nanoporous structures for conductive thin films, which can significantly improve the material’s optical and electrical properties [175]. The process of anodization is non-vacuum. It is an electrochemical process that usually occurs at atmospheric pressure in a liquid electrolyte solution [176]. Since anodization is carried out in typical air circumstances, it is a more convenient and economical way to create oxide layers, especially for uses in creating nanoporous structures and protective coatings. Anodization techniques can be categorized according to several criteria, such as the kind of electrolyte utilized, the voltage or current used, and the particular goal or result sought (e.g., the size of the nanopores or film thickness) [177]. Table 4 summarizes the types of anodization methods of the primary categories:

As the oxide layer grows during anodization, highly ordered nanopores emerge. These nanopores can be customized in size, depth, and distribution by varying the anodization parameters, including voltage, electrolyte composition, temperature, and duration [178]. Because they provide greater surface area and light trapping, these nanoporous structures are especially helpful in TCFs, improving the transparency and conductivity of the film. Because of their special qualities, anodized nanoporous TCFs can be used in a wide range of applications, such as optoelectronic devices, sensors, and solar cells. Better electrical conductivity results from improved surface area supplied by the nanopores, which enhances the contact between the conductive layer and the underlying substrate [175]. Furthermore, these films can have their optical characteristics precisely adjusted to attain a high degree of transparency, which makes them perfect for applications requiring transparent electrodes.

**Figure 23:**
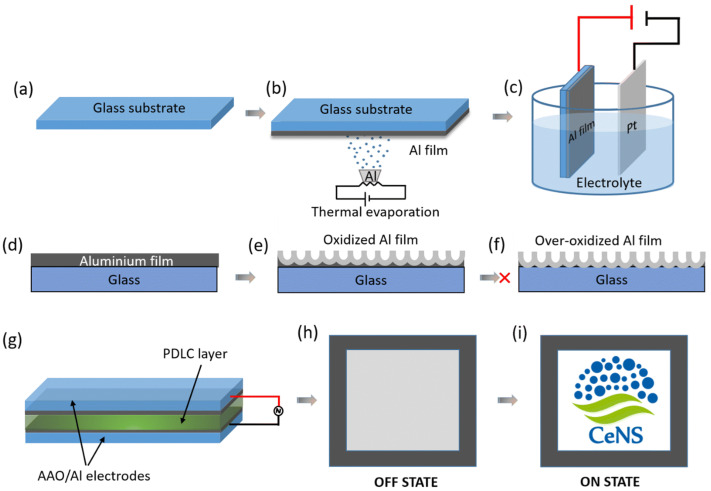
Diagrammatic representation of the following steps: (**a**–**c**) the setup for the anodization process and the deposition of the aluminum film; (**d**–**f**) Al film at various anodization stages (the cross-sectional view); and (**g**) the creation of the PDLC-based smart window device using anodized nanoporous aluminum (AAO/Al). The PDLC smart window can be in one of two states: (**h**) opaque or OFF or (**i**) transparent or ON. Reproduced from [180] under permissions from copyright clearance center.

Figure 23 illustrates the method of fabricating the PDLC smart window and transparent conducting electrode (TCE). Anodizing a thermally evaporated aluminum (Al) sheet on a glass substrate enabled its fabrication. The Al film was first thermally evaporated onto the glass. Then, platinum (Pt) foil was used as the counter electrode to anodize the glass in a 0.3 M oxalic acid electrolyte. To achieve the necessary optoelectronic properties, the thickness of the nanoporous aluminum oxide (AAO/Al) coating was optimized by meticulous management of the anodization process. Subsequently, a 20 μm thick polymer dispersed liquid crystal (PDLC) layer was sandwiched between the optimized AAO/Al TCEs to create a smart window that changes from opaque to transparent when voltage is applied. Another similar study [179] showed that Al on a glass substrate could be electrochemically anodized to create a porous transparent conductive nanomesh. The technique provides fine control over transmittance and sheet resistance by modulating the anodizing process at different current densities and modifying the applied voltage. Its consistent pore layout significantly improves optical and electrical performance, approaching that of indium tin oxide (ITO) layers.

### 2.3. Mixed Category

The mixed category of deposition techniques is a unique group of procedures used in both vacuum and non-vacuum environments [181]. These techniques are flexible enough to be adjusted to particular materials or applications by changing the pressure environment in which they are carried out. This adaptability is critical for several modern production processes where exact control over the properties of the film is required. The flexibility to transition between vacuum and air conditions in mixed-category deposition techniques enables a greater variety of material interactions and process optimizations. The pressure conditions selected can strongly impact the quality, homogeneity, and composition of the resultant thin films.

#### Metal–Organic Chemical Vapor Deposition (MOCVD)

**Figure 24:**
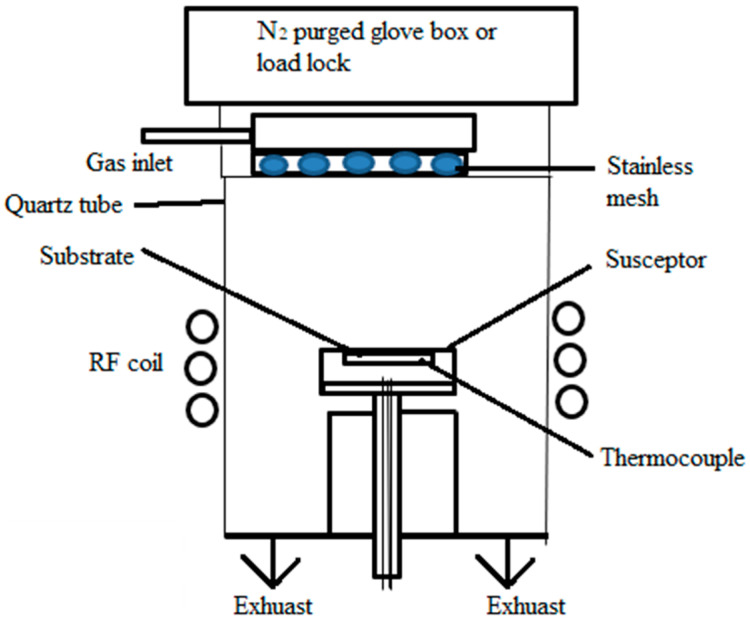
An illustration of the vertical MOCVD reactor.

MOCVD deposits thin films, particularly semiconductors, to create electrical and optoelectronic devices [130]. MOCVD employs reactive gases and volatile metal–organic precursors to produce thin coatings on a substrate, as shown in Figure 24. Precursors, either liquid or solid, are vaporized during the process. Lead alkyls were once employed as precursors; however, they are poisonous. Precursor gases enter the system through the gas inlet and pass into the quartz tube, where the reaction occurs in this process. The precursor gases injected through the gas inlet at the top of the chamber serve as the source materials. The metal–organic compounds that break down and settle on the substrate are present in these gases. The RF coil surrounding the susceptor region creates the electric field. By heating the susceptor and, subsequently, the substrate, this electric field helps to facilitate the chemical processes required for deposition. The stainless mesh aids effective gas dispersion. The exhaust eliminates byproducts, and the thermocouple monitors the temperature. A more liquid-like technique is used in modern MOCVD procedures, enabling better control over doping and homogeneity [182]. Furthermore, many layers can form simultaneously since the reaction mechanism is clear-cut and uncomplicated. Even with these benefits, MOCVD is not without its drawbacks. For instance, compared to solid precursors, liquid precursors, or alkyls, are more environmentally friendly. The resultant films are amorphous and odorless, and they pose less of a risk than lead. Additionally, MOCVD processes can be continuous, eliminating the need for replenishment throughout a deposition run. Thus, superconductor sheets can be grown using this approach [182].

In this process, metal atoms are released during the gas phase breakdown of the metal–organic precursors. The desired product is formed when these metal atoms react with other gases, such as hydrides or organics. A thin coating of the reaction products is deposited on the substrate. Usually, to speed up the deposition process, the substrate is heated to a certain temperature. As the deposition proceeds, the film builds up on the substrate layer by layer [130]. In addition to generating extremely conformal film layers, MOCVD provides excellent electrical and thermal characteristics. High-purity, high-power, and high-speed electrical devices may also be created via MOCVD. In addition, this method makes managing the film stoichiometry simpler. The main drawback of MOCVD is its difficulty applying [183]. MOCVD provides more accuracy when depositing compound and multilayered films than LPCVD, particularly for uses in sophisticated electronics and LEDs. While MOCVD is better at customizing film properties, especially for applications demanding high purity and specific material compositions, LPCVD offers higher conformality and uniformity.

MOCVD is a versatile process in the semiconductor sector because it precisely controls layer composition and thickness. Its use for synthesizing compound semiconductors, such as GaAs and InP, is crucial for producing solar cells, light-emitting diodes (LEDs), and laser diodes [184]. There are many benefits associated with MOCVD. High-quality materials may be grown using this procedure, which is also quickly developed. A MOCVD device at Northern Illinois University’s Department of Electrical Engineering can handle six reactants simultaneously [185]. Because of its adaptability, it can construct a wide range of devices using numerous materials. Transistors, solar panels, and other electronic devices can also be created via MOCVD. The primary advantage of MOCVD is its capacity to produce high-quality and easily handled materials. It is also simple and reasonably designed [186].

Table 5 is an overview of different deposition methods used to prepare various TCFs.

## 3. Requirements for Thin Films to Be Conductive

There are several requirements for a thin film to be conductive. Those are the following:

1. Selecting proper material: Either intrinsic or extrinsic conductivity is required for the thin-film material. Extrinsic conductivity results from adding dopants to a material to change its conductivity, whereas intrinsic conductivity results from the substance’s intrinsic electronic structure. Metals (like Au, Ag, and Cu), metal oxides (like ITO), and conducting polymers are frequently utilized as conductive materials in thin films.
2. Carrier density: For electrical conduction to occur, the thin film must have an adequate concentration of charge carriers, such as electrons or holes [260]. Doping or intrinsic carrier production techniques like photoexcitation or thermal activation can be used to accomplish this.
3. Charge carriers’ mobility: When an electric field is applied, the charge carriers in the thin film must be allowed to flow freely through the substance [261]. Efficient electrical conduction requires high carrier mobility, which is determined by various factors, including defects, interactions with lattice vibrations, and the crystal structure [262].
4. Film thickness: The conductivity of the thin layer may vary depending on its thickness. Thinner films can occasionally have higher conductivity because of better carrier transport qualities and less charge carrier scattering [263]. However, quantum confinement phenomena may cause overly thin films to have higher resistance.
5. Uniformity of a film and purity: The thin film’s surface should have a consistent thickness and composition to guarantee constant electrical properties. Furthermore, impurities or flaws in the film can decrease its conductivity and impede charge carrier mobility [264].
6. High electrical conductivity and low resistivity: High electrical conductivity refers to the thin layer’s ability to permit the passage of electrical current with little resistance. This is accomplished by minimizing imperfections that impede charge carrier mobility, decreasing the scattering mechanisms, and optimizing the material’s electronic band structure [265]. The value of this resistivity should be in the range of 10^−6^ to 10^−2^ Ohm cm or lower [266]. The resistivity is inversely correlated with conductivity [267].
7. Optical bandgap: Since photon energy is inversely related to wavelength, a material with a wider bandgap tends to absorb shorter wavelengths (like visible or ultraviolet light) and be transparent to longer wavelengths (like infrared). As a result, the material selection and doping level can influence the thin film’s optical characteristics and transparency at various wavelengths [268]. Table 6 overviews various conductive thin films and their required properties.

## 4. Optical Properties of Different TCFs

### 4.1. Absorption Coefficient

The degree to which a thin-film material absorbs light at a specific wavelength is indicated by its absorption coefficient. The alpha symbol, α, represents it. Understanding a material’s optical properties requires knowledge of its absorption coefficient, which is crucial in studying thin-film optics. Molecular optical absorption spectra frequently show the link between the vibrational modes of the molecule and its electronic excitation, or exciton [290].

The intensity of light (*I*) traveling through a material over a distance (*t*) is correlated with the α. The Beer–Lambert law describes the relationship as shown in Equation (5) [291]:

*I*(*t*) = *I*_0_*e*^−*αt*^ (5)

Here, *I*_0_ represents the initial intensity of light entering the material.

However, Equations (6) and (7) can be used to determine and explain the refractive indices (*n*) and α from the imaginary and real components of the complex refractive index [292],

(6) $$n=\frac{c}{\omega d}\left(\varphi_{samp}-\varphi_{ref}\right)+1$$

(7) $$\alpha =-\frac{2}{d}\ln\frac{{\left(n+1\right)}^{2}}{4n}\;\left|\frac{E_{samp}}{E_{ref}}\right|$$

where *d* is the gap of the cell, ω is the THz wave’s circular frequency, *c* is the light’s speed in vacuum, and the reference’s (which is dry air) refractive index is taken to be 1. The THz field’s phases and amplitudes as they pass through the sample and reference cell are denoted by $\varphi_{samp}$, $\varphi_{ref}$, $E_{samp}$, and $E_{ref}$, respectively. The THz-TDs system stability and the airborne water vapor absorption coefficient will cause minor inaccuracies in the testing process. Errors that occur from data processing are dominantly caused by the accuracy of the cell’s thickness [292].

Because the absorption coefficient varies depending on the wavelength of light, it is termed wavelength-dependent. It details how well a substance absorbs light at a specific wavelength. Materials with high absorption coefficients are less transparent to light at that wavelength because they show substantial absorption [290]. Designing optical coatings, solar cells, and other thin-film devices requires understanding the absorption coefficient in the context of thin films. It affects how well light is absorbed and is affected by several variables, including the thickness, composition, and wavelength of the incident light. Table 7 displays how various factors affect the absorption coefficients of conductive thin films, according to several authors. An absorption coefficient is a tool scientists and engineers use to maximize thin-film performance in various applications.

#### 4.1.1. Organic Thin Films

Organic thin films are used in many industries, including electronics, optics, and sensors. They are composed of a wide variety of materials with unique characteristics. There are many organic thin films, such as polymer thin films, OLED films, organic photovoltaic films, organic–inorganic hybrid thin films, liquid crystal thin films, and self-assembled monolayers (SAMs) [293]. Various parameters affect the absorption coefficients of these thin films.

##### Polymer Thin Films

Effect of doping concentration

Semiconducting polymers have extraordinarily high dopant concentrations (usually in parts per million levels) compared to regularly doped inorganic semiconductors. Polymer doping, in general, causes a higher carrier concentration that, as a result, generates a high conductivity [294]. This results in conjugational defects in the polymer chain, thereby generating an electron-hopping mechanism, including solitons, polarons, and bipolarons [295,296].

There have been reports on the impact of dopant concentration on polyaniline thin film doped with hydrochloric acid (PANI-HCl) film optical absorption at constant film preparation temperature [297,298,299,300], and they have found an increase in the absorption coefficient with the increasing dopant. However, another study [301] examines how dopant concentrations of 1 M and 2 M affect the optical characteristics of PANI-HCl films that are formed at various temperatures, and it was observed that, for the spectra of all the films created at various temperatures, absorption always rises with dopant concentration as the degree of polymerization increases. This also raises PANI’s surface-to-volume ratio, further improving optical absorption. The widely used polymethyl-methacrylate (PMMA) thin films were the subject of researchers, who indicated that when the doping concentration increases, the optical features of PMMA thin films alter, and the resulting composite materials may show some degree of electrical conductivity [302,303].

Undoped conjugated polymers exhibit semiconductor-like absorption bands in their absorption spectra, with bandgaps ranging from 1 to 4 eV [299]. For most conjugated polymers, the absorption process may be roughly accurately modeled as an electron excitation from the valence band to the conduction band [300]. The electron–phonon interaction causes the photogenerated carriers to self-localize quickly. The joint density of states, or absorption coefficient, near the band edge *E_c_* of an ideal one-dimensional semiconductor can be represented [300] by Equation (8).

*E_c_* ∝ (*E_photon_* − *E_v_*) ^−1/2^ (8)

Effect of molecular structure

**Figure 25:**
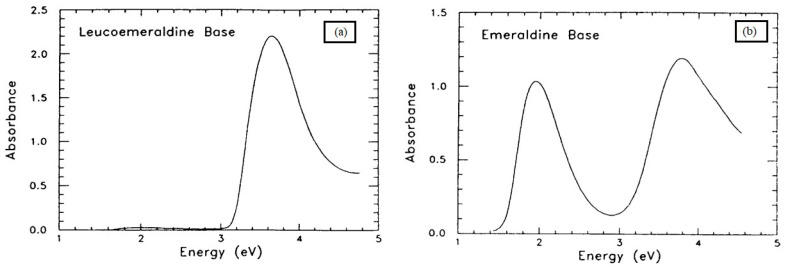
Spectra of absorption of forms of PANI: (**a**) leucoemeraldine base; (**b**) emeraldine. Reproduced from [304] under permissions from copyright clearance center.

Many aspects of the polymer’s molecular structure, including its chemical composition, chain structure, and functional groups, influence the absorption coefficient. For example, the conjugated π-electron systems of polymers, like polyacetylene or polyaniline, delocalize π-electrons along the polymer backbone, leading to higher absorption coefficients in the visible and ultraviolet areas [302]. On the other hand, polymers with aliphatic or saturated structures could have lower absorption coefficients in the visible and ultraviolet spectra because they lack conjugated π-electron systems [303]. Nevertheless, the absorption coefficient of polymers is also influenced by an array of other factors. Consequently, there have been reports of unexpected findings. For example, a study [300] observed that, even though the molecular structure and bands in polyanilines are significantly different from other conducting polymers, the absorption spectra of the (non-conducting) leucoemeraldine base form of PANI resemble the typical conjugated polymers. Conversely, an additional absorption band is observed in the (non-conducting) emeraldine base form at approximately 2.0 eV (Figure 25), located beneath the π–π* absorption band, which peaks at approximately 3.7 eV. While the absorption of emeraldine base and pernigraniline base are similar, their origins are not. However, due to the conjugational defects stated earlier, including polaron, soliton, and bipolaron states, doping causes strong new sub-gap absorption bands [300].

Effect of temperature

Many researchers have studied the influence of temperature on the absorption coefficient. According to the investigation of Stejskal et al. on polyaniline thin films [305], absorption increased noticeably as the reaction medium’s temperature decreased. This is because, as noted by [305], a drop in reaction medium temperature increases the degree of polymerization, which raises the molecular weight of the polymer and enhances absorption. According to the findings of Geethalakshmi et al.’s study [301], absorption consistently increases with dopant concentration across all films created at varying temperatures. It is also clearly observed from the study that this kind of absorption only significantly increases in films that are created at extremely low temperatures (4 °C). This indicates that the dopant’s involvement is only very effective in films that are created at low temperatures. It is well known that increasing the dopant concentration or lowering the temperature during the PANI-HCl film formation process would increase the molecular weight and thickness of the film, which will increase absorption [299,300].

Stretching effects

Polymer samples are usually somewhat amorphous and non-oriented [306]. Stretching the film is the simplest method of adding more anisotropy and order. The probing light’s polarization affects these stretched polymers’ optical absorption spectra. Compared to light polarized perpendicularly, light polarized parallel to the stretching direction is more strongly absorbed. The significant anisotropy between the intrachain and interchain transfer integrals could explain this [307]. Although a perfect order is assumed in the theoretical computations, disordered patches not orientated by stretching are nevertheless found in an actual stretch-oriented sample [300]. Because of the shorter conjugation length and the higher gap in these disordered portions, these amorphous parts contribute significantly to the absorption of the polarization perpendicular to the stretching direction and push this absorption to higher energies [308].

Effect of nanoparticles

By varying the size and proportion of gold nanoparticles, [293] research is carried out to examine the optical characteristics of gold nanocomposites, and thin-film samples were created by spin-coating a nanocomposite solution on a silicon substrate at varying speeds. A systematic fluctuation of the absorption peak width and position as a function of the percentage of gold nanoparticles in PMMA was observed in that study. This is highly beneficial as it suggests obtaining optical coatings with adjustable optical characteristics. Additionally, they discovered some quite intriguing optical absorption behavior related to interface shape. This provides us an extra parameter to adjust the composites’ optical characteristics. In another study [309], it is shown that the size of the produced nanoparticles influences the absorption of Au-PEI polymer thin films.

##### OLED Film

OLEDs generate light upon application of an electric current. The organic molecules that compose these thin films react electrically to produce light. Polymers like poly (para-phenylene vinylene) (PPV) and tiny compounds like tris(8-hydroxyquinolinato) aluminum (Alq3) are common materials used in OLEDs. The [310] study used different polymers as light-emitting layers to create red, yellow, green, and blue gravity-printed OLED devices. Their correlation was found through a comprehensive optical analysis of the polymer films that concentrated on electroluminescence (EL) and photoluminescence (PL) characteristics.

In contrast to conventional thin films, OLED thin films are non-conductive [311]. They do not have the same degree of electrical conductivity as metallic or doped semiconductor materials, even though they may carry charge carriers and permit light emission. OLED thin films emit light when an electric current flows through them, essentially serving as active layers to support the electroluminescent process. They cooperate with conductive electrodes to facilitate charge carrier flow and light emission in OLED devices [312]. Moreover, doping can improve carrier tunneling from electrodes into organic materials, removing the requirement for a second charge-injection layer and leading to the development of p-i-n OLEDs [313,314]. P-i-n OLEDs feature more balanced charge injection into the light-emitting active area than undoped OLEDs, which results in a lower operating voltage and higher efficiency. This is because of enhanced charge transport on both ends. A similar result was observed in a study incorporating delta doping into OLED [315].

The light an OLED emits is inversely correlated with its absorption coefficient. More significant absorption coefficients result in more light absorbed by the organic layers, lowering emission efficiency and light extraction. The absorption coefficient influences the color purity of light emitted. The absorption spectra of various organic materials vary, and the selection of materials influences the total absorption properties. Precise control over absorption is essential for OLED screens to provide clean and rich colors [316]. Humidity and temperature can impact how well OLED thin sheets absorb light [317]. Maintaining steady device performance requires an understanding of an effort to mitigate the impact of external factors.

Presence of “tail” states near the band edge

**Figure 26:**
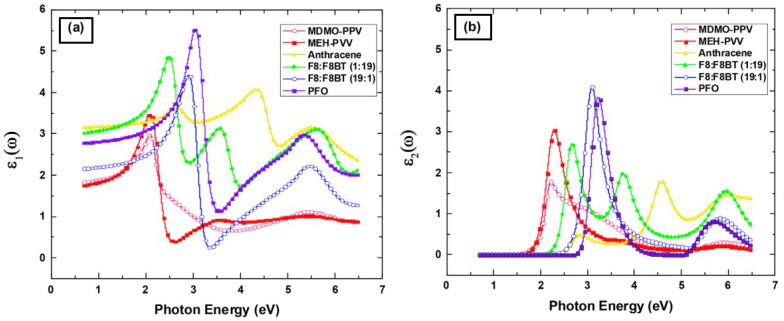
Different light-emitting polymers studied by Gioti et al. [310]: (**a**) $\;\varepsilon_{1}\left(\omega \right)$; (**b**) $\varepsilon_{2}\left(\omega \right)$. Reproduced from [310] under permissions from copyright clearance center.

In the [310], the investigated OLED films’ bulk dielectric spectra, or real part, ε_1_(ω), and imaginary part, ε_2_(ω), are shown in Figure 26a,b, respectively, as they were recreated using the best-fit parameters. Owing to the amorphous nature of the polymeric films, there are significant densities of “tai,” which are the localized states that permit a great deal of absorption but minimal transport near the band edge. Because these states are confined, they impede charge transmission but permit light absorption. This means that these materials’ energy gap is usually more significant than the value obtained from a modeling technique known as the TL (Tammann–Lippmann) dispersion equation. Tauc plots were employed by the researchers [310] to determine the energy gap with more accuracy.

Choice in anode material

In OELD, choosing anode material is essential. As it acts as the positive electrode through which electrons flow into the organic layers to start the light emission process, the anode is important to OLEDs [318]. An anode material’s work function, conductivity, and optical absorption characteristics affect its absorption coefficient. Although a high work function is essential for effective electron injection, materials with a propensity to absorb blue light, such as Au, may not be appropriate for full-color applications, even though they have a high work function. Similarly, Ti has a high work function but is not appropriate for anode reflectors due to its low conductivity and strong optical absorption. On the other hand, ozone-treated Ag thin films provide an alternative with enhanced optical transparency for specific electrical applications while having a high work function and minimal color absorption problems. However, a study [319] revealed that a top-emission OLED using Ti as an anode shows a high work function. In this instance, the anode reflector is not appropriate due to the low conductivity and strong optical absorption. Therefore, an absorption layer should be placed within an OLED or underneath a transparent OLED to reduce ambient reflection and maintain the contrast ratio in display applications.

#### 4.1.2. Inorganic Thin Films

##### AZO Thin Film

Numerous oxides that carry electricity have been found and thoroughly studied. Zinc oxide is one of the most popular for being transparent when formed into thin films. As ZnO thin films are widely used in optoelectronic devices, understanding their absorption coefficient behavior is essential. These films have special optical qualities that are necessary for solar cells, photodetectors, and LEDs, among other uses [320]. By adjusting the thickness and composition of the material, researchers can maximize device performance by having a better understanding of how light is absorbed at different wavelengths by ZnO thin films.

Effect of doping concentration and bandgap

Zinc oxide thin films show the Moss–Burstein effect, which has been reported by many researchers [218,219,220,221,223,224,321,322,323,324,325]. According to the Moss–Burstein hypothesis, the filling of electronic states close to the conduction band causes the absorption edge of strongly doped semiconductors to shift to higher energies than that of undoped semiconductors. Doping causes free carriers (holes or electrons) to be introduced, which screens the Coulombic potential and causes a bandgap renormalization. Consequently, the absorption edge experiences a blue shift due to an increase in the apparent bandgap observed in absorption spectra. There have been several studies indicating his phenomenon. In [214], it was found that using the sol–gel technique to create thin zinc oxide films doped with aluminum resulted in translucent and conductive films. The study also revealed that AZO films are direct transition-type semiconductors, indicated by the squared absorption coefficient’s linear dependency on photon energy at higher photon energies.

**Figure 27:**
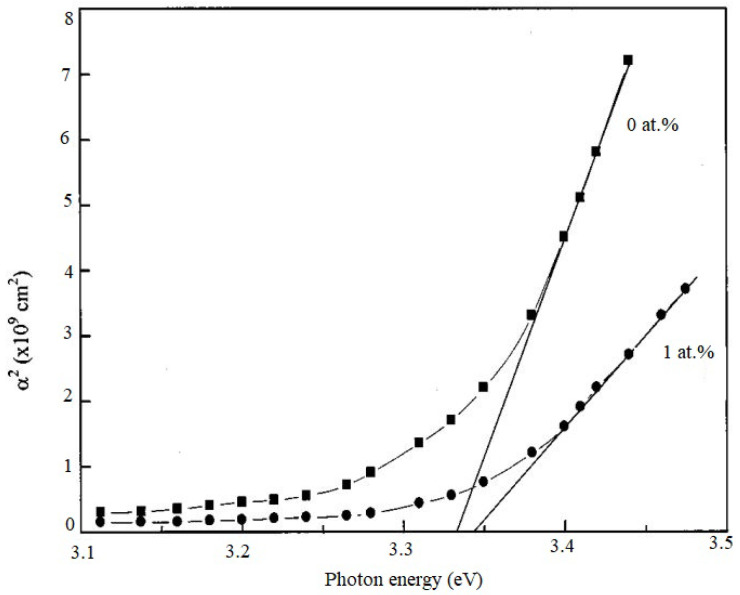
Effects on the square of the absorption coefficient for different Al dopant concentrations with changing photon energy. Reproduced from [214] under permissions from copyright clearance center.

According to Figure 27, obtained from the study mentioned in [232], a pure ZnO film’s electron gap (E_g_) is 3.333 eV, while the E_g_ of samples obtained with 1% Al doping is 3.345 eV, which shows the band expansion known as the Moss–Burstein shift. According to that hypothesis, the conduction band is occupied by the free electrons in highly doped zinc oxide films. In contrast, additional energy is needed for the valence electrons to be stimulated to higher energy states in the conduction band. As a result, doped zinc oxide films have wider optical bandgaps than undoped zinc oxide films.

According to another study [213], Al-doped ZnO thin films were prepared using the sol–gel spin-coating technique at various doping concentrations. This study revealed that the films showed minimal absorption at the visible and near-infrared energy while very high absorption at the UV range with the increasing doping concentration. From this observation, we could conclude that this thin film shows transparent characteristics in the visible and NIR regions. Additionally, they exhibit high absorbance characteristics as they have broadband gap properties.

**Figure 28:**
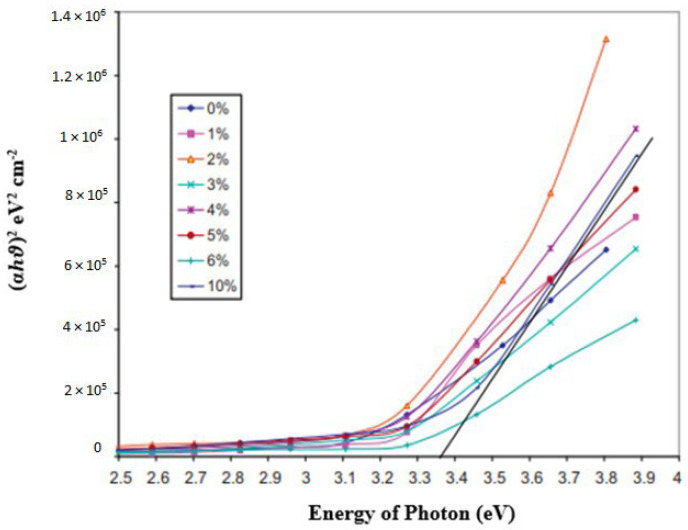
Variations in $({\alpha h\vartheta )}^{2}$ and incident photon energy for thin films created with varying aluminum doping concentrations. Reproduced from [216] under permissions from copyright clearance center.

Muiva et al. [216] calculated the E_g_ of ZnO thin films using an extrapolation method for a range of doping rates shown in Figure 28. It was found that the absorption spectrum shifts monotonically to a higher energy area with increasing Al concentration. It is observed that the absorption coefficient has the values of 3.18 eV, 3.23 eV, and 3.35 eV, respectively, for undoped, 1 at. %, and 10 at. % doped films. This supports the growing aluminum doping concentration, as reported by other workers [321]. The optical bandgap is widening, showing the Burstein–Moss effect. In a similar study [322], it is observed, that when the concentration of aluminum increases, the films’ bandgap also increases. Silva et al. [212] revealed that the band boundaries of the AZO films are blue-shifted about 330 nm, typically linked to the band-filling procedure.

A different investigation [211] was conducted on the AZO sheets with a pronounced absorption edge at a wavelength of 385 nm. The study explained that electrons in the valence band cannot be excited to move into the conduction band by light with energy below the bandgap, which is because of the bulk ZnO’s greater energy bandgap, causing it to transmit more light in the visible spectrum. Because of light scattering by compacted Al ions in the ZnO lattice, the optical transparency of AZO films has diminished with increasing Al doping. Furthermore, the UV area is where AZO films absorb most light. Because of this abrupt cut-off, AZO films are expected to be useful as UV detectors. Here, the Burstein–Moss effect is responsible for shifting the absorption edges.

Effect of free carrier concentration

In a study [217], ultrasonic spray pyrolysis (USP) was employed to deposit AZO films to explore their possible use as a top contact layer and anti-reflection coating for solar cells. For ZnO thin films, particularly semiconductors, their optical characteristics, such as the absorbance coefficient, can be impacted by the concentration of free carriers arising from deliberate doping or intrinsic flaws. A decrease in the optical transmission was observed for strongly doped materials in the photons’ free carrier absorption, indicating an increase in the absorption as transmission and absorption are inversely related. Carrier concentration and absorption are complicated by various elements, including the kind of carriers (holes or electrons), how mobile they are, and the particular mechanisms the absorption process uses. Generally, as a result of more excellent free carrier absorption, higher carrier concentrations usually result in higher optical absorption. Increased absorption of absorbed photons within the material is caused by greater carrier densities, which make more states available for electronic transitions [214].

Effect of temperature

It was reported in [216] that the transparent conducting zinc oxide (ZnO: Al) thin films were formed with float glass substrates using specially designed spray pyrolysis with an adjustment for monitoring the substrate surface temperature during deposition.

In another work [323], a cost-effective chemical solution deposition approach was used to successfully develop transparent thin films of zinc oxide doped with Al on sapphire (0 0 0 1) substrates. In that investigation, adjusting the annealing temperature of ZnO thin films did not result in any appreciable difference in the optical absorption edge. The relationship [323] mentioned in Equation (9) was used to obtain the α(ω),

(9) $$\alpha \left(\omega \right)=\left(\frac{1}{d_{f}}\right)\ln\left\{\left(1-R\right)\left[1-\frac{{\left(n_{f}-n_{s}\right)}^{2}}{{\left(n_{f}+n_{s}\right)}^{2}}\right]\times 1-\frac{{\left(n_{s}-1\right)}^{2}}{\frac{{\left(n_{s}+1\right)}^{2}}{T}}\right\}$$

where *n_f_* denotes the refractive index of the film, *n_s_* is the refractive index, and the film thickness is expressed by *d_f_*. Additionally, *R* and *T* represent the reflectance and transmittance, respectively.

Effect of the number of coatings

Research [212] was conducted to quantify the specific optical characteristics of AZO films prepared using a non-alkoxide sol–gel. This study revealed that the AZO films with varying numbers of coatings exhibited transparency in the visible spectrum with a transmission of 85% at k (λ = 600 nm). However, they reported greater absorption at shorter wavelengths (λ = 300 nm) because of band edge absorption.

##### ITO Thin Film

Thin films of TCOs have significant applications in many fields, including IR reflectors, touch panels, solar cells, panel displays, transparent electrodes for optoelectronic devices, etc. Because of its reduced electrical characteristics and increased transmittance in the visible range, ITO-thin-film has been employed nearly completely in optoelectronic devices. ITO thin films are frequently utilized as transparent, conducting electrodes on glass substrates. ITO films have been deposited onto glass substrates using various methods, including sputtering, evaporation, sol–gel, etc. ITO thin films deposited using the above methods typically require exposure to a post-annealing procedure or a somewhat high temperature to obtain acceptable electrical and optical characteristics.

Effect of thickness

ITO thin films deposited by magnetron sputtering on polymer substrates were reported in [278]. They investigated how the optical characteristics of the thin film were affected by the thickness of the film. They reported that, as the bandgap decreased, the absorption coefficient rose with thickness. This could also cause a decline in transmittance as thickness increases. Since the thinnest film has the lowest carrier concentration, fewer occupied states are in the valence band, resulting in a tiny bandgap value.

Effect of doping concentration and bandgap

With an investigation [202] on glass substrates, ITO thin films with various compositions of tin were produced by electron beam evaporation at a fixed substrate temperature in an oxygen atmosphere. The absorption coefficient’s dependence on photon energy at short wavelengths was used to calculate the optical energy bandgap (E_g_), which increased from 3.61 to 3.89 eV. This finding revealed the dopant concentration’s ascending loading profile. The Sn doping concentration was observed to alter the absorption depths of the Sn-doped In_2_O_3_ films, resulting in a drop in intensity. These peaks show the thin film’s interference. The study mentioned in [324] demonstrated the band widening, which could be described well by the Burstein–Moss shift.

However, Kim et al. [306] addressed an important issue regarding the doping concentration that excess doping can cause to reduce the absorption coefficient. As excess Sn leads to crystal instability and the Sn atoms function as carrier traps rather than electron donors, the carrier density decreases with Sn doping over the critical Sn doping. The absorption edge was moved towards lower energies due to this drop in carrier density.

Effect of wet etching

Many electro-optical devices, including flat panel displays, solar cells, and image sensors, use transparent conductive oxide materials like ITO thin sheets. Patterning the ITO film is necessary for many of these applications, and it is often completed by wet etching in acidic solutions [279]. Wet etching’s efficacy is, however, highly dependent on the characteristics of the material, including its microstructure and crystallinity, which places strict restrictions on the manufacturing process. Because of under or over-etching, it is challenging to create fine, homogenous patterns, particularly in large-area devices. ITO thin-film laser patterning performance was examined in a study [279] concerning substrate absorption. In the past, the laser wavelength was selected to absorb the ITO film, but the glass substrate was transparent to prevent over-etching and damage to the substrate. This, however, led to an incomplete removal of material at high process speeds, which caused the etched line to take on a ripple-like shape. This work showed that total material removal and the creation of residue-free etch lines can occur via intense light absorption by the substrate instead of the substrate being over-etched.

Relationship with other optical constants

According to specific research [325], the refractive index (*n*), the thickness (*d*), and the absorption coefficient of the thin film can all be determined from the interference fringes. Using the Lambert–Beer equation and the Tauc equation, the optical absorption coefficient and bandgap are found in accordance with Equations (10) and (11):

(10) $$\alpha =\frac{ln(\frac{1}{T})}{d}$$

(11) $${\left(\alpha h\vartheta \right)}^{n}=A(h\vartheta -E_{g})$$

T, d, A, h, υ, and n represent transmittance, the thickness of the material, a constant depending on the material and experimental conditions, Planck’s constant, the incident light or photon’s frequency, and a constant depending on the nature of the optical transition, respectively. The values of n are different for different scenarios. If n takes the values of 1/2, 2/3, 2, and 3, it represents direct-allowed, direct-forbidden, indirect-allowed, and indirect-forbidden transitions, respectively [326]. ITO is a direct bandgap semiconductor of the n-type, where *n* = 1/2. However, Urbach’s empirical rule, which is the relationship between the absorption coefficient and photon energy, can be used to discover more about structural characteristics. The following Equations (12) and (13) represent it,

(12) $$\alpha =\alpha_{0}exp(\frac{h\vartheta }{E_{u}})$$

(13) $$\ln\left(\alpha \right)=\frac{h\vartheta }{E_{u}}+ln(\alpha_{0})$$

Effect of carrier concentration

Absorption increases the concentration of carriers in the films and also increases light absorption. Infrared spectrum absorption, as does the tin content in ITO, tends to rise. This is because free carrier absorption, mainly caused by tin dopants, becomes more noticeable at longer wavelengths [202]. However, increased free carrier absorption brought on by higher tin dopant concentrations may cause a decrease in optical transparency, particularly in the infrared region of the spectrum.

Effect of temperature

Different studies have shown the relationship between temperature and absorption coefficients. According to [324], it is shown that with an increase in substrate temperature, the absorption coefficient increases, and the bandgap rises as well. The study further illustrated the relationship between the bandgap rise and carrier concentration rise in response to higher substrate temperatures.

However, aside from the factors discussed, variables like deposition technique and post-deposition treatments affect the absorption. It is essential to comprehend and manage these variables to balance electrical conductivity and optical transparency while optimizing ITO thin films for particular applications. ITO may still absorb some light, even though it is essentially transparent in the visible spectrum. ITO’s thickness and deposition conditions should be chosen with the needs of the device or application in mind.

##### CuI Thin Film

Effect of temperature

**Figure 29:**
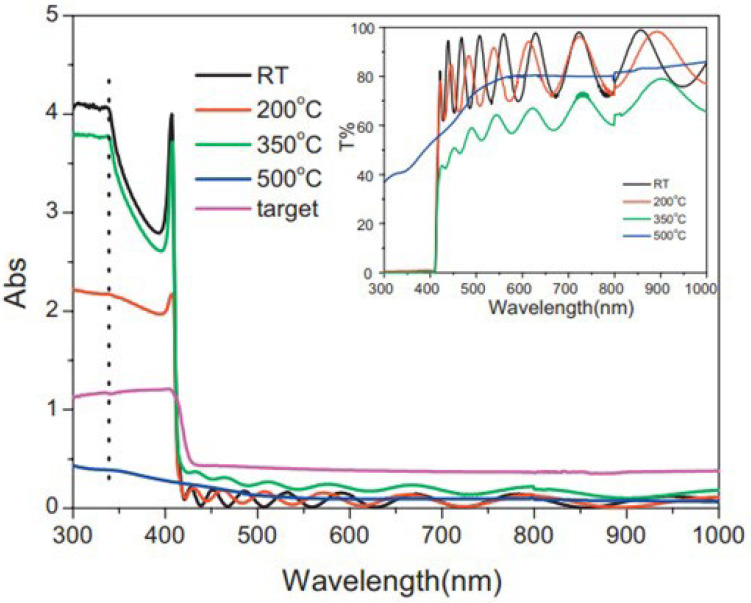
Absorption spectra of CuI at different substrate temperatures. Reproduced from [190] under permissions from copyright clearance center.

In general, the CuI-thin-film absorption coefficient tends to decrease with increasing temperature. There are multiple reasons for this behavior. In semiconductor materials like CuI, temperature-induced lattice vibrations, or phonons, can cause the energy bandgap to widen [327]. Consequently, the material absorbs less photons, which lowers the absorption coefficient. Furthermore, thermal excitation of charge carriers can happen at high temperatures, which results in greater mobility and shorter carrier lifetimes. A weakening of absorption properties linked to localized states inside the bandgap may arise from this phenomenon [328]. Notably, the absorption coefficient’s particular temperature dependency in CuI thin films can change based on several variables, including dopant concentration, crystalline structure, and film thickness. As the temperature increased, the absorption spectra decreased, as reported by Zhu and Zhao [190], as shown in Figure 29.

Effect of doping concentration

The doping concentration affects the optical absorption of CuI thin films. Without causing structural alterations, Wang et al. [329] reported increased optical absorption in CuI-thin-film photovoltaic cells doped with silver. According to the study by Naveena et al. [330], the La-doped CuI films show strong optical absorption in UV spectra, and their bandgap value drops from 1.62 eV to 1.49 eV up to 6 wt. % of doping. However, with an intrinsic absorption value of α < 200 cm^−1^ at wavelengths exceeding 450 nm, the produced CuI thin films exhibit remarkable transparency in the visible and near-infrared (NIR) spectrum [188].

Effect of thickness

A study conducted in [331] displays the absorbance coefficients of CuI films under various deposition conditions. Examining these data closely shows that the thick films deposited at a higher flow rate have a lower absorption coefficient than the thin films in the high-energy zone. Larger crystallites or grain sizes that can be obtained for thicker, thin films may be the cause of this phenomenon. Because of this, thicker thin films are more similar to bulk crystalline CuI; nevertheless, greater grain sizes cause more empty intergranular volume, which lowers absorption per unit thickness [332].

##### Cadmium Oxide (CdO) Thin Film

Effect of temperature

Ullah et al. [333] investigated the absorption coefficient and found that, when annealing was conducted at different temperatures, the absorption coefficient decreased. According to another study [334], the absorption edge changes to higher wavelengths for the film annealed at higher temperatures.

Effect of doping and bandgap

**Figure 30:**
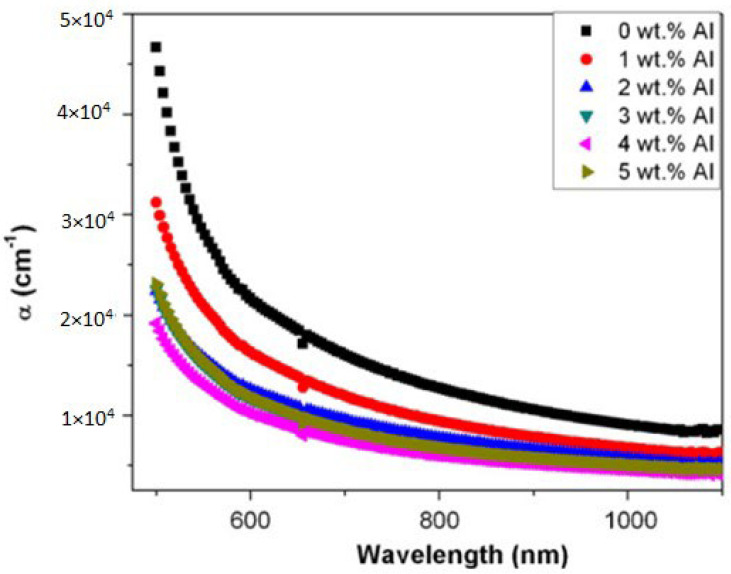
Absorption coefficient vs wavelength. Reproduced from [335] under permissions from copyright clearance center.

Kumaravel et al. [335] investigated the effects of Al doping on CdO thin films. Figure 30 illustrates how absorbance drops as the concentration of Al-doping rises, with a particularly steep drop observed close to the band edge.

The undoped CdO film’s bandgap value is 2.36 eV [335]. Up to 3 weight percent (2.53 eV), the bandgap value rises with increasing Al doping concentration; at greater doping levels, it falls. An increase in the films’ concentration of free electrons causes the bandgap to expand. The Burstein–Moss shift helps explain how the bandgap shifts as the carrier concentration changes [336,337]. Usharani et al. [338] showed that CdO film doped with 2 wt% Mg shows a blue shift as the bandgap widens with increasing Mg concentration. The increased bandgap measurements with Mg doping indicate consistent replacement of Cd^2+^ ions in the lattice by Mg^2+^ ions. It is also suggested that quantum confinement is a contributing factor in the bandgap changes in doped materials.

The bandgap is found to grow with Al concentration up to 1% to CdO thin film in the study [339] of the effects of Al doping on bandgap values, and then it starts to decrease with greater Al concentrations. The Burstein–Moss effect, which links changes in carrier concentration to changes in the bandgap, is responsible for this behavior. The direct bandgap experiences a blue shift as a result of the BM effect’s elevated carrier concentration. It is pointed out that the BM effect does not entirely explain the decrease in carrier concentration brought on by grain boundary barriers. Grain boundaries and electronic configuration both affect the subsequent decrease in carrier concentration, pointing to partial applicability of the BM effect in explaining the observed bandgap changes.

In a similar study [340], a trend of increasing bandgap with increasing La dopant concentration up to 0.75 weight percent is observed in the analysis of La dopant impacts on bandgap values. Nevertheless, the bandgap starts to decrease after this threshold. This phenomenon, characterized by a blue shift in the absorption edge, is another example of the frequently referred Moss–Burstein’s band widening effect. An increase in carrier concentration brought on by doping is responsible for this blue shift. This observation emphasizes the complex relationship between dopant concentration and bandgap behavior, as well as the impact of dopant concentration on the electrical characteristics of the material.

**Table 7:** An overview of the impact of major factors on the absorption coefficients of TCFs.

TCFs	Factors	Effects on the Absorption Coefficients	Authors
AZO	Doping	Shows the Moss–Burstein effect Doped ZnO has wider optical bandgaps than undoped zinc oxide films The free electrons occupy the conduction band in highly doped zinc oxide films.	Alam et al. [214], Mamat et al. [213], Muiva et al. [216], Mia et al. [211], Silva et al. [212], Manjakkal et al. [322], Majumder et al. [323]
		AZO has very high absorption at the UV range Shows transparent characteristics in the visible and NIR regions	Mamat et al. [213]
		The absorption spectrum shifts monotonically to a higher energy	Muiva et al. [216]
		AZO films are expected to be useful as UV detector	Mia et al. [211]
		Al doping produces donor levels below the conducting band, bending the band edge and reducing the bandgap.	Kaur et al. [230]
		α in UV region > Visible region α decreased with Al doping	Caglar et al. [215]
		Burstein–Moss shift Band widening	Tuna et al. [324]
	Free Carrier Concertation	An increase in the absorption with increasing carrier	Alam et al. [214], Babu et al. [217]
		Bandgap widening is proportional to N^2/3^	Mosbah et al. [221]
	Temperature	Annealing temperature did not result in any appreciable difference.	Majumder et al. [323]
		Bandgap increases with increasing substrate temperature.	
		α decreases with increasing temperature	Barhoumi et al. [219]
	Thickness	The bandgap decreases with the increasing film thickness	Kaur et al. [230]
ITO	Doping	Burstein–Moss shift Band widening	Tuna et al. [324]
		Excess doping can cause to reduce the absorption coefficient Excess Sn leads to crystal instability, and the Sn atoms function as carrier traps rather than electron donors	Kim et al. [273]
	Free carrier concentration	Since the thinnest film has the lowest carrier concentration, fewer occupied states are in the valence band, resulting in a tiny bandgap value	Eshaghi and Graeli [278]
		The carrier density decreases with Sn doping over the critical Sn doping	Kim et al. [273]
		Absorption increases the concentration of carriers in the films and increases light absorption. Free carrier absorption, mainly caused by tin dopants, becomes more noticeable at longer wavelengths Increased free carrier absorption brought on by higher tin dopant concentrations may cause a decrease in optical transparency	Senthilkumar et al. [202]
	Thickness	The bandgap decreased, and the absorption coefficient rose with thickness.	Eshaghi and Graeli [278]
		A strong absorption edge is a well-known property of pure semiconducting substances. The absorption edge became sharper and shifted to longer wavelengths as the film thickness increased.	Ahmed et al. [341]
	Wet Etching	Intense light absorption by the substrate can achieve entire material removal and the formation of residue-free etch lines without over-etching the substrate.	Yavas and Takai [279]
	Temperature	With an increase in substrate temperature, the absorption coefficient increases, and the bandgap rises as well.	Tuna et al. [324]
		The absorbance coefficient decreases as the annealing temperature increases.	Mohamed [270]
CuI	Doping	Optical absorption increased in CuI-thin-film with doping silver concentration.	Wang et al. [329]
		La-doped CuI films show strong optical absorption in UV spectra, and their bandgap value drops for up to 6 wt.% of doping.	Naveena et al. [330]
		The produced CuI thin films exhibit remarkable transparency in the visible and near-infrared (NIR) spectra.	Yang et al. [188]
	Thickness	Thick films deposited at a higher flow rate have a lower absorption coefficient than the thin films in the high-energy zone Larger crystallites or grain sizes obtained for thicker films	Amalina et al. [331]
		Greater grain sizes cause more empty intergranular volume, which lowers absorption per unit thickness.	Abbas et al. [332]
	Temperature	Temperature-induced lattice vibrations, or phonons, can cause the energy bandgap to widen The material absorbs less photons, which lowers the absorption coefficient	Villegas et al. [327]
		Thermal excitation of charge carriers can happen at high temperatures, which results in greater mobility and shorter carrier lifetimes. A weakening of absorption properties linked to localized states inside the bandgap may arise due to high temperature	John and Wang [328]
		As the temperature increased, the absorption spectra decreased.	Zhu and Zhao [175]
CdO	Doping	Absorbance drops as the concentration of Al is doped in CdO thin films	Kumaravel et al. [335]
		The bandgap value rises with increasing Al doping concentration; at greater doping levels, it falls An increase in the films’ concentration of free electrons causes the bandgap to expand. Shows Burstein–Moss shift Dopant elements change the electron concentration in the conduction band, which modifies the bandgap energy and Fermi level.	Moss et al. [336], Burstein et al. [337]
		CdO film doped with 2 at. % Mg shows a blue shift as the bandgap widens with increasing Mg concentration. Mg doping indicates consistent replacement of Cd^2+^ ions in the lattice by Mg^2+^ ions. Quantum confinement is a contributing factor in the bandgap changes in doped materials.	Usharani et al. [338]
		The bandgap is found to grow with Al concentrations up to 1%, and then it starts to decrease with greater Al concentrations. The changes in carrier concentration and changes in the bandgap are responsible for this behavior. The direct bandgap experiences a blue shift as a result of the BM effect’s elevated carrier concentration. The BM effect does not entirely explain the decrease in carrier concentration brought on by grain boundary barriers. Grain boundaries and electronic configuration both affect the subsequent decrease in carrier concentration, pointing to partial applicability of the BM effect in explaining the observed bandgap changes.	Abdolahzadeh Ziabari et al. [339]
		The bandgap increases with La dopant concentration up to 0.75 weight percent; the bandgap starts to decrease after this threshold. An increase in carrier concentration brought on by doping is responsible for the blue shift.	Velusamy et al. [340]
	Temperature	When annealing was conducted at higher temperatures, the absorption edge decreased.	Ullah et al. [333]
		The absorption edge changes to higher wavelengths for the film annealed at higher temperatures.	Santos-Cruz et al. [334]
Polymer thin film	Doping	Causes a higher carrier concentration Generates a high conductivity. Creates conjugational defects in the polymer chain, thereby generating an electron-hopping mechanism, including solitons, polarons, and bipolarons	Yoon et al. [294], Geethalakshmi et al. [301]
		An increase in the absorption coefficient with the increasing dopant on PANI-HCl	Banerjee et al. [295], Ayad et al. [296]
		Absorption always rises with dopant concentration as the degree of polymerization increases Raises PANI’s surface-to-volume ratio and improves optical absorption	Geethalakshmi et al. [301]
		When the doping concentration increases, the optical features of PMMA thin films alter, and the resulting composite materials may show some degree of electrical conductivity.	Chan and Kammer [297], Ou et al. [298]
	Molecular structure	Polymers like polyacetylene or polyaniline have conjugated π-electron systems that delocalize π-electrons along the polymer backbone, leading to higher α in the visible and ultraviolet areas.	Kar et al. [302]
		Polymers with aliphatic or saturated structures could have lower absorption coefficients in the visible and ultraviolet spectra because they lack conjugated π-electron systems.	Kiebooms et al. [303]
		While polyanilines have different bands and molecular structures from other conducting polymers, their absorption spectra are comparable to those of other conjugated polymers.	Stubb et al. [300]
	Temperature	Absorption increased noticeably for polyaniline thin films as the reaction medium’s temperature decreased. A drop in reaction medium temperature increases the degree of polymerization, which raises the molecular weight of the polymer and enhances absorption.	Stejskal et al. [305]
		The dopant’s involvement is only very effective in films that are created at low temperatures. Increasing the dopant concentration or lowering the temperature during the PANI-HCl film formation process would increase the molecular weight and thickness of the film, which will increase absorption	Geethalakshmi et al. [301], Banerjee et al. [295], Ayad et al. [296].
INO	Temperature	Films deposited at varying substrate temperatures have various bandgap values; at higher temperatures, the bandgap energies are higher, and the particle sizes are smaller. Increased bandgap values for films deposited at higher temperatures, possibly due to grain boundary band bending.	Beena et al. [342]
		Due to the Burstein–Moss shift, the bandgap increases with temperature increasing.	Prathap et al. [343], Gupta et al. [344], Senthilkumar and Vickraman [345]
	Particle size	Quantum confinement theory states that, as particle size decreases, bandgap energy rises. Smaller crystallites show more noticeable band-bending effects, which leads to reduced bandgap energies compared to larger crystallites	Beena et al. [342]

### 4.2. Transmittance

The amount of incident light that travels through a thin layer without being absorbed or reflected is known as optical transmittance. It is an indicator of how well the film transmits light. Optical transmittance is supplementary to optical absorbance and is commonly stated as a percentage. Regarding thin films, several factors affect how much light may pass through the film, such as film thickness, doping concentration, surface morphology, etc. Interference effects are frequently observed in thin films depending on the film’s thickness. The total transmittance can be impacted by interference, which amplifies or inhibits specific wavelengths.

Two important factors are the material’s refractive index and absorption qualities, which are utilized in the thin film. The transmittance can change depending on the incident light’s wavelength. This is especially crucial when working with films that have significant wavelength interactions. Equation (14) describes the relationship between transmittance, absorbance (*A*), and reflectance:

*T* + *R* + *A* = 1 (14)

The absorption coefficient can be computed using the expression based on the transmission spectra in the ultraviolet area, as mentioned in Equation (15),

(15) $$I=I_{0}e^{-\alpha t}$$

where *t* is the film’s thickness, *I*_0_ is the incident light’s intensity, and *I* is the transmitted light’s intensity.

Using Swanepoel’s envelope approach and the optical transmittance interference data, the refractive index of the films was calculated from the relations mentioned in Equations (16) and (17),

(16) $$n\left(\lambda \right)={\left[N+{\left(N^{2}-n_{0}^{2}n_{1}^{2}\right)}^{\frac{1}{2}}\right]}^{1/2}$$

(17) $$N=2n_{0}n_{1}\left[\frac{T_{M}-T_{m}}{T_{M}T_{m}}\right]+[\frac{\left(n_{0}^{2}+n_{1}^{2}\right)}{2}]$$

Comprehending thin-film optical transmittance is essential in various applications, including photovoltaics, displays, coatings, and optics. Measurement and analysis of thin-film transmittance qualities are frequently conducted via experimental methods such as spectrophotometry and ellipsometry.

#### 4.2.1. AZO Thin Film

In AZO thin films, transmittance is a crucial optical characteristic since it tells us a great deal about how much light can pass through the substance. The visible spectrum’s high transmittance is vital for applications where transparency is required because it permits visible light to pass through while retaining electrical conductivity. ZnO is frequently employed as a TCO layer in solar cells. In order to maximize the amount of sunlight that reaches the solar cell’s active layers, high transmittance in the visible range is preferred.

##### Effect of Film Thickness

The film thickness greatly affects the characteristics of the thin film. ZnO films that are thinner frequently have greater optical transmittance, particularly in the visible light spectrum. Transmittance may decrease due to increased light absorption in the film caused by thickness, as shown by many researchers [230,345]. This characteristic is essential for uses such as transparent conductive coatings on solar cells and displays.

According to Hoon et al.’s [346] analysis of the impact of film thickness revealed that, at wavelengths between 400 and 850 nm, the transmittance dropped to 50–70% from 70–90% when the deposited film thickness exceeded 700 nm. Using RF magnetron sputtering techniques, ZnO films in the 160–398 nm thickness range were investigated in [223]. Film thickness’s impact on ZnO films’ structural, morphological, and optical characteristics was thoroughly investigated. It was discovered that the optical transmittance of ZnO films rose as the film thickness increased to 231 nm and dropped at a higher thickness of 398 nm. In the visible wavelength region of 450–650 nm, the average optical transmittance was 89%.

##### Effect of Temperature

ZnO films are frequently annealed at high temperatures to increase crystallinity and decrease flaws [347]. Annealing may result in improved transmittance, which can enhance the optical characteristics. Notably, the precise influence of temperature on ZnO-thin-film transmittance is contingent upon various parameters, including the film’s initial qualities, the deposition conditions, and the intended use.

**Figure 31:**
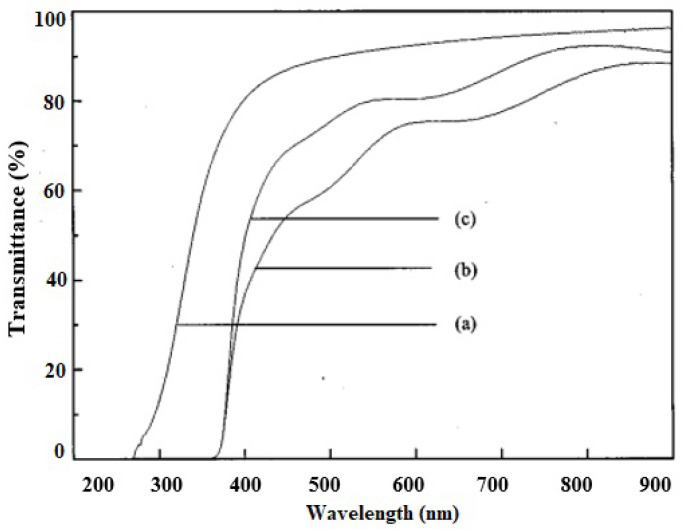
Optical transmittance of glass and AZO films placed on glass at (a) uncoated, (b) 400 °C, and (c) 500 °C with different annealing temperatures. Reproduced from [214] under permissions from copyright clearance center.

According to the study [214], the thin film’s transmittance was determined using a UV–vis spectrometer. Figure 31 displays the optical transmittance of ZnO: Al films (0.8 at. % Al) deposited on glass with a thickness of 300 nm. At 900 nm, the films’ optical transparency reaches 91%. The thickness of the film and the ensuing interference in the layer are the causes of the oscillations on the spectrum. ZnO: Al films exhibit enhanced optical transmission in the visible spectrum upon annealing at elevated temperatures. At 900 nm wavelength, the maximum transmittance is 88% and 91% for films annealed at 400 °C and 500 °C temperature, respectively. According to Gao et al. [348], quick thermal annealing and the pulsed-filtered cathodic arc method were used to create ZnO: Al films on glass substrates with good visual and infrared transmission characteristics. They demonstrated that the visible transmittance (∼85%) stays almost constant as the annealing temperature rises from 500 to 650 °C, while the infrared transmittance (from 780 to 2500 nm) significantly improves from 22% for the as-deposited film to 58% at 600 °C and 71% at 650 °C for the annealed films. The films’ transmittance and crystallinity were enhanced by the high-temperature annealing [349].

The substrate temperature is critical during film deposition. Elevated substrate temperatures have the potential to enhance film adherence and impact the formation of crystalline formations, which in turn affects the optical qualities. During film growth or annealing, a significant temperature differential between the substrate and the surrounding air could introduce thermal stress. Thermal stress can affect the structural integrity of the film and, in turn, its optical performance. A different investigation [217] also found a similar result on the substrate’s temperature affecting the films’ transmittance.

According to the study by Barhoumi et al. [219], as substrate temperature increased, the average optical transmittance changed from 78% to 90%. The average grain size of the film may have increased, which could be the cause of the transmission increase. The increased homogeneity and crystallinity of AZO thin films can be credited with improving transmittance.

##### Effect of Doping Concentrations

**Figure 32:**
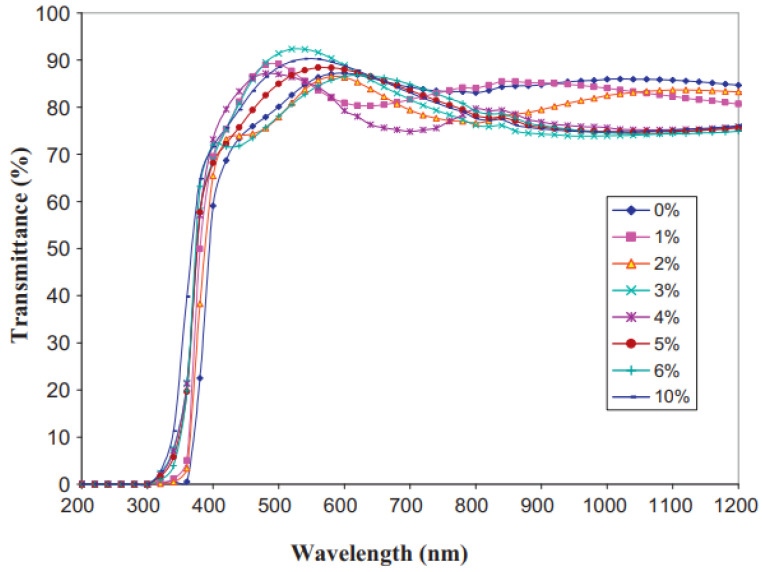
The transmittance spectra of films created at various initial aluminum atomic ratios. Reduced [216] under permissions from copyright clearance center.

It is important to note that the precise parameters of the doping procedure and the intended application will determine the real influence on transmittance. Scholars frequently carry out comprehensive optical and electrical characterizations to comprehend how doping affects ZnO thin-film performance. Post-deposition treatments and the choice in deposition technique can significantly influence the ultimate characteristics of doped ZnO films. Generally, with increasing doping concentrations, the transmittance usually decreases [221,224,350]. Typically, films grown under ideal deposition conditions exhibit high transmittance. The electrical, optical, and microstructural characteristics of the sprayed ZnO at high Al doping concentration were investigated in [217]. Higher doping levels (>2 at. %) may cause more photons to be scattered by doped crystal defects, which would explain the drop in transmittance [350]. Another possible explanation for the observed decrease in the optical transmission of strongly doped materials is the photons’ free carrier absorption. Additionally, the optical transmission spectra of zinc oxide films grown on glass substrates at 420 °C substrate temperature with varying amounts of aluminum doping is displayed in Figure 32 from a similar study [216]. Regardless of doping concentration, all films have high transparency in the visible (400–750 nm) optical spectrum, with values as high as 85% at 550 nm. It is important to note that the resulting layers will provide an adequate visible optical window for optical applications. Another similar study [215] showed the films appear to have a notably high transmission rate with Al doping.

According to [217], all films with different doping concentrations had optical transmittances ranging from 75 to 90% in the visible wavelength range of 600–700 nm. At 450 °C, the doped film containing 2 at. % Al has a transmittance over 90% higher than the doped film containing 3 and 4 at. %. This could be because there are more voids in the film with 2 at % than in the films with 3 and 4 at % doping. This could result in an increase in transmittance and a decrease in optical scattering.

It is commonly known that bulk ZnO’s higher energy bandgap causes its transmittance to be higher in the visible spectrum. Another study revealed [211] that light scattering by compressed Al ions in the ZnO lattice causes a lowering of the optical transparency of AZO films. The AZO thin films achieved the maximum transmittance of 97%, which decreased with increased Al concentration.

##### Effect of Surface Morphology

It is well known that the optical transmittance of films depends on the surface morphology. With the increased amount of Al, the films often have a whitish color, a powdery texture, and polycrystalline morphology, as shown in the study [217] with its FESEM analyses. The enhanced photon scattering caused by doping-induced crystal defects may cause the transmittance to drop at higher doping levels. Another possible explanation for the observed decrease in the optical transmission of strongly doped materials is the photons’ free carrier absorption. Additionally, ZnO-thin-film surface morphology can be influenced by film thickness. Grain and roughness patterns on the surface may be more noticeable in thicker coatings. This may affect how well the film works in optical and electrical applications.

##### Effect of Coating

The transmittance is independent of the type or process of coating. A study mentioned in [212] provided evidence of this claim and revealed that the quantity of coatings had no appreciable impact on transmittances or bandgaps.

#### 4.2.2. ITO Thin Film

##### Effect of Film Thickness

In general, with the increasing thin-film thickness, the transmittance decreases. A prior study used magnetron sputtering to deposit transparent conducting ITO thin films on polymer substrates [278]. The investigation focused on how the thickness of the thin film affected the optical characteristics of ITO thin films. It is evident that, as ITO thin film-thickness increases, its transmittance decreases. The influence of grain size may be connected to this phenomenon. Light scattering results from an increase in grain size brought on by thin-film thickness. Furthermore, there is a modest drop in average transmittance as thickness increases. This phenomenon could be attributed to free carrier absorption, which raises the concentration of carriers in thick films and enhances light absorption. It is also revealed that, as the absorption coefficient rose, and the bandgap shrank with increasing thickness. This may provide an additional justification for transmittance decrees when thickness increases.

Optical transmission measurements were obtained from the study of the ITO thin films [351]. The measurements reveal strong transmittance throughout the visible spectrum with decreasing thickness, with a sharp decline at 380 nm due to photons with energies over the bandgap being absorbed. Approximately 1000 nm is the wavelength at which the transmittance decreases because of the high carrier concentration at higher wavelengths.

##### Effect on Changing Compositions

**Figure 33:**
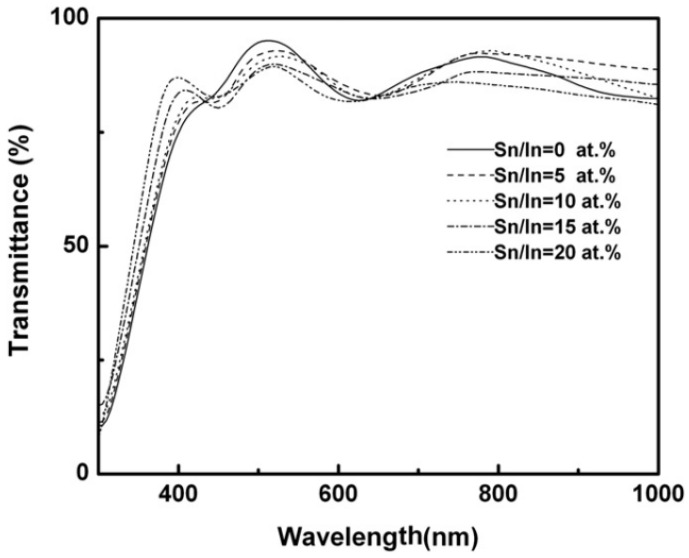
ITO film optical transmittance spectra with varying Sn doping concentrations. Reproduced from [202] under permissions from copyright clearance center.

Numerous studies have been carried out to examine the impact of compositional changes in ITO thin films. Most studies revealed that, when Sn doping concentration rises, optical tests indicate a modest decrease in transmittance. A study [222] used electron beam evaporation to analyze ITO thin films in various compositions on glass substrates. Figure 33 displays the optical transmission spectra of ITO films captured in the 300–1000 nm wavelength range during that work. In the visible spectrum, the films’ transmittance showed a noticeable rise. The ripple pattern in Figure 33 results from the interference of light with nanostructured materials [98]. It was discovered that the transmission peaked for undoped In_2_O_3_ declined as the quantity of Sn increased. The reduction in light transmission was linked to (i) scattering at grain boundaries and (ii) oxygen vacancies. Since every film was deposited in an oxygen-rich environment and under identical processing circumstances, it was anticipated to experience the same transmission loss from oxygen flaws.

##### Effect of Grain Size

Senthilkumar et al. [202] showed that with the increasing concentration of Sn, the grain size decreased, resulting in a decrease in the transmittance. The reduction in transmission could be attributed to increased scattering centers brought on by more grain boundaries and increased Sn dopant content. Using the Scherrer equation [352], the grain size of the ITO film was determined from the line broadening of the (222) diffraction line, and the result was shown as a graphical representation in the study,

(18) D=0.9λ/βcosθ

**Figure 34:**
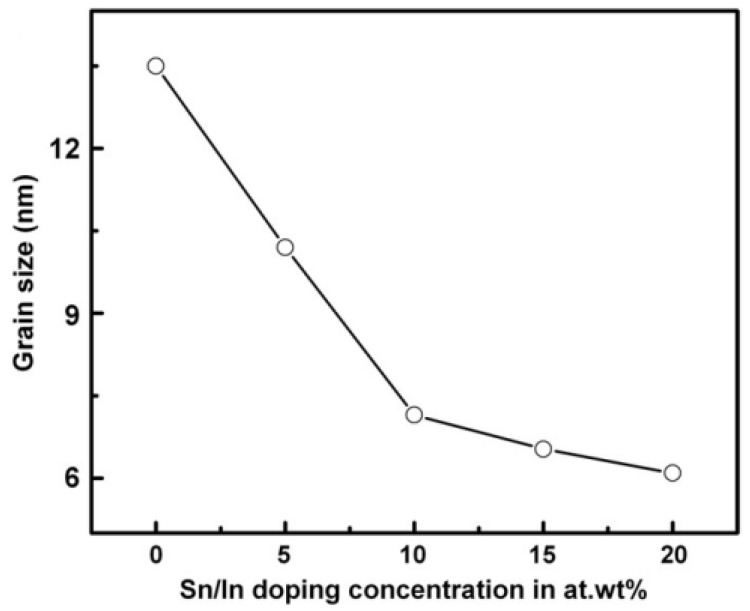
Grain size fluctuation with varying amounts of Sn doping. Reproduced from [202] under permissions from copyright clearance center.

Here, X-ray radiation’s wavelength and diffraction angle are represented by *λ* and β, respectively. Moreover, the study [202] also revealed that the grain boundaries in In_2_O_3_ films increased due to a decrease in grain size and increased Sn content. The full width at half maximum (FWHM) increased, but the peak intensity fell as the tin level increased, as shown in Figure 34. Both Ogihara et al. [353] and Katsube et al. [354] reported the same trend of observation for ITO films created by vacuum evaporation [355] and the thermal breakdown of indium nitrile acetylacetonate.

##### Effect of Surface Morphology

ITO films have the potential to be shortened with layers above them as their roughness is crucial in various applications, including thin-film photovoltaics and displays [353]. The surface shape also influences the impact of transmittance. Senthilkumar et al. [202] observed the surface morphological characteristics of doped ITO films with Sn/In = 0 and Sn/In = 20 at. wt.%. The morphology of the films was shown to be influenced by the integration of Sn in various areas of wt.% samples. All the films were found to be smooth and to have distinct nanocrystallites. Tin doping caused a notable shift in the crystallite’s size. The study also revealed that porosity rose when grain size decreased, as observed from the morphology. It was also found that, as the Sn concentration increased, transmittance also decreased.

##### Effect of Deposition Power and Time

**Figure 35:**
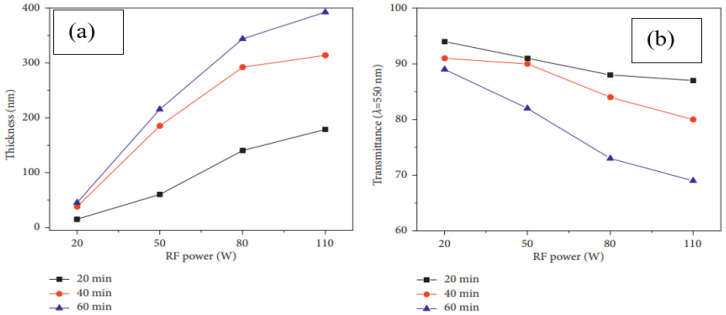
Shows the evolution of the ITO films’ (**a**) thickness and (**b**) transmittance at varied powers and deposition times. Reproduced from [325] under permissions from copyright clearance center.

**Figure 36:**
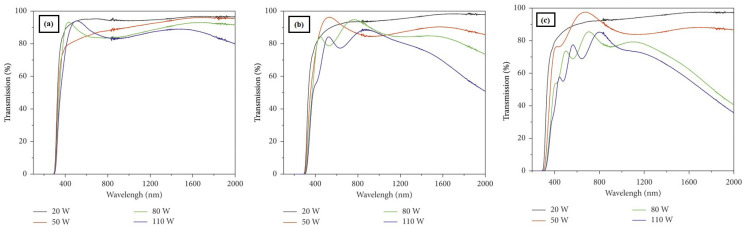
ITO films’ transmittance after being deposited at varied powers and deposition durations: (**a**) 20 min; (**b**) 40 min; (**c**) 60 min. Reproduced from [325] under permissions from copyright clearance center.

With the increment of the deposition time or power, a decrease in the average transmission is observed. A study [325] reports the optical transmittance spectra of ITO films deposited in the 200–2000 nm wavelength region with varying powers and deposition times, which are demonstrated in Figure 35 and Figure 36. For deposition power larger than 20 W, interference fringes are observed in the transmittance spectrum. ITO film transmittance falls when deposition power and duration increase. As shown in Figure 35a, it is caused by the film thickness’s increment with an increase in deposition time and RF power. Figure 35b illustrates that the average transmittance fluctuates between 70% and 95% and decreases with increasing RF strength and deposition time.

#### 4.2.3. CuI Thin Film

##### Effect of Deposition Technique

**Figure 37:**
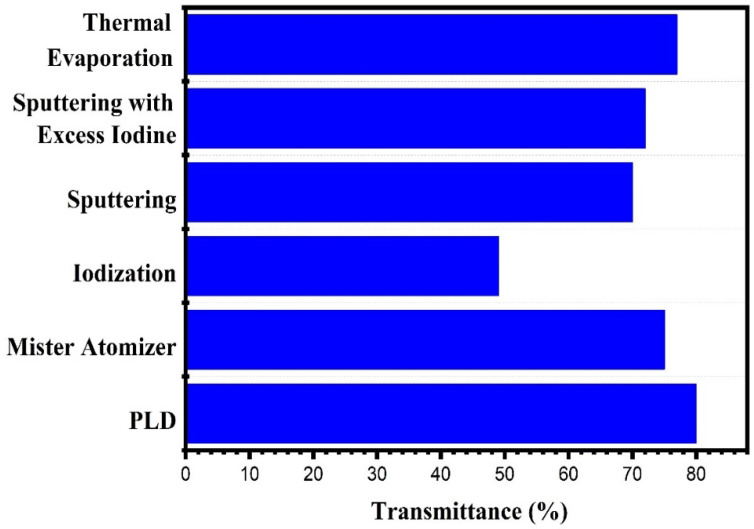
Changes in the transmittance with different deposition techniques reported by other authors.

Several authors have reported that the deposition technique impacts the transmittance. Kaushik et al. [56] investigated the CuI-thin-film using thermal evaporation. They found an increased transmission of 70% in the 550–1000 nm wavelength range compared to previous publications reported by Potts et al. [356] and Burns et al. [357]. Figure 37 is plotted using available data reported by other authors to show the changes in the transmittance for different deposition techniques [56,197,198,200,358].

##### Effect of Temperature

**Figure 38:**
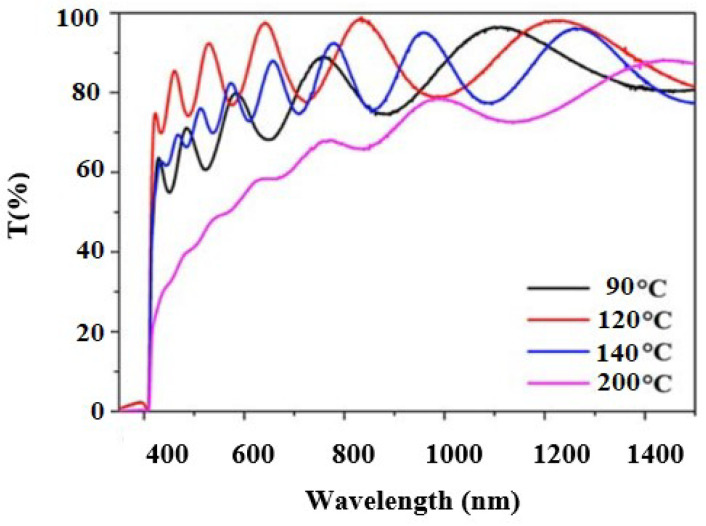
Transmittance spectra of CuI thin films deposited at various substrate temperatures. Reproduced from [359] under permissions from copyright clearance center.

Zi et al. [359] and Zhu and Zhao [190] reported the transmittance spectra of CuI films produced at various substrate temperatures. Similar results were obtained, as shown in Figure 38, wherein the transmittance increased as the substrate temperature rose. CuI-thin-film structure and characteristics deteriorate with rising substrate temperature, as indicated by phase structure breaking and average transmittance falling. The I/Cu ratio and inadvertent oxygen introduction are thought to be important determinants of the conductive characteristics.

**Figure 39:**
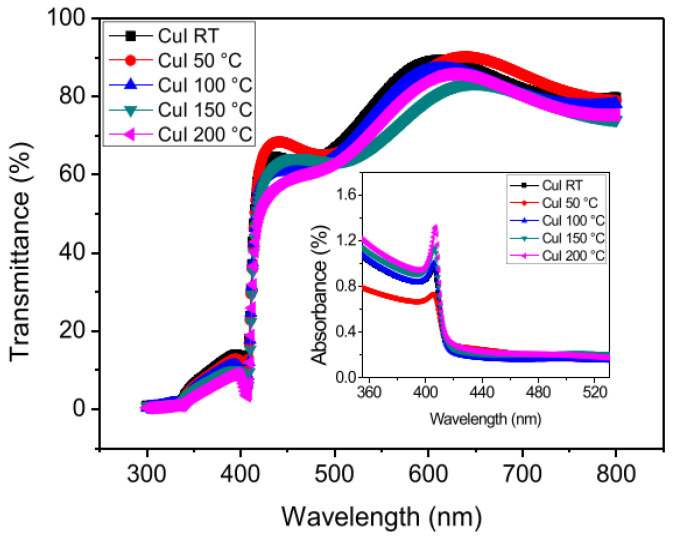
CuI thin-film transmission spectra at various annealing temperatures. The absorption spectra of CuI thin films are displayed in the inset. Reproduced from [54] under permissions from copyright clearance center.

Another study [54] found a similar observation, showing that the transmittance decreased with the increasing annealing temperature, as shown in Figure 39. With an average transmittance of roughly 70–80%, all films are extremely transparent in the visible spectrum. At 411 nm, a distinct absorption edge is observed. All the films’ surfaces showed interference patterns, indicating they were specular and homogenous [360].

##### Effect of Doping Concentration

**Figure 40:**
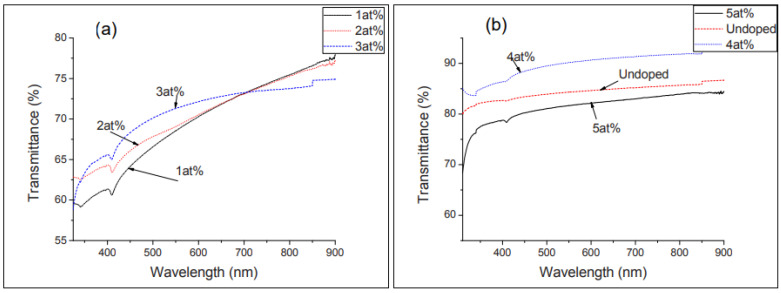
CuI film transmissivity spectra at (**a**) 1 at. %, 2 at. %, and 3 at. %; (**b**) undoped, 4 at. %, and 5 at. %. Reproduced from [191] under permissions from copyright clearance center.

Amalina et al. [191] investigated the effects of I_2_ doping on CuI thin films. Figure 40a,b display the optical transmission spectra of CuI thin films produced on a glass substrate for 1–5% doping. All films show high transparency in the visible range of the optical spectrum. The excitation of electrons from valence band sub-bands to the conduction band may be the cause of the 410 nm hump that has been observed [191]. The enhanced photon scattering by doping-induced crystal defects may cause a transmittance drop of up to 3 at. % [216]. The electrical properties, which rose to 3.0% of the doping concentration in the work by Amalina et al. [191], support this. Transmittance increased after 4 at. % I_2_ doping, parallel to the resistivity value.

#### 4.2.4. Polymer Thin Films

##### Effect of Wavelength

In the case of polymeric materials, the transmittance shows a higher value in the short wavelength. In work [361], thin films of different polymeric PMMA were created by slowly evaporating a chloroform suspension of the composites. It was discovered that 20% of the composites was the ideal concentration, resulting in films with good visual transparency that were 0.1 mm thick. The silver nano prism (SNP) is responsible for the strong absorbance observed in the transmission spectra at shorter wavelengths, where the peak for the SNP-2 system is broader and reaches its maximum at a higher wavelength of 880 nm than for the SNP-1 system (740 nm); this is comparable to the absorbance of the SNPs alone. However, light scattering and decreased transmittance might result from rough surfaces at the interfaces between the polymer film and substrate. Smooth interfaces help the film to transmit light more effectively.

##### Effect of Doping Concentrations

A study [362] examined the impact of different ZnO concentrations on the optical characteristics of pure poly-vinylidene fluoride (PVDF) and PVDF-ZnO nanocomposites. The transmittance of ultraviolet light reduced as the ZnO content in the nanocomposite films increased according to the UV–vis transmittance spectra of PVDF pure and PVDF-ZnO nanocomposites. The increase in surface roughness is the cause of the observed lower transmittance. ZnO drives the nanocomposite sample’s notable drop in transmittance nanoparticle-induced Rayleigh scattering [362]. The optical characteristics of polymer composites can be altered by adding dopants or additives. For instance, some additions might make the thin film more transparent or provide it the ability to absorb or emit light.

##### Effect of Polymer Type and Molecular Structure

Polymers exhibit different optical qualities depending on their molecular weight, chain architecture, and chemical composition. For example, because they absorb less light, polymers having aromatic or conjugated structures may have better optical transmittance [363]. A common polymer with good optical transparency in the visible range is polyethylene [364]. Light can travel through it without absorption or scattering because of its linear molecular structure comprising repeated ethylene units (-CH_2_-CH_2_-). Because of this, polyethylene thin films have outstanding optical transmittance, making them appropriate for protective coatings, lenses, and food packaging.

Polystyrene (PS) is another typical polymer known for transparency and optical clarity [365]. It is composed of an ethylene-linked chain of aromatic benzene rings. The stiff and planar structure of the benzene rings facilitates the efficient transmission of visible light through the polymer matrix. When comparing high molecular weight PS to its lower molecular weight competitors, PS shows better optical transmittance. PS thin films with longer polymer chains are better suited for high optical clarity applications like transparent packaging and optical lenses because they are less flawed and show less light scattering [366]. PMMA is an optically transparent polymer with a linear chain topology. PMMA thin films have a homogeneous molecular structure and little chain branching or crosslinking; good PMMA is frequently utilized in optical applications, including light guide panels and eyeglass lenses, because it offers optical clarity and high transmittance throughout the visible spectrum [367].

##### Effect of Film Thickness

Reduced transmittance is often the result of thicker polymer sheets’ tendency to absorb lighter and scatter photons [368]. In the visible spectrum, thin polymer films (less than 100 nm) could transmit higher. More material for incident light to pass through is present in thicker polymer films, which increases the likelihood of light absorption inside the film. Photons may interact with molecular bonds, functional groups, or contaminants in the polymer film during light transmission, resulting in photon absorption and decreased transmittance. The polymer molecules’ absorption of light energy reduces the intensity of transmitted light [369].

Due to its low light absorption, a thin polyethylene terephthalate (PET) sheet as thin as a few micrometers may have good transmittance throughout the visible spectrum. Nevertheless, the polymer material absorbs more incident light as the PET film thickness rises, which lowers optical clarity and transmittance [370].

Reduced transmittance might result from introducing structural imperfections, surface roughness, or internal interfaces caused by thicker polymer sheets. These factors can scatter incident light [370,371]. When photons interact with changes in the polymer film’s refractive index or surface morphology, they diverge from their intended route and scatter in various directions. This process is known as light scattering. Consequently, there is a reduction in optical transparency due to increased attenuation and perhaps diffuse dispersion of the transmitted light. A thin polycarbonate (PC) sheet with good transmittance and low light scattering may have smooth surfaces and a consistent thickness [371]. However, light scattering intensifies with increasing PC film thickness or if surface imperfections are introduced during manufacture, resulting in decreased transmittance and compromised optical performance.

#### 4.2.5. INO Thin Film

##### Effect of Temperature

**Figure 41:**
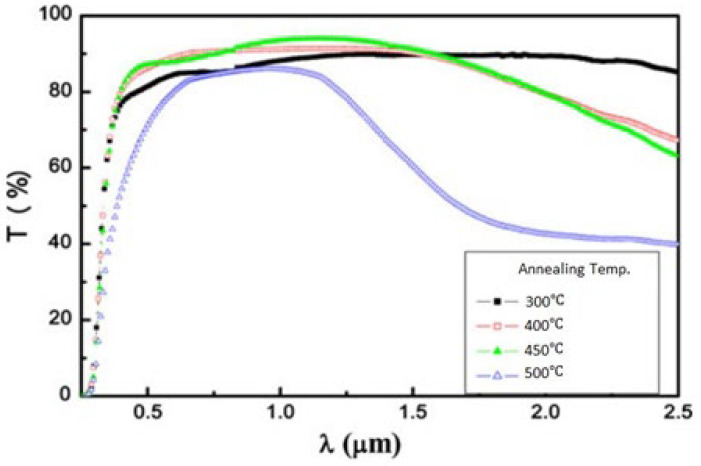
Variations in transmittance with wavelength for INO. Reproduced from [372] under permissions from copyright clearance center.

Numerous studies have been conducted on the transmittance affected by temperature. Figure 41 shows an investigation conducted by Solieman [372] on the changes in the transmittance with different annealing temperatures. It shows that, in the visible spectrum, all films exhibited high transparency; however, transmittance values drop in the longer wavelength range as the sintering temperature increases. The sample’s fluctuating oxygen content could be the cause of this. In addition, the films showed high transparency (80%) in the visible range. Films sintered at 500 °C displayed a narrower transparent window than those sintered at 300 °C, which may have exceeded the device’s detection limit. As the sintering temperature rose, the NIR transmission dropped.

**Figure 42:**
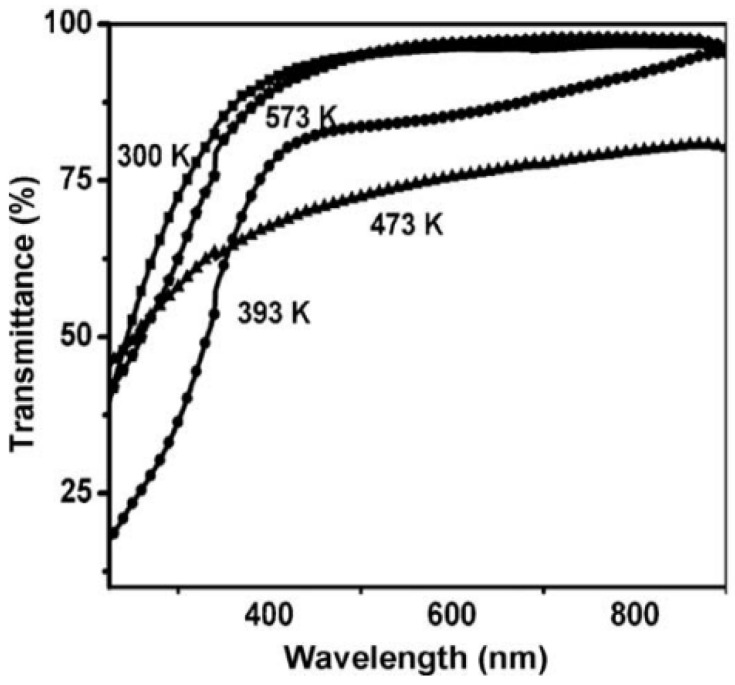
Changes in the transmittance with changes in the substrate temperature for INO. Reproduced from [342] under permissions from copyright clearance center.

Figure 42 shows the effects of the transmittance with the changing substrate temperature. Stress was placed on crystalline films when produced at ambient temperature on amorphous substrates (like quartz). As a result, the films may not be stoichiometric and will typically only exhibit poor transparency [342]. Figure 42 also shows that the transmittance of films created at 300 K is high, approximately 96%. It is interesting to note that this film has the maximum thickness value. Its high transmittance may be due to the smooth surface feature. The study by Beena et al. [342] also reported that the following elements generally cause a decrease in the film’s transmittance: (i) mixed phases, (ii) thickness growth, (iii) defects and oxygen vacancies, (iv) significant root mean square (RMS) surface roughness; (v) porous nature of the films; (vi) grain boundary scattering, etc. However, in this instance, the films only display a uniform crystalline phase, and the film thickness decreases with substrate temperature. When substrate temperature rises, oxygen desorption may occur, creating oxygen vacancies. Therefore, the film created at T_s_ = 573 K should have the lowest transmittance value and more oxygen vacancies.

**Figure 43:**
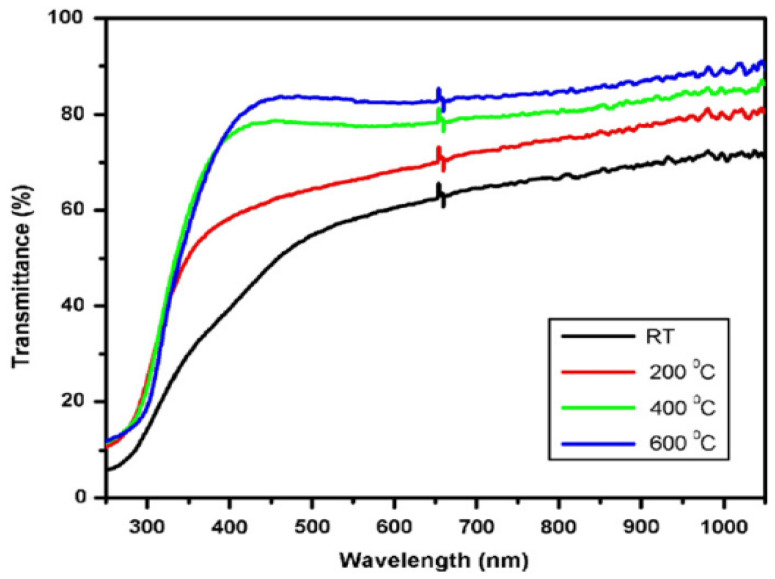
Effects of temperature on the transmittance for INO. Reproduced from [344] under permissions from copyright clearance center.

A similar study by Gupta et al. [344] illustrated how the substrate’s temperature affects the films’ optical transmittance, as shown in Figure 43. It is clear from Figure 43 that the growing temperature affects film transparency. We find that, as substrate temperature rises, the films’ transmittance also increases. In this study, the highest transmittance was found to be 84%.

Another investigation by Senthilkumar et al. [345] revealed that the UV–vis region shows a notable increase in average transmittance as the annealing temperature rises from 350 to 500 °C. They suggested that this could be the consequence of the films’ increased crystallinity upon annealing, which is likely related to XRD examination. Their crystallinity likely influences the transparency of the films’ light scattering. Better transparency results from decreased light scattering as the films’ crystallinity increases. According to Han et al. [373], the thin films’ electrical and transmittance qualities are improved by crystallization. When used as transparent electrodes in optoelectronic devices, In_2_O_3_ thin films are anticipated to have a high transparency in the visible spectrum.

##### Effect of Doping Concentration

**Figure 44:**
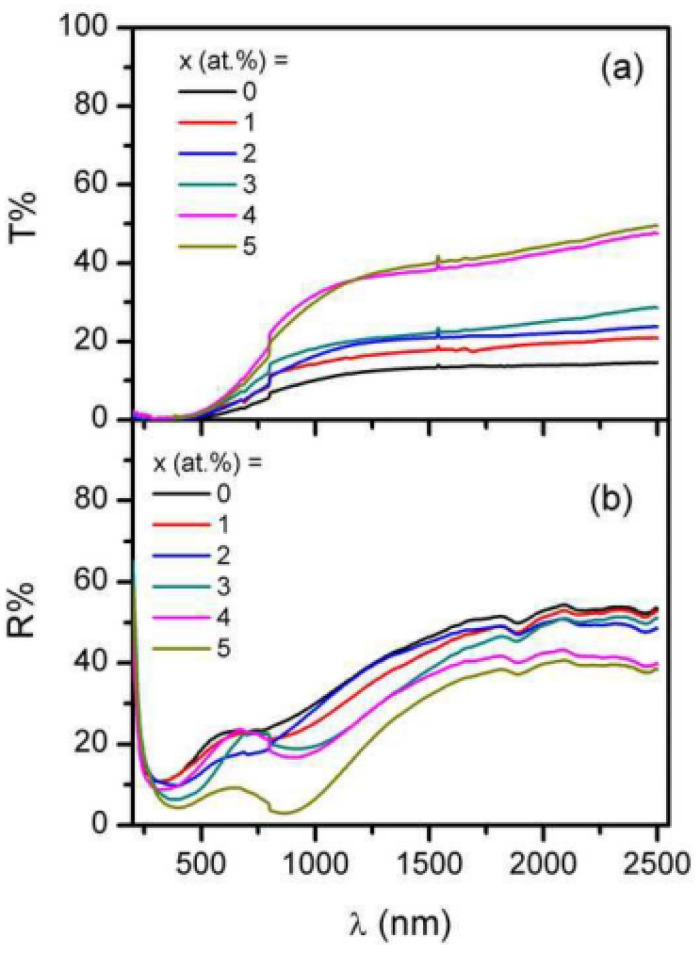
Changes in (**a**) transmittance and (**b**) reflectance with the increasing doping concentration of vanadium. Reproduced from [374] under permissions from copyright clearance center.

The effects of Sn’s doping have already been mentioned in Section 4.2.2. Aside from that, another doping element can also influence the optical properties of In_2_O_3_. A study [374] reported that the transmittance of undoped In_2_O_3_ was the lowest, and it rose as the V doping level increased. The oxygen lost during evaporation is the reason for the poor transmittance of the undoped In_2_O_3_. Following doping, the V-doped In_2_O_3_ films exhibit an increase in transmittance and a decrease in reflectance, as shown in Figure 44a,b. It is possible to attribute the rise in transmittance with higher V doping relating to improved stoichiometry.

#### 4.2.6. CdO Thin Film

Researchers are most attracted to CdO’s enormous energy bandgap, significant transmissivity in the visible spectrum, remarkable luminescence characteristics, and other features. These characteristics have made it suitable for use in the creation of liquid crystal displays, IR detectors, anti-reflection coatings, photodiodes, phototransistors, photovoltaic cells, diaphanous electrodes, and TCOs [374,375,376].

##### Effect of Oxygen Pressure

**Figure 45:**
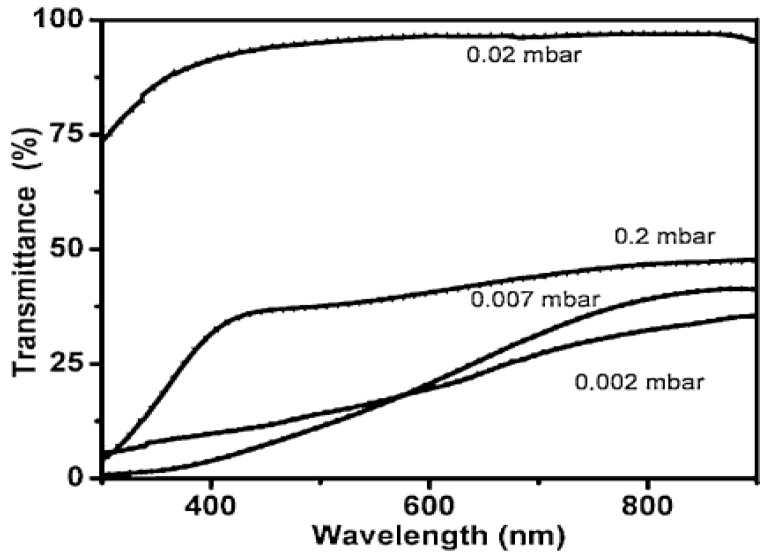
Variations in transmittance with changing oxygen pressure. Reproduced from [342] under permissions from copyright clearance center.

The oxygen pressure can influence the transmittance of the CdO thin film. Beena et al. [342] reported the variations in the transmittance with different oxygen pressures in their research, as shown in Figure 45. The films formed at lower oxygen pressures of 0.002 and 0.007 mbar displayed poorer transmittance values. This might be attributed to oxygen deficits and the absence of stoichiometry in the film, which is caused by an inadequate oxygen ambiance. A stoichiometric film devoid of oxygen deficits can be achieved at elevated oxygen ambient levels. The quality of the film may decrease if the background oxygen pressure rises over this threshold because of the increased collision between laser plume species and background oxygen atoms. While the films deposited at an oxygen pressure of 0.2 mbar exhibits a lesser transmittance, the films formed at an oxygen ambiance of 0.02 mbar show a very high transmittance. The film that was deposited at 0.02 mbar demonstrated a significantly higher transmittance than the other films, indicating its superior quality. Their previous study on ITO films determined the ideal background oxygen pressure for producing a high-quality film to be 0.02 mbar [375].

##### Effect of Temperature

**Figure 46:**
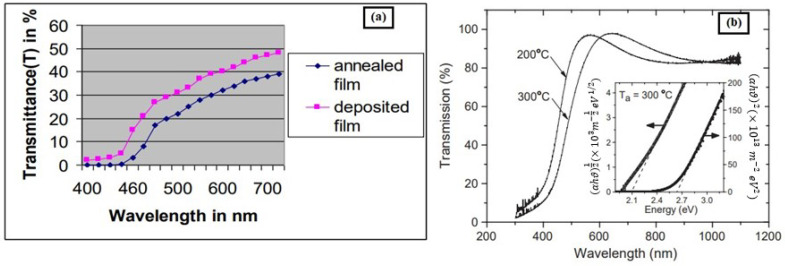
Variations in transmittance with annealing temperature. Reproduced (**a**) from [333]; (**b**) from [334] under permissions from copyright clearance center.

Ortega [376] reported that the n-type CdO thin films exhibit a 2.2 eV bandgap and a rock salt structure. Additionally, it records strong optical transmission and conductivity within the visible light spectrum. According to a study [333], the transmittance of deposited and annealed films rises non-linearly with wavelength, as shown in Figure 46a. Additionally, the transmittance of deposited films is higher than that of annealed films at the same wavelength. A similar investigation was conducted by Santos-Cruz et al. [334], where they investigated the changes in the transmittance for the annealing temperature range of 200–450 °C. They reported that all the films showed high transparency, around 85%, as shown in Figure 46b. It is also observed that, as a film is annealed at a higher temperature, the absorption edge changes to higher wavelengths. For every film, this pattern is observed.

##### Effect of Doping Concentration

**Figure 47:**
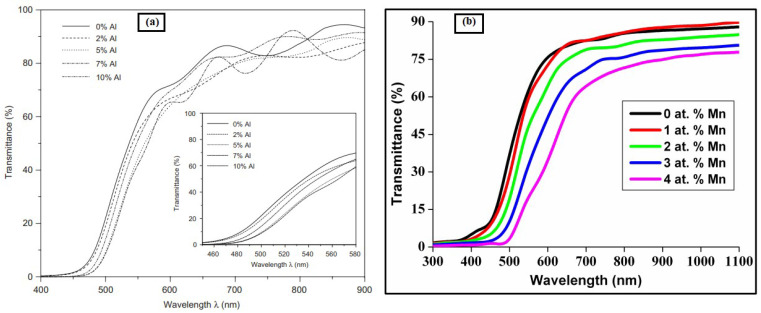
Changes in the transmittance with increasing (**a**) Al dopant [377] and (**b**) Mn dopant [378] in CdO thin films. Reproduced from [377,378] under permissions from copyright clearance center.

A study [377] investigated the variations in the transmittance for doping up to 10 wt.% of Al, and it was evident that the transparency falls between 70 and 80% in the visible spectrum. The investigation showed the graphical representation of the variations in transmittance, as shown in Figure 47a. Another study was conducted by Manjula et al. [378], where the impact on the transmittance was investigated with Mn dopant. Figure 47b displays the transmittance spectra of thin films of CdO: Mn that have been formed at various concentrations of Mn doping (0 to 4 at. %). Transparency for the pure CdO film is 88%, increasing to 90% for the CdO film doped with 1% Mn. Higher Mn doping concentrations (more than 1 at. %) decrease transparency; the film coated with 4 at. % Mn achieves the lowest value of 78%. As the Mn doping concentration rises, the transmission edge changes to the longer wavelength side, indicating a decrease in the optical bandgap of the CdO: Mn films. The reduction in transparency observed in CdO: Mn films coated with 2, 3, and 4 at. % Mn concentrations could potentially be attributed to the addition of Mn to the CdO lattice, together with a rise in the concentration of free charge carriers [379].

**Figure 48:**
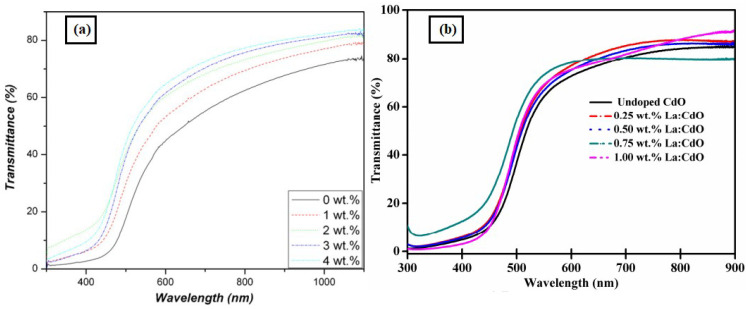
Influence of (**a**) Al dopant [335] and (**b**) La dopant [380] on the transmittance of CdO thin films. Reproduced from [335,380] under permissions from copyright clearance center.

Another study [335] investigating the influence of Al dopant on optical transmittance reported that adding Al dopant into CdO-thin-film improves transmittance. The transmittance of the undoped films is 62%. In contrast, the 4 wt. % Al doping raises the transmittance value to 77%, as shown in Figure 48a. A similar study by Ziabari et al. [339] demonstrated the transmittance spectra that, at all doping levels, the addition of Al to CdO enhances transmission. More precisely, the transmittance of the doped film contains 1 at. % Al is close to 90%, which is higher than the doped film containing 5 at. %. This could be because the 1 at % doped film has a comparatively lower RMS roughness than the 5 at % doped films, a parameter that could reduce optical scattering.

An investigation by Velusamy et al. [340] showed the effects of La dopant on the transmittance of CdO thin films. They reported that the CdO film’s transmittance increased to 0.5 wt. % of La doping, but it decreased at 0.75 and 1 wt. (%) of doping, as shown in Figure 48b. Compared to undoped CdO films, the optical analysis reveals that La-doped CdO thin films have comparatively high transmittance, suggesting its optoelectronic applications.

The influence of another doping element, Mg, on the transmittance was investigated by Usharani et al. [338], where it is reported that the transparency increases significantly, with the exception of the CdO film coated with 2 at. % Mg. At wavelengths longer than 500 nm, the maximum transmittance of the CdO: Mg film coated with 8 at. % Mg is almost equivalent to 90%. The doped films’ high transmittance suggests they have a high degree of stoichiometry and are very crystalline. Regarding solar cell applications, CdO: Mg films are regarded as excellent window materials because of their exceptional transparency in both the visible and infrared areas. The film coated with 2 at. % Mg doping may have a higher thickness, which could account for its low transparency. A higher crystallite size value for this film may cause the increased absorption by free carriers brought about by increased film thickness [381]. The optical transmittance spectra of CdO: Mg films exhibit a movement of the band edge towards low energy, indicating that their bandgaps should rise, with the exception of the CdO: Mg film coated with 2 at. % of Mg dopant. Table 8 shows the overview of the different factors that impacted the transmittance of different conductive thin films through other authors’ reports.

### 4.3. Reflectance

The amount of light reflected from a thin film’s surface is known as its optical reflectance. It is a measurement, usually provided as a percentage, of the ratio of incident light intensity to reflected light intensity. Spectrophotometry and ellipsometry are frequently used to quantify optical reflectance [383]. For various applications, including anti-reflection coatings, optical filters, mirrors, and photonic devices, these measurements offer essential information regarding the optical characteristics of thin films. Optimizing thin-film performance in these applications requires a thorough understanding of and control over optical reflectance [384]. Table 9 shows the overview of the different factors that impacted the reflectance of different conductive thin films through other authors’ reports.

#### 4.3.1. AZO Thin Film

AZO thin films are frequently employed as transparent conductive coatings in various optoelectronic devices, including touchscreens, flat-panel displays, and solar cells. Reduced reflectance is essential in these applications to improve light transmission through the device [385]. Excessive reflectivity may cause loss of incident light, lowering the device’s effectiveness and performance.

##### Effect of Temperature

According to an investigation [222], the annealing temperature impacted the coated samples’ infrared reflection spectrum. The findings show that the annealing temperature significantly affects the AZO films’ surface morphology and crystallization. After annealing at 300 °C, the films exhibit improved surface morphology, including smooth, compacted, and crystallization. Samples with an infrared reflection rate of 30% and 40% were obtained because the IR reflection of the AZO films was directly correlated with the film resistivity.

**Figure 49:**
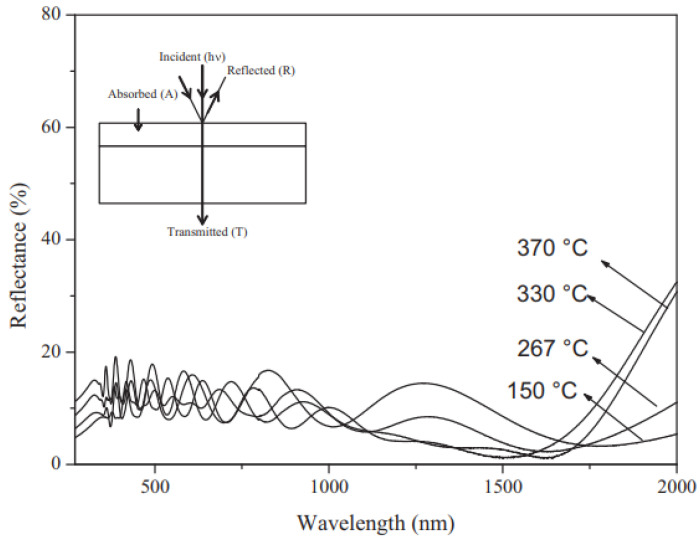
The relationship between reflectance and substrate temperature for ZnO: Al films formed at 150–370 °C. Reproduced from [221] under permissions from copyright clearance center.

The low optical reflection of ZnO: Al thin films is confirmed by the reflectance spectra displayed in Figure 49 from the study [221]. The visible range has a reflectance of roughly 10%. When the substrate temperature rises in the 150–330 °C range, ZnO: Al films exhibit an increase in IR reflectance for wavelengths higher than 1600 nm. At 370 °C, the reflectance drops somewhat, most likely due to the decrease in grain size mentioned previously. Fu et al. [386] discovered the similar behavior of IR reflectance change with substrate temperature. The shift in carrier mobility was used to explain the decrease in IR reflectivity at higher temperatures.

##### Effect of Carrier Concentration

According to the classic Drude model [387], the vibration plasma, which is based on the carrier concentration N and reflectance *R*, is correlated as mentioned in Equation (19),

(19) $$R=1-4(\frac{\varepsilon_{0}C}{ed})\times \frac{1}{N\mu }$$

This equation states that IR reflectance increases as product *N* increases, where the permittivity of empty space, light velocity, electronic charge, film thickness, and carrier mobility are represented by the variables ε_0_, *C*, *e*, *d*, and *μ*, respectively. As previously demonstrated, substrate temperature increases carrier concentration and carrier mobility. Therefore, we can interpret the rise in IR reflectance with substrate temperature in the 150–330 °C range regarding the improvement in product *N*.

##### Effect of Bandgap

According to the study [349], the Kubelka–Munk formula was used to determine the optical bandgap of the films based on their reflectance spectra. By converting the reflectance data, we can compute the energy gap of thin films on nontransparent substrates using the Kubelka–Munk theory. The Kubelka–Munk function *F*(*R*) is correlated with the sample’s diffuse reflectance. Using the relationship, the Kubelka–Munk function was used to calculate the diffuse reflectance data as mentioned in Equation (20) [349],

(20) $$F\left(R\right)=\frac{{\left(1-R\right)}^{2}}{2R}=\frac{\alpha }{s}$$

Here, s is the scattering coefficient, *R* is the sample’s diffuse reflectance, and *F*(*R*) is the Kubelka–Munk function related to the absorbance.

Takci et al. [349] showed that the bandgap value decreases with an Al concentration of 6 and 10 at. %, while it increases with an increase in Al concentration from 0 to 4 at. %. Al-doped ZnO thin films developed at ambient temperature on p-type Si substrate could be viable for optoelectronic device applications.

##### Effect of Film Thickness

Research [230] examined the optical reflectance in the 200–800 nm wavelength range where the AZO thin films were produced on glass substrates using a PLD approach. It was revealed that the structural, optical, and electrical characteristics of AZO thin films were significantly impacted by the film’s thickness. It was also found that the reflectance values ranged from 10% to 20% in wavelengths between 200 and 800 nm. Since the aspect ratio of crystallites varies with thickness, the change in optical reflectance may result from morphological changes in films.

#### 4.3.2. ITO Thin Film

##### Effect of Film Thickness

A study [223] lists the values of a few optical characteristics of (ITO)_1-x_(ZnO)_x_ films at various x ratios in the visible spectrum (320–800 nm), where it is found that the refractive index and free carrier concentration exhibit comparable behaviors. These parameters rise as the ZnO ratio increases to x = 0.15, after which they drop slightly. This could be related to the change in reflectance as the ZnO ratio rises. The reflectance (R%) against wavelength for various ZnO ratios is shown in the study [223]. The reflectance rises as the value of x increases, reaching its maximum value of 50.6% in the near-IR range for x = 0.15. The reflectance begins to diminish as the x value increases further.

The study [221] measured the film’s transmission and reflection, revealing that the prepared thin-film thickness primarily influences transmittance. However, the films’ reflectance spectra are the same. It indicates a slight increase in reflectance for wavelengths greater than 1900 nm. Nevertheless, the drop in transmittance in the same region does not correspond with this rise in reflectance.

##### Effect of Oxygen Pressure

The oxygen pressure during deposition had an impact on the films’ crystalline structure as well. The XRD patterns of the films formed at 300 °C under various oxygen pressures were displayed in a study [231]. The patterns show that the intensity of the (222) peak increases with increasing oxygen pressure from 1 to 100 mTorr, suggesting that the films are more crystalline and retain their preferred orientation. Furthermore, the linewidth of the (222) reflection decreases with increasing oxygen pressure, indicating that the film’s grain size is increasing.

##### Effect of Tin-Dopant Concentration

Tynell et al. [231] suggested that the reflectance strongly relates to the dopant concentration of Sn. The material’s band structure is changed by Sn doping, which also affects the material’s optical transparency, absorption coefficient, and refractive index. The optical response of the material, particularly its reflectance behavior, can be affected by the concentration of Sn dopants. ITO thin-film electrical conductivity can be changed by Sn doping, which may impact the reflectance characteristics of the films. The material’s optical characteristics and light interaction can be affected by altering the carrier density, mobility, and scattering mechanisms brought about by Sn doping.

##### Effect of Temperature

Miao et al. [222] displayed some films’ specular reflectance (SR) and diffuse reflectance (DR). Additionally, it provides the spectra for various substrate temperatures and bias voltages. Specular reflectance intensity changes with wavelength in a manner akin to DR. Transmittance increases due to a drop in specular reflectance brought on by increases in substrate temperature and negative bias voltage. Total integrated scattering (TIS) can calculate the surface roughness (d) using the specular and diffuse reflectance spectra.

#### 4.3.3. CdO Thin Film

##### Effect of Doping

**Figure 50:**
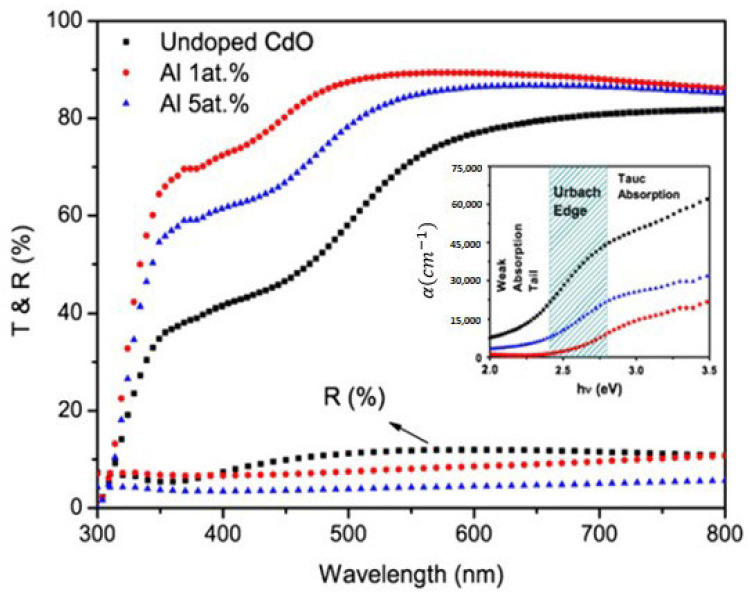
Variations in CdO’s reflectance with the doping concertation of Al. Reproduced from [339] under permissions from copyright clearance center.

Ziabari et al. [339] investigated the effects of Al doping concentration on reflectance. The reflectance curves are shown to be reasonably near one another, with a magnitude difference of less than 10%. In contrast to the 5 at. % Al-doped film, the 1 at. % Al-doped film has a little greater reflectivity. Figure 50 shows variations in CdO’s reflectance with the doping concertation of Al [339]. 

##### Effect of Surface Morphology

The study [339] suggested the impact of surface morphology in the reflectance with the doping concentration. A surface’s reflectance is a highly responsive function of its roughness [388]. In spite of random scattering, lower roughness and more homogenous thickness (of a 1 at. % Al-doped sample) cause electromagnetic beam reflection to occur in almost the same direction, increasing reflectance (compared to a 5 at. % Al film) [389].

#### 4.3.4. INO Thin Film

##### Effect of Doping

A study [374] investigated the variations in the reflectance based on the doping concentration and reported that, following doping, the V-doped In_2_O_3_ films exhibit an increase in transmittance and a decrease in reflectance, as shown in Figure 44b. The NIR area has high reflectance values because of free carrier reflection.

##### Effect of Temperature

**Figure 51:**
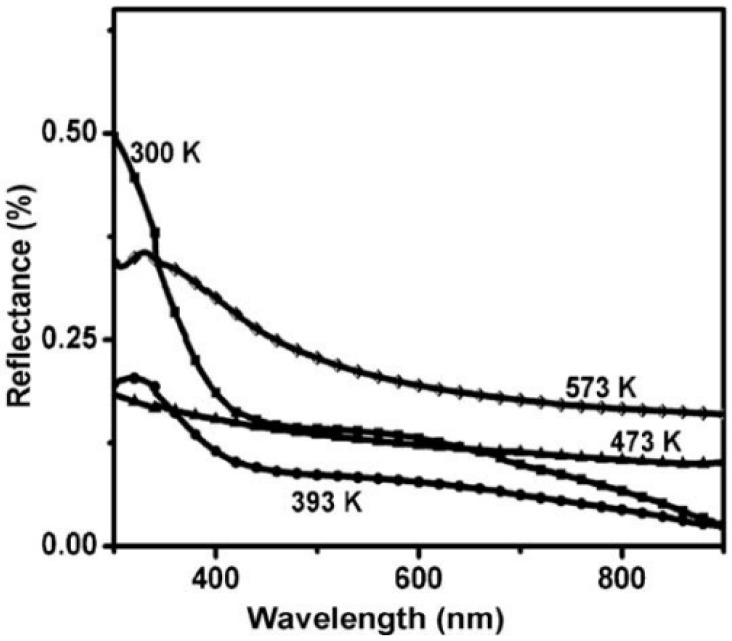
Changes in the reflectance with substrate temperature. Reproduced from [342] under permissions from copyright clearance center.

One of the most effective methods for comprehending crystalline and amorphous materials’ band structure and energy gap is the analysis of optical reflectance spectra. Figure 51 from an article by Beena et al. [342] illustrates how the optical transmission and reflection spectra of the thin films under investigation depend on the substrate temperature. The investigation shows that the thin film has the highest reflectance at a substrate temperature of 300 K in the low wavelength region. In contrast, it has the lowest reflectance in the high wavelength region. However, in the wavelength range around 450–800 nm, the reflectance shows higher values with increasing substrate temperature.

**Table 9:** An overview of the impact of major factors on the reflectance of conductive thin films.

Thin Films	Factors	Effects on Reflectance	Authors
AZO	Temperature	The annealing temperature significantly affects the AZO films’ surface morphology and crystallization. After annealing at 300 °C, the films exhibit improved surface morphology, including smooth, compacted, and crystallization, and samples with an infrared reflection rate of 30% and 40% were obtained.	Miao et al. [222]
		The optical reflection of ZnO: Al thin films is low The visible range has a reflectance of roughly 10%. When the substrate temperature rises in the 150–330 °C range, AZO films exhibit an increase in IR reflectance for wavelengths higher than 1600 nm. At 370 °C the reflectance drops, most likely due to the decrease in grain size.	Mosbah and Aida [221]
		The reflectance increases with the increasing substrate temperature in the 150–330 °C range. At higher temperatures, the reflectance decreases due to the shift in carrier mobility.	Fu et al. [386]
	Carrier concentration	IR reflectance increases as product N increases.	Chaabouni et al. [387]
	Bandgap	The bandgap value decreases with an Al concentration of 6 and 10 at. %, while it increases with an increase in Al concentration from 0 to 4 at. %.	Takci et al. [349]
	Film thickness	Since the aspect ratio of crystallites varies with thickness, the change in optical reflectance may result from morphological changes in films.	Kaur et al. [230]
ITO	Oxygen pressure	Reflection decreases with increasing oxygen pressure, indicating that the film’s grain size increases.	Tynell et al. [231]
	Tin dopant	Tin doping changes the material’s band structure and affects its optical transparency, absorption coefficient, and refractive index. Tin doping causes alterations in the carrier density, mobility, and scattering mechanisms that affect the reflectance.	Tynell et al. [231]
	Temperature	Transmittance increases due to a drop in specular reflectance caused by increases in substrate temperature and negative bias voltage.	Miao et al. [222]
CdO	Doping	The reflectance is reasonably consistent, with a magnitude difference of less than 10%. In contrast to the 5 at. % Al-doped film, the 1 at. % Al-doped film has a little greater reflectivity.	Ziabari et al. [339]
	Surface morphology	A surface’s reflectance is a highly responsive function of its roughness. In spite of random scattering, lower roughness and more homogenous thickness (of a 1 at. % Al-doped sample) cause electromagnetic beam reflection to occur in almost the same direction, increasing reflectance (compared to a 5 at. % Al film).	Abdolahzadeh Ziabari et al. [339], Bennett and Porteus [388], Ghodsi et al. [389]
INO	Doping	The V-doped In_2_O_3_ films exhibit an increase in transmittance and a decrease in reflectance. The NIR area has high reflectance values because of free carrier reflection.	Alqahtani et al. [374]
	Temperature	The thin film has the highest reflectance at a substrate temperature of 300 K in the low-wavelength region. In the wavelength range around 450–800 nm, the reflectance shows higher values with increasing substrate temperature	Beena et al. [342]

### 4.4. Refractive Index (RI)

An optical characteristic of a material that characterizes how light travels through it is called the optical RI. It shows the relationship between the speed of light in a substance and the speed of light in a vacuum. The way light propagates through a material and bends or refracts as it enters and exits it is determined by its refractive index [390]. It affects how much light at the film surfaces is absorbed, reflected, or refracted. To obtain desired light transmission properties, it is essential to understand the refractive index of the thin-film material when constructing optical coatings, anti-reflection coatings, and other thin-film structures [391].

Thin optical film includes dispersion, birefringence, and optical dispersion, which are influenced by the RI. Dispersion effects, in which the wavelength-dependent variations in light speed occur, can be caused by changes in the refractive index [295,392]. Birefringence thin films have a distinct refractive index when light is polarized in different directions. Designing thin-film devices with particular optical functionality requires understanding and controlling these optical properties. Overall, the optical refractive index is a fundamental property that controls how light behaves in thin films [392,393]. It is one factor that most affects a solar cell’s efficiency and enables it to minimize optical losses. It is also a critical factor in the engineering and design of thin-film structures used in coatings, filters, waveguides, and photonic devices, among other optical applications [394].

However, one of the essential characteristics of an optical material is its RI, which is directly correlated with the local field inside materials and the electronic polarizability of ions. For use in integrated optic devices, such as switches, filters, modulators, etc., where refractive indices are crucial constants for device design, the assessment of optical materials’ refractive indices is vital [394]. Through other authors’ reports, Table 10 shows the overview of the different factors that impacted the refractive index of different conductive thin films.

#### 4.4.1. AZO Thin Film

##### Effect of Wavelength

A study [322] examined AZO thin films applied by the dip-coating method to Corning glass substrates. It was discovered that the refractive index initially decreases with increasing wavelength and then becomes constant at higher wavelengths. Caglar et al. [215] also illustrated the similar wavelength dependency of refractive index.

##### Effect of Film Thickness

**Figure 52:**
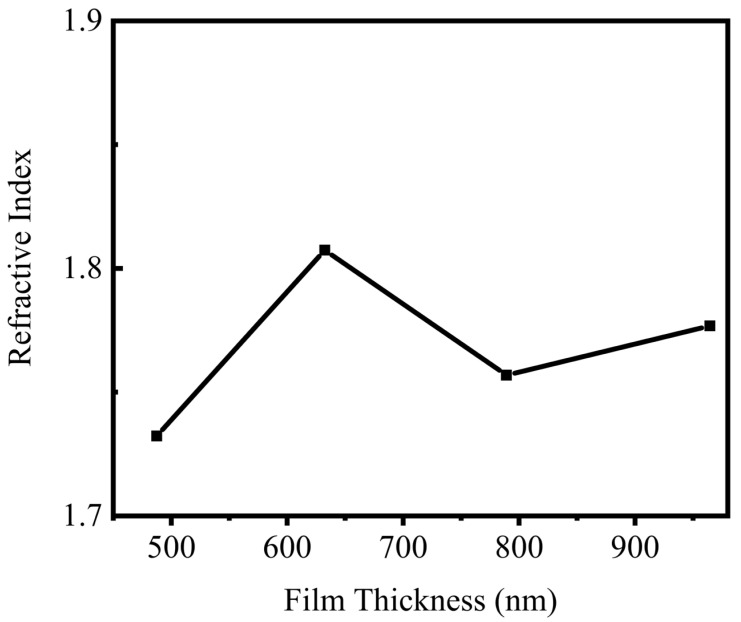
Variations in refractive index with film thickness.

Film thickness influences the optical bandgap and determines the optical transparency and refractive index [219]. Kaur et al. [230] showed that the refractive index increases with film thickness. Figure 52, drawn from the available data of Kaur et al.’s study [230], shows the changes in refractive index with changing thickness. 

**Figure 53:**
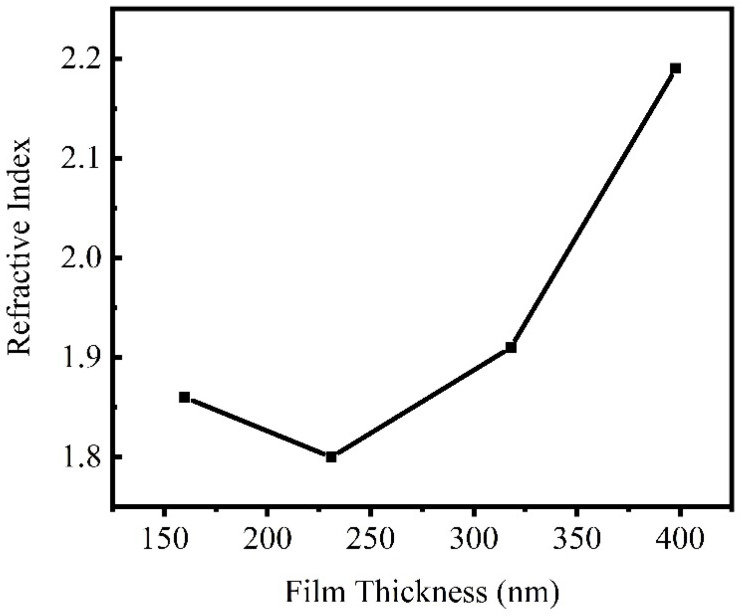
Refractive index vs. film thickness plotted from the data provided by Reddy et al. [223].

Reddy et al. [223] illustrated how the refractive index of AZO thin films changes with film thickness. Figure 53 is plotted using the data from the same study [223], where we can see a trend of refractive index with the film thickness. It can be observed that the ZnO films’ refractive index first dropped from 1.86 to 1.80 as the film thickness decreased from 160 nm to 231 nm. Afterward, the index rose to 2.19 with an increase in film thickness to 398 nm.

The study [230] lists the thin-film thickness and refractive index values. The thickness of the layer causes a rise in the refractive index, n. Optical reflection’s low value of AZO thin films was verified by the reflectance *R*(*k*) spectra presented in the study [219]. The film’s thickness, *d*, can be accurately estimated using the *R*(*k*) reflectance spectra. Equation (21) can be used to determine the thickness *d* of AZO thin films,

(21) $$d=\frac{\lambda_{1}\lambda_{2}}{2(\lambda_{2}n_{1}\left(\lambda_{1}\right)-\lambda_{1}n_{2}\left(\lambda_{2}\right))}$$

##### Effect of Substrate Tilt Angle and Substrate Rotating

A study mentioned in [220] investigated how the substrate tilt angle and substrate rotation affected the refractive index. It was discovered that the refractive index was decreased in the absence of substrate rotation for larger inclined nanocolumnar structures throughout a broad wavelength area of 400–1200 nm. With longer wavelengths, the refractive index decreased to a greater extent. The photon energy trapped in the grain boundary and internal reflections are likely responsible for the decreased refractive index [219]. Furthermore, the decreased refractive index of tilted AZO sheets may be partially attributed to the increased porosity. Due to the low shadow effect brought on by the isotropic deposition characteristic of RF magnetron sputtering as opposed to e-beam evaporation, the refractive index drop is relatively minimal. The refractive index decreased for the tilted nanocolumnar structure, and the extinction coefficient was relatively constant over the visible wavelength range.

##### Effect of Doping

Caglar et al. investigated [215] that, when the concentration of Al doping rises, the refractive index falls. The carrier concentration’s increase in the AZO films may be the primary cause of the refractive index decrease observed with an increase in Al doping concentration. The visible region’s refractive index varies very little across all films, typically remaining constant at ~1.7. The results found by Caglar et al. [215] in the visible region closely resemble those obtained using alternative methods by Bandyopadhyay et al. [395] and Xue et al. [396]. The refractive index is observed to rise dramatically near the optical absorption edge.

##### Effect of Annealing Temperature

According to research [210], ZnO films had a refractive index of about 1.25 in the 2.4–3.2 eV energy range before annealing, and the index progressively increased when the energy reached 3.2 eV. ZnO’s refractive index was around 1.75 in the visual light range when the films were annealed at 600 °C. The findings demonstrated that ZnO’s refractive index rose as annealing temperatures rose.

#### 4.4.2. ITO Thin Film

##### Effect of Doping Concentration

The refractive index decreased with wavelength in the study [202], and it was also found that the Sn-doped In_2_O_3_ thin films had a smaller refractive index than the pure In_2_O_3_ film. As Sn concentration increased, the porosity of the ITO film increased, and the value of grain size decreased, which resulted in a decrease in refractive index.

**Figure 54:**
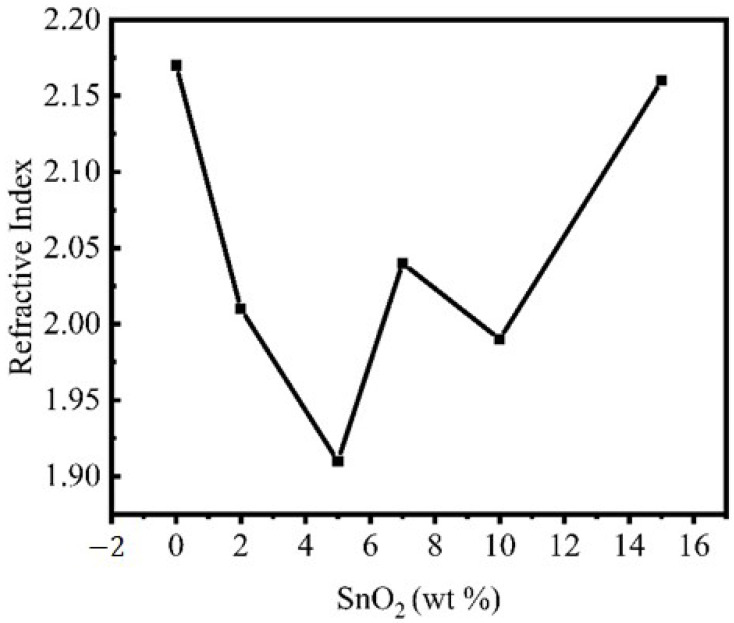
Changes in the refractive index with the changing SnO_2_ content.

According to [270], the effects of Sn doping on the refractive index were shown, and Figure 54 was plotted from the available data of this study. Figure 54 also indicates that the refractive index progressively drops with SnO_2_ content up to 5% of the total weight before significantly increasing to 15%.

The study [270] showed that the value of n dropped to 1.91 when the SnO_2_ content ascended to 5 wt %, and the carrier concentration increased. However, because of a drop in carrier concentration, the value of n increased once again as the SnO_2_ content was raised to a 15% weight percentage. These outcomes are consistent with previously published values [397,398].

##### Effect of Packing Density

There is a correlation between the lower packing density of grains in the films and the lower value of the refractive index, n. Therefore, the refractive index is a valuable tool for determining the packing density from the Bragg and Pippard model [399], which Harris et al. [400] later improved,

(22) $$n^{2}=\frac{\left(1-p\right)n_{v}^{4}+(1+p)n_{v}n_{s}^{2}}{\left(1+p\right)n_{v}^{2}+(1-p)n_{s}^{2}}$$

where packing density is denoted by *p*. Additionally, *n*, *n_v_*, and *n_s_* are the refractive index of the generated film, the void refractive index (equal to one for air), and the substrate refractive index, respectively. It was found that packing density decreased with Sn doping concentration, supporting the findings on the refractive index.

##### Effect of Porosity

The porosity is correlated with the refractive index. The following Equation (23) is employed to determine the porosity.

(23) $$Porosity=(1-\frac{n^{2}-1}{n_{d}^{2}-1})\times 100(\%)$$

where *n* and *n_d_* denote the refractive index of porous thin films and the pore-free films [401]. Senthilkumar et al. [202] demonstrated how the ITO films’ porosity rose as the Sn doping concentration increased.

##### Effect of Power and Deposition Time

Tchenka et al. [325] showed that the refractive index rises with increasing power. Furthermore, because of the effects of thickness and density, the refractive index increases with longer deposition times in the visible and infrared areas. This conclusion aligns with other research findings [398,402].

##### Effect of ZnO Ratio

A study [270] found similarities between the refractive index and free carrier concentration behaviors. The fluctuation of reflectance with increasing ZnO ratio may be connected to these parameters; as the ZnO ratio increases up to x = 0.15, the values show an increase, but with additional increases in the ratio, the values decrease slightly.

##### Effect of Temperature

**Figure 55:**
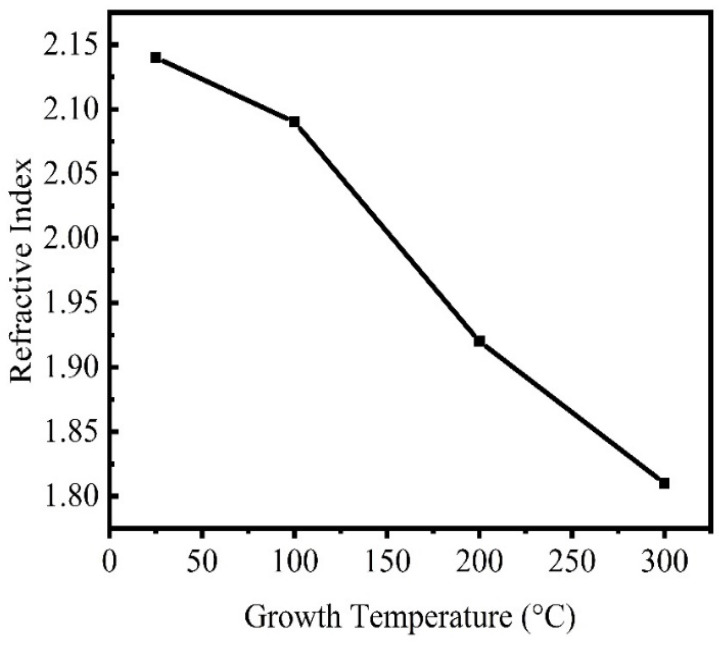
ITO films produced at different temperatures and their refractive indices.

Mohamed et al. [270] showed that the refractive index decreases as the ZnO content and annealing temperature rise. The increase in carrier mobility, which raises the relaxation time and mean free path, could cause the refractive index to decrease. The growing temperature also affects the films’ refractive indices, as shown by Kim et al. [273]. Figure 55 is plotted using the data investigated by Kim et al. [273] in their research, and it is found that the value of n at a wavelength of 550 nm falls between 2.14 and 1.81 when the deposition temperature of substrate rises from 25 to 300 °C. A rise in the films’ carrier concentration can also account for this drop in refractive index. It is also shown in the study that, as the carrier density of the films increased, the deposition temperature rose to 300 °C, and, hence, the value of n decreased.

#### 4.4.3. CdO Thin Film

##### Effect of Doping Concentration

**Figure 56:**
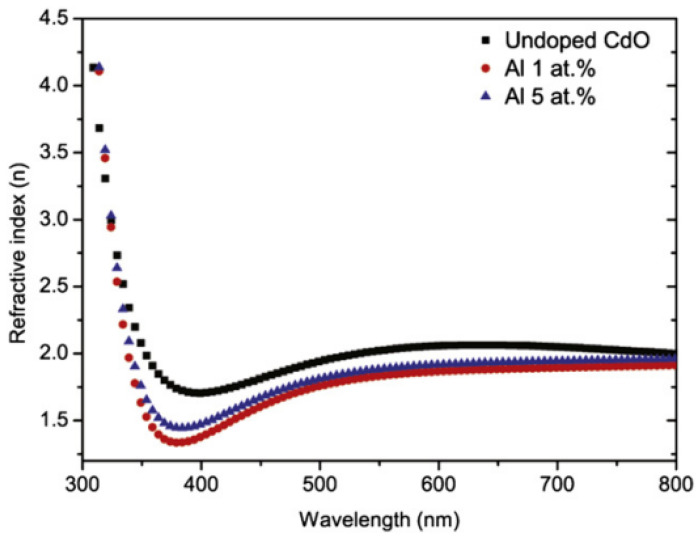
Variations in the refractive index with doping concentrations. Reproduced from [339] under permissions from copyright clearance center.

CdO thin films have a moderate refractive index, good optical transmittance in the visible portion of the solar spectrum, and high electrical conductivity (even without doping), making them suitable for a variety of solar cells, phototransistors, and photodiode applications [377]. Ziabari et al. [339] demonstrated the influence of Al doping on the optical properties of CdO thin films. It is reported that the structural alterations caused by the Al dopant in the films often drop the refractive index. With the highest refractive index and lowest transmittance, this sample is most likely due to the well-packed undoped CdO coating on the surface. This notion is supported by the greater crystallite size and lower hole percentage, which result in lower porosity of the undoped film. In the meantime, 1 at. % Al-doped film has a lower refractive index than 5 at. % as shown in Figure 56.

**Figure 57:**
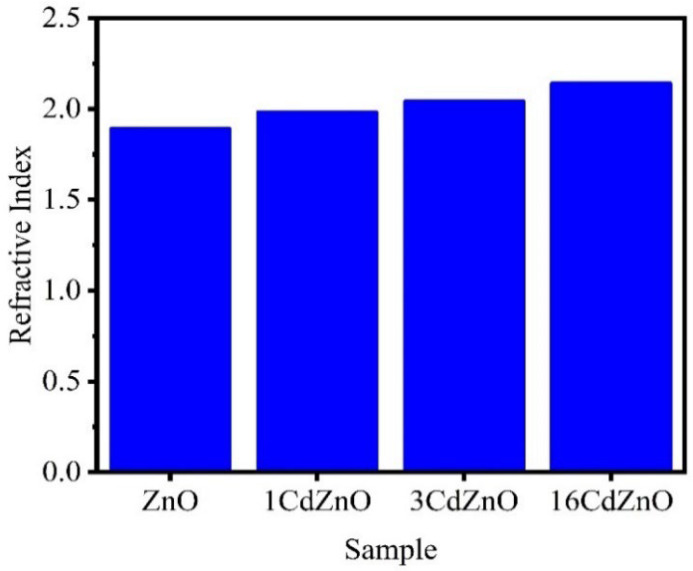
Changes in the refractive index for CdO doping in ZnO thin films.

Vinodkumar et al. [402] investigated the changes in the refractive indices by incorporating CdO into a ZnO thin film. With the increasing amount of CdO doping, the refractive index gradually increased. Figure 57 depicts the influence of the CdO doping on the refractive index.

##### Effect of Roughness

Ziabari et al. [339] reported that the refractive index fluctuations with RMS roughness values were found to be correlated with the film doping level for the doped samples; a high refractive index was associated with higher film roughness, higher Al doping, and smaller grain sizes.

#### 4.4.4. CuI Thin Film

##### Effect of Temperature

Zhu and Zhao [190] reported the temperature effects on the refractive index of CuI thin film. The acquired refractive index of CuI films formed at room temperature falls with increasing substrate temperature and does not significantly vary with laser energy. Within the range between 440 to 1000 nm wavelength, the refractive index is found to be around 1.536–1.520.

##### Effect of Changing the Media of Methodology

**Figure 58:**
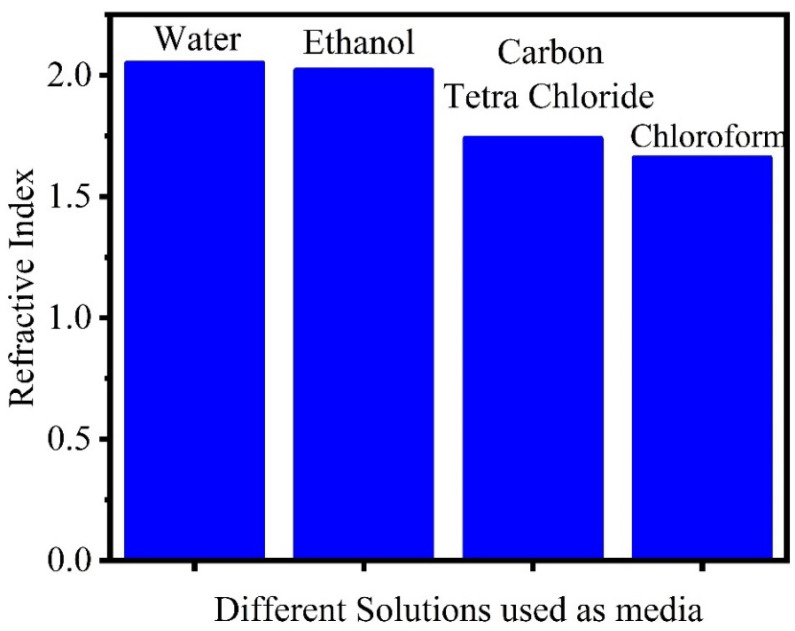
Variations in the refractive index when using different solution media. The column graph is plotted using the data available from Kariper’s investigation.

Kariper [403] demonstrated the effects of altering the materials that are used to create CuI films on the optical and structural characteristics of CuI. Figure 58 shows the variations in the refractive index caused by the changing media. It is suggested that the film generated in the chloroform bath, where the particle size was somewhat smaller than the others, has a lower refractive index.

##### Effect of Film Thickness

The film thickness varies with the refractive index of CuI thin film. A report [403] suggested that, as the film thickness increases, the refractive index also increases.

**Table 10:** An overview of the impact of major factors on the refractive index of conductive thin films.

Thin Films	Factors	Effects on Refractive Index	Authors
AZO	Wavelength	The refractive index initially decreases with increasing wavelength and then becomes constant at higher wavelengths.	Alam and Cameron [214], Caglar et al. [215]
	Film thickness	ZnO films’ refractive index first dropped from 1.86 to 1.80, increasing film thickness from 160 to 231 nm. The index rose to 2.19 with an increase in film thickness to 398 nm	Reddy et al. [223]
	Doping	When the concentration of Al doping rises, the refractive index falls due to the increase in the carrier concentration of ZnO: Al films. The visible region’s refractive index varies very little across all films, typically remaining constant at ~1.7.	Caglar et al. [215]
	Temperature	The refractive index rose as annealing temperatures rose.	Gareso et al. [210]
ITO	Doping	The refractive index decreased with wavelength The Sn-doped In_2_O_3_ thin films had a smaller refractive index than the pure In_2_O_3_ film. As Sn concentration increased, the porosity of the ITO film increased, and the value of grain size decreased, which resulted in a decrease in refractive index.	Senthilkumar et al. [202]
		The refractive index progressively drops with SnO_2_ content up to 5% of the total weight before significantly increasing to 15%.	Mohamed [270], Chen and Robinson [404], Ohhata et al. [405]
		Packing density decreased with Sn doping concentration, which affects the refractive index.	Harris et al. [400]
		As the Sn doping concentration increases, the ITO films’ porosity rises, impacting the RI.	Senthilkumar et al. [202]
	Power and deposition time	The refractive index rises with increasing power and longer deposition times in the visible and infrared areas.	Tchenka et al. [325], Kim et al. [397], Kumar et al. [398]
	ZnO ratio	As the ZnO ratio increases up to x = 0.15, the refractive index increases, but, as the ratio increases further, the values decrease slightly.	Mohamed [270]
	Temperature	The refractive index decreases as the ZnO content and annealing temperature rise. The increase in carrier mobility, which raises the relaxation time and mean free path, could cause the refractive index to decrease.	Mohamed [270]
		The refractive index falls when the substrate’s deposition temperature rises. A rise in the films’ carrier concentration can also account for this drop in refractive index.	Kim et al. [273]
CdO	Doping concentration	Al dopant in the films often causes structural alterations, resulting in decreased refractive index.	Ziabari et al. [339]
		With the increasing amount of CdO doping, the refractive index gradually increased.	Vinodkumar et al. [402]
	Roughness	A high refractive index was associated with higher film roughness, higher Al doping, and smaller grain sizes.	Ziabari et al. [339]
CuI	Temperature	The refractive index of CuI films formed at room temperature falls with increasing substrate temperature and does not significantly vary with laser energy.	Zhu and Zhao [190]
	Changing the media of methodology	The film generated in the chloroform bath, where the particle size was somewhat smaller than the others, has a lower refractive index.	Kariper [403]
	Film thickness	As the film thickness increases, the RI also increases	Kariper [403]

## 5. Electrical Properties of Different Thin Films

The electrical properties of thin films are just as crucial to their performance as their optical characteristics. It is essential to consider how substrate temperature, aluminum doping concentration, film thickness, and other factors affect thin-film electrical resistivity, mobility, and carrier concentration. 

### 5.1. Resistivity

#### 5.1.1. AZO Thin Film

##### Effect of Doping Concentration

The resistivity variations in zinc oxide thin films doped with various aluminum atomic concentrations are displayed in Figure 59 from [214], where the concentrations of aluminum dopant in the solution were annealed at 500 °C in air. It was discovered that the concentration of Al doping significantly impacts their resistance. Up to 0.8 at. %, the film’s resistivity reduced as the Al concentration increased. At 0.8.% Al concentration, the resistivity of ZnO films demonstrated a minimum value of 1.531024 Ω cm. Subsequently, the resistivity began to rise with higher doping.

Goyal et al. [406] explained the resistivity minimum at a certain doping level by demonstrating that substitutional doping of Al^3+^ at the Zn^2+^ site creates one additional free carrier. When the doping level is raised, more dopant atoms occupy zinc atom lattice positions, producing more charge carriers. However, the dopant atoms cannot occupy the zinc sites at a certain doping level. Al atoms incapable of being replaced at zinc sites do not function well as donors. The increased electron scattering results from the interstitial incorporation of Al in the form of Al_2_O_3_ [407], which is caused by higher amounts of Al incorporation. The aluminum atoms may segregate to the grain boundaries as Al_2_O_3_, which can raise the grain boundary barrier. Because of this, the doping concentration peaks when the substitutional doping of aluminum into zinc oxide reaches its peak, but the mobility decreases further due to increased scattering and grain boundary barrier effects.

A study [217] showed that the minimum resistivity for 3 at. % layers deposited at 475 °C is determined by comparing the resistivity of three distinct aluminum compositions. Because a small quantity of Al was ionized into Al^3+^ and replaced Zn^2+^ in the ZnO film, there was an increase in resistivity at greater doping concentrations. As a result, one aluminum atom replaced one zinc atom, producing one free electron [408]. Up to 3 at. %, the carrier concentration rose as the Al concentration did. However, the carrier concentration dropped at concentrations greater than 3 at. % because the growing Al atoms created neutral defects and did not release free electrons [409].

**Figure 60:**
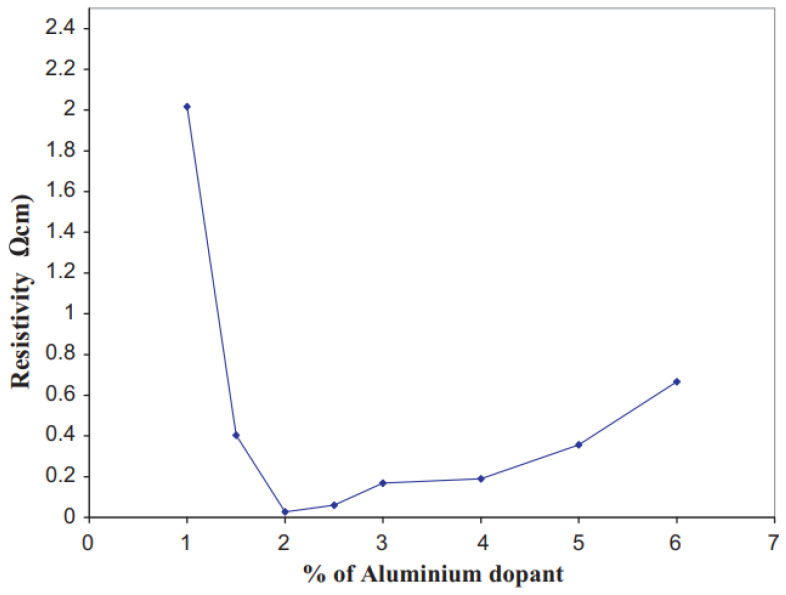
Resistivity for ZnO: Al films with increasing aluminum doping concentration. Reproduced from [216] under permissions from copyright clearance center.

The resistivity against % of Al dopant in the study [216] is plotted in Figure 60. A minimum-centered, almost parabolic behavior is noted. At 6 at. % doping, the resistivity began to rise with the doping concentration and reached 6.67 × 10^−1^ Ω^−^cm. Figure 60 also demonstrates that, at 2 at. % Al doping, the resistivity achieves a minimum. This result for sprayed ZnO: Al films at a similar substrate temperature and doping range is identical to prior publications [410,411]. Al was ionized into Al^3+^ and substituted for Zn^2+^ when a tiny quantity of Al was added to the ZnO layer. As a result, one zinc atom was exchanged for one aluminum atom to produce one free electron. The conductivity rises due to the enormous number of free electrons that little amounts of aluminum bring into the doped films. On the other hand, conductivity does not increase with additional increases in aluminum concentration (>2 at. %). Due to its low solubility, excess aluminum cannot be incorporated into the ZnO lattice; instead, it forms neutral aluminum oxide and separates at the grain boundaries [410]. As a result, when Al doping is high, there are fewer electrically active Al atoms in the film, and too much dopant causes carrier traps to form in the lattice, which lowers the mobility of the Al atoms.

According to a study [323], surface leakage reduces the accuracy, impacts, and other characteristics of a high-resistive film’s resistivity values. The relative electrical characteristics of several films are anticipated to be significant since the errors resulting from measurement uncertainty are thought to be systematic.

##### Effect of Temperature

**Figure 61:**
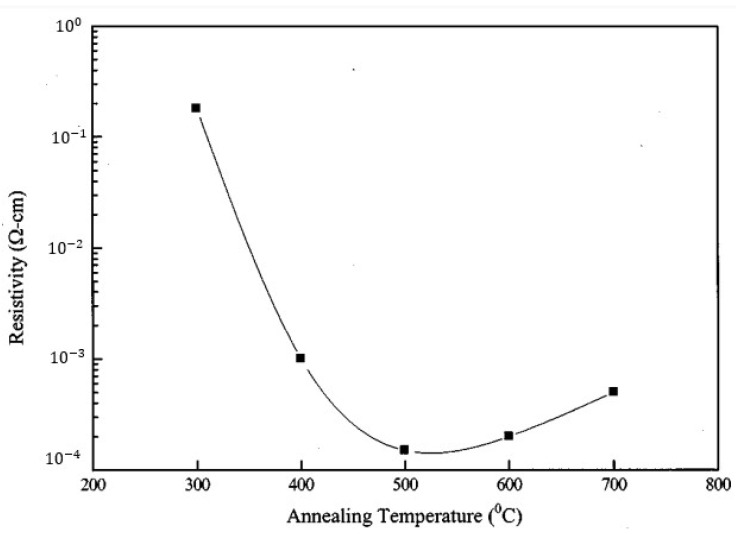
The relationship between the annealing temperature and the resistivity of AZO thin films. Reproduced from [214] under permissions from copyright clearance center.

Alam et al. [214] reported the variations in the resistivity of thin films of zinc oxide doped with aluminum, where the solution’s aluminum dopant concentrations underwent annealing at 500 °C in air, as shown in Figure 61. From Figure 61, we can see that the resistivity decreased with increasing temperature up to 500 °C. After that, the resistivity began to increase.

According to another study, the creation of oxygen vacancies is thought to cause the resistivity drop at annealing temperatures as high as 500 °C. High resistivity is also a result of the low crystallinity of films that are annealed at low temperatures. It is also demonstrated that crystallinity increases with increasing temperature. Similar to the phenomenon observed here, Ning et al. [213] demonstrated that the films’ resistivity rose and their crystallinity decreased when annealed beyond 500 °C. This could be connected to the decline in carrier mobility caused by an increase in grain boundary scattering for free electrons [411].

The growth temperature also impacts the films’ mobility and carrier concentration. It has been noted that, when the growing temperature rises, the carrier concentration and the mobility of the films first rise and subsequently fall. A decrease in zinc interstitials or oxygen vacancies at higher temperatures results in a more stable material with high resistivity. Furthermore, at high substrate temperature regimes, the effects of alkaline impurity out-diffusion from the substrate are enhanced, offsetting the donor nature of the impurities [408].

Yanfeng et al. [412] reported that oxygen atoms usually form Na zeolite structures, which might lead to the creation of more neutral impurity scattering centers in the low- and high-temperature ranges, aside from the middle one, thus increasing the influence of scattering. Hall mobility and carrier concentration consequently drop. When the amount of Al doping is high, there is a decrease in the concentration of electrically active Al in the film. This outcome is also a result of the solubility of aluminum in the ZnO lattice, which is restricted to a specific value (in this case, 3 at. %). Above this value, excess aluminum is thought to be segregated at the grain boundaries, raising the barrier height and lowering electron mobility and carrier concentration [273,412].

##### Effect of Deposition Techniques

**Figure 62:**
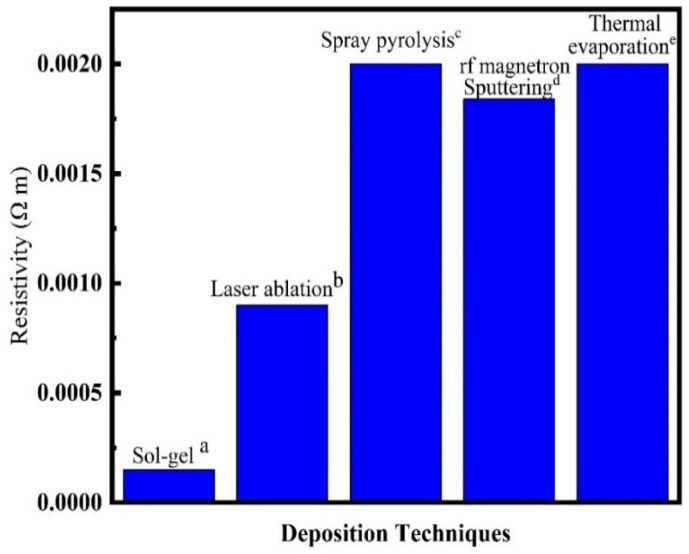
Resistivity of ZnO: Al films for different deposition techniques.

Alam et al. [214] revealed that the sol–gel approach yielded a minimum resistivity value of 1.5 × 10^−4^ Ω cm at 0.8. % aluminum concentration, which is better than values achieved by other traditional methods. The differences in the resistivity of ZnO films with aluminum-dopant created using numerous methods are in the study. Using the data from the study [214], Figure 62 was plotted to address the variations in the resistivity for different deposition techniques. The figure’s lowercase letters relate to certain research or references that provide resistivity values for the corresponding deposition methods. The lowercase letters (a–e) stand for references to the following particular studies: RF magnetron sputtering (d), spray pyrolysis (c), laser ablation (b), thermal evaporation (e), and sol-gel (a). Compared to films created using various methods, the optical transparency of Al-doped ZnO thin films created using the sol–gel approach is sufficiently high to be used as transparent electrodes. According to the study analysis, the sol–gel films perform significantly better.

##### Effect of Film Thickness

As film thickness increases, the resistivity of sprayed ZnO thin films decreases [413]. Greater film thickness promotes improved film stacking, which raises grain size and reduces grain defects and grain boundary scattering [414], both detrimental to carrier mobility. In the study [216], films were kept thinner than 1 mm to trade off transmittance, which decreases with thickness. Therefore, it is possible that the resistivity was higher than the current minimum values documented in the literature.

#### 5.1.2. ITO Thin Film

##### Effect of Film Thickness

According to research [278], conductivity increases as grain size and grain boundary decrease, reducing resistivity. Similar studies suggested that the resistivity gradually reduced as the thin-film thickness increased [405]. Quantum size effects become important in thin films, particularly in those with thicknesses close to or less than the characteristic length scales of the material. The confinement of charge carriers within the film as its thickness decreases may result in quantum confinement effects that change the electronic band structure and impact the material’s resistivity. Due to quantum size effects, thinning the film occasionally causes an increase in resistance [415].

In contrast with thicker films, thinner films could show variations in carrier concentration and mobility. Reducing the thickness of the film, for instance, may alter the growth mechanism and cause fluctuations in the film’s dopant concentration or carrier mobility [416]. These modifications may impact the film’s resistivity. The resistivity of the ITO thin film can also be affected by the substrate on which it is deposited. Thinner films might be more vulnerable to substrate effects, which could alter the film’s resistivity. These effects could include strain-induced alterations in the film structure or interdiffusion between the film and substrate materials.

##### Effect of Power and Deposition Time

Using the four-point probe, the electrical resistivity of the films produced with varying RF powers and deposition durations is determined. The following expression mentioned in Equation (24) is employed:

(24) $$\rho =\frac{2\pi d}{ln[(S_{1}+S_{2})(S_{2}+{\;S}_{3})/S_{1}S_{3}}\frac{V}{I}$$

where *S*_1_, *S*_2_, and *S*_3_ are the distances between the probes, and ρ, *I*, and *V* are the resistivity, current, and voltage, respectively.

A study [325] showed that the resistivity gradually decreases to a modest reduction, corresponding to a power of 50 W. Furthermore, as a function of power, the rise in carrier concentration causes the resistivity to grow [417]. Conversely, the evident redshift, or decrease in the bandgap, is the source of the resistivity decrease. Numerous atoms with multiple orbital overlaps comprise a large crystallite, which lowers the optical bandgap and thus results in low resistivity. It is evident that, as electron density rises, the energy gap shrinks. Similar electrical experiments showed that the resistivity falls when the RF power and the deposition time increase.

##### Effect of Temperature

A study [324] shows that the resistance decreases as the substrate temperatures increase. This is because an increase in substrate temperature causes increases in grain size, crystallization, and carrier concentration. As a result of the rise in temperature, resistivity values may also decrease positively due to the shift in the preferred crystallization orientation.

##### Effect of Grain Size

Scherrer’s formulation validates the vital link between resistivity and grain size. According to the computations in [324], there is a link between resistivity and grain size [418], and the resistivity of the DC-magnetron-sputtering-generated films was inversely proportional to the grain sizes. However, The RF-magnetron-sputtering-developed samples showed a very small correlation compared to the correlation observed between grain sizes and resistivities at higher substrate temperatures for the DCMS films. For DCMS- and RFMS-generated samples, the resistivities decreased by 4.5 and 3 times, respectively, with increased substrate temperature. Another study [419] showed that the resistivity decreased gradually with the increasing grain size.

##### Effect on Bandgap

There is a relationship between resistivity and bandgap; as substrate temperature rises, the bandgap increases, and resistivity falls. There is a relationship between the rise in carrier concentration and bandgap at higher substrate temperatures. The Burstein–Moss effect can well describe ITO films’ widening bandgap shift [420].

##### Effect of Sputtering Voltage

The sputtering voltage strongly influences the film resistivity. A study [421] revealed that a lower collision energy of negative ions might decrease the resistivity of ITO films. They created the ITO films on fixed substrates by lowering the sputtering voltage to −110 V. Additionally, the study demonstrated the resistivity variations in ITO films generated at various sputtering voltages. The resistivity decreased at every substrate temperature in contrast to the sputtering voltage, and it did not increase right above the erosion area. However, when the sputtering voltage was adjusted to −370, −250, and −110 V at 200 °C substrate temperature, the resistivity decreased at all positions, and the rate of resistivity growth above the erosion area reduced with a lower sputtering voltage. The negative ion damage to the film diminished with decreased sputtering voltage. This suggests that a lower sputtering voltage can effectively decrease the ITO film resistivity [415].

##### Effect of Carrier Concentration

An increase in carrier concentration explains the decreased resistivity at lower sputtering voltage. In a study [325], only the carrier concentration rose while the sputtering voltages were decreased, and the mobility remained constant for all substrate temperatures. This suggests that negative ion collisions cause damage that mainly affects the concentration of charge carriers and does not affect mobility. The mechanism of carrier concentration drop by negative ion damage is caused by an increase in bivalent In or Sn as acceptors and a decrease in oxygen vacancies as donors. Sn^4+^ is typically the predominant donor in ITO film created at high temperatures, and oxygen partial pressure does not significantly influence the resistivity. Even in low-resistivity ITO films produced on high-temperature substrates, the carrier concentration is considerable, and the resistivity dependency on oxygen partial pressure is significant. This is due to the low sputtering voltage [422]. This suggests that there are more oxygen vacancies available to donate. When Ar ions splatter the ITO target, they produce a high-resistance black suboxide on the surface. A high sputtering voltage can produce the same reaction in the ITO film, which causes oxygen ions to collide negatively to form highly resistive black suboxide. This substance will increase the film’s resistivity [423].

#### 5.1.3. CuI Thin Film

##### Effect of Temperature

Kaushik et al. [56] reported a resistivity value of 72 mΩ m for CuI thin films at ambient temperature (300 K), which is less than previously published works by Inudo et al. [194] and Zhu and Zhao [190]. Inudo et al. [194] investigated CuI films, and, using the van der Pauw method, they examined the electrical resistivity. When the thin films were annealed in an Ar environment, they showed resistivities between 1 and 5 Ω cm. They also mentioned that, compared to films annealed in Ar at the same temperature, the films annealed in air at temperatures below 200 °C showed somewhat lower resistivities. At about 130 °C, the lowest resistivity (0.3 Ω cm) was achieved during the annealing process. But, when the annealing temperature rose, the resistivities of the films annealed in air tended to rise as well, becoming too high to detect at temperatures beyond 250 °C. The oxidation of CuI to generate CuO should have been the cause of this notable rise in resistivity. In another study reported by Zi et al. [359], a resistance of 10 mΩ cm was found for thermally evaporated CuI thin films produced at 120 °C substrate temperature. However, the electrical resistivity measurements at low temperatures shed more light on how charge carriers are transported through the material. Reports on low-temperature transport investigations of CuI thin films are quite rare. In a similar study reported by Zhu and Zhao [190], the resistivity rises to the order of 1 Ω cm as the substrate temperature rises to 350 °C. Additionally, grain size increases with substrate temperature, which is suitable for hole transport since it reduces grain boundary scattering. However, a notable drop in hole concentration causes a rise in resistance as substrate temperature rises.

**Figure 63:**
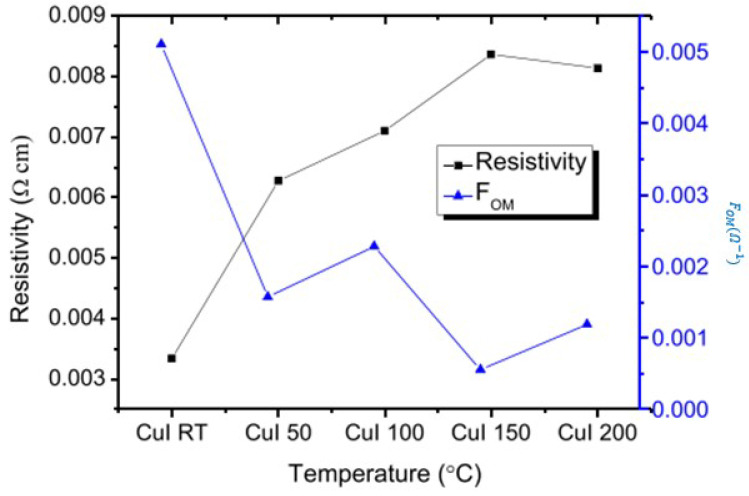
CuI thin films’ resistivity and figure of merit as a function of annealing temperature. Reproduced from [54] under permissions from copyright clearance center.

Another study [54] showed that the CuI thin-film resistivity increased with increased annealing temperature, as shown in Figure 63. It has been discovered that Cu vacancies (V_Cu_) are essential native acceptors in CuI thin films [424]. The increased carrier concentration at lower temperatures is the reason for the as-deposited film’s reduced resistivity [359]. Because of the decrease in V_Cu_, resistance rose when the annealing temperature was raised. The evaporation of iodine from CuI thin films at higher annealing temperatures is responsible for the reduction in V_Cu_ with increasing annealing temperature [199,362].

##### Effect of I/Cu Ratio

For PLD-prepared CuI thin films, Rusop et al. [425] observed that resistivity rose with the I/Cu ratio. Tanaka et al. [187] discovered comparable outcomes for sputtered CuI thin films. They both believed that Cu^+^ was the dominant carrier, which is why the low resistivity at a lower I/Cu ratio was observed. Actually, the γ-CuI is often Cu-deficient, and CuI becomes a p-type semiconductor because of the hole created by the defect equation, which can be understood by using Kroger–Vink defect notations.

Tennakone et al. [426] reported that γ-CuI thin films should achieve high conductivity at a high I/Cu ratio. When comparing the PLD-prepared CuI thin films by Rusop et al., the addition of oxygen causes a resistivity drop of the order of 10^4^ Ω cm. According to Herrick and Tevebaugh [427], adding oxygen to CuI films enhances their conductivity [424].

##### Effect of Doping Concentration

**Figure 64:**
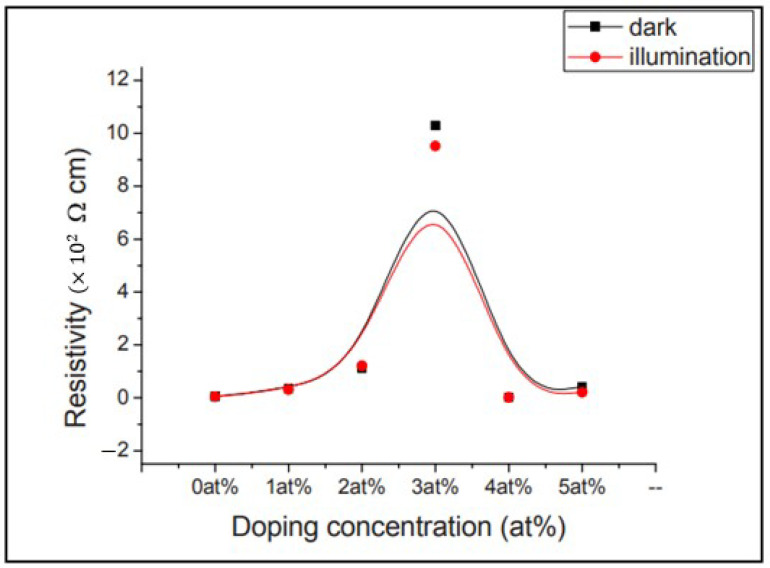
Variations in CuI film resistivity at varying iodine doping concentrations. Reproduced from [191] under permissions from copyright clearance center.

The doping process significantly impacts the electrical characteristics of semiconductor materials. Figure 64 presents the resistivity pattern for undoped and iodine-doped CuI films at 1 at. % to 5 at. % from the investigation by Amalina et al. [191]. It is evident that the resistivity rose for films manufactured with a 3 at. % doping concentration, reaching an optimal peak of 1.03 × 10^3^ Ω cm from 4.75 cm for undoped thin films. The excess iodine in the stoichiometric surplus determines the p-type conductivity of CuI. As a result, iodine doping can raise or lower the resistivity, which relates to where the iodine-induced surface traps are located in the CuI band position. Since the interstitial I_2_ atoms in the lattice operate as a charge trap for electrons, the resistivity likely increases as the concentration of I_2_ ions increases. In addition to stimulating recombination, the surface traps in the lattice caused by the excess iodine also lower carrier mobility. According to Perera et al., the surface trapping sites are located 0.2 eV above CuI’s valence band [428]. However, as shown by Amalina et al. [191], the maximum resistance is brought on by the thin-film cracking; the carrier scattering caused by the fracture coatings will result in reduced electron mobility. The reduced grain boundaries that result in improved carrier mobility under conditions of greater doping concentration could cause resistivity reductions for 4 at. % and 5 at. %.

However, several studies have been conducted on resistivity behavior with the doping concentration on CuI thin films. Using spray pyrolysis, Nesa et al. [429] reported high optical conductivity and lower electrical resistance for CuO thin films doped with Zn. Saied et al. [430] investigated the characteristics of Ti dopant, which improves the conductivity of the films while maintaining the samples’ optical qualities and crystallinity. As the Ni concentration of the CuO thin film increased, so did the optical bandgap and electrical resistance [431].

#### 5.1.4. Polymer Thin Films

A wide variety of electrical resistivity values can be observed in polymer thin films, contingent on several variables, including dopants, manufacturing circumstances, molecular structure, and type of polymer.

##### Insulating Behavior

Many polymers function as insulators and show significant electrical resistivity, particularly those with strongly branched or highly crosslinked molecular structures [432]. In these situations, the absence of mobile charge carriers causes the polymer thin films to block the flow of electrical current effectively.

##### Conducting Behavior

Some polymers have delocalized π-electrons along their backbone, providing them high electrical conductivity. These polymers are referred to as conducting polymers. Poly(3,4-ethylenedioxythiophene) (PEDOT), polyaniline, and polypyrrole are a few examples of conducting polymers. Conducting polymer thin films can have relatively low electrical resistivity values, which makes them appropriate for electrical-conduction-dependent applications, including electrochromic devices, flexible electrodes, and sensors [369,433].

##### Semiconducting Behavior

Some polymers can behave as semiconductors, especially conjugated polymers or polymers doped with certain chemicals. Under some circumstances, these polymers may have intermediate electrical resistance values, allowing them to conduct electricity to some degree [302]. Organic photovoltaics, organic field-effect transistors (OFETs), and OLEDs are interesting applications of semiconducting polymer thin films [115].

Additional variables that might affect the electrical resistivity of polymer thin films include processing method, processing level, degree of crystallinity, film thickness, and ambient factors (such as humidity and temperature) [434]. Comprehending and managing these variables are vital in customizing the electrical characteristics of polymer thin films for distinct uses. In conclusion, depending on their composition, structure, and production, polymer thin films can display various electrical resistivity values, ranging from insulating to semiconducting to conducting behavior. Polymer thin films are interesting for a wide range of applications in electronics, energy, sensing, and optoelectronics due to their diverse electrical properties.

#### 5.1.5. INO Thin Film

##### Effect of Oxygen Pressure

Investigations were conducted into the DC electrical resistivity of INO films created at various substrate temperatures by Beena et al. [342]. The INO films exhibit relatively high room temperature DC electrical resistivity values, ranging from 0.80 to 1.90 Ω m. For INO films created in an oxygen-free atmosphere, Yamada et al. obtained [435] a low DC electrical resistivity value of 1.53 × 10^−5^ Ω m. A still lower DC electrical resistivity value (1.7 × 10^−6^) Ω m for INO films created at an oxygen pressure of 10^−3^ mbar was reported by Gupta et al. [436]. The INO films lack stoichiometry due to the existence of oxygen-array vacancies, which can function as doubly ionized donors and provide two electrons for electric conduction. In ref. [372], the films were prepared at 0.02 mbar, a somewhat more tremendous oxygen pressure. The films produced in this manner can be nearly stoichiometric, as indicated by their high optical transparency and improved stoichiometric character, which leads to a comparatively higher DC electrical resistivity analysis value. These films have incredibly few oxygen shortages.

##### Effect of Temperature

**Figure 65:**
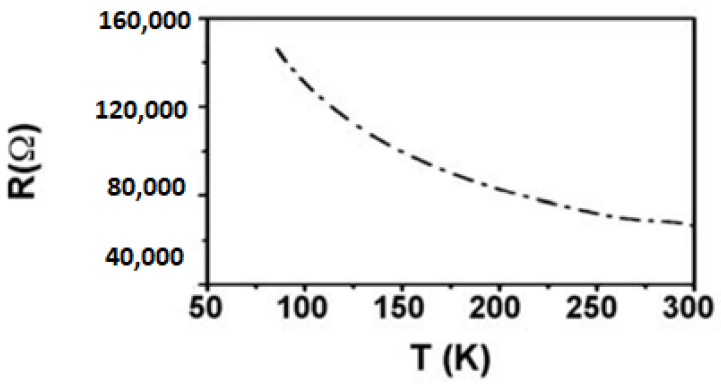
Changes in the electrical resistivity with increasing temperature. Reproduced from [342] under permissions from copyright clearance center.

Figure 65 illustrates the study of the temperature dependence of electrical resistance of INO films formed at an oxygen pressure of 0.02 mbar across a temperature range of 50–300 K [342]. The film is semiconducting in nature, as evidenced by its negative temperature coefficient of resistance.

### 5.2. Electrical Conductivity

The ability of a material to conduct electricity in response to various light wavelengths or frequencies is known as the optical conductivity of thin films. It measures a material’s ability to conduct electricity when exposed to light. This feature is essential in optoelectronics, where materials are used for their capacity to conduct electricity and interact with light [437]. The relationship between electronic resistivity (ρ) and electronic conductivity (σ) is inverse. The link between resistivity and conductivity can be represented mathematically as follows:

σ = 1/ρ (25)

Measurements of optical conductivity offer crucial new information about the charge transport characteristics and electrical structure of thin films. Researchers use these measurements to examine the material’s band structure, carrier density, mobility, and scattering mechanisms. Understanding optical conductivity makes designing and improving thin-film electronic and optoelectronic devices, such as solar cells, photodetectors, LEDs, and transparent conductive coatings, more accessible [438]. Higher optical conductivity, for example, can result in more effective light absorption and current generation in solar cells. For applications like touchscreens, flat-panel displays, and OLEDs that require clear conductive coatings, thin films with excellent optical conductivity and transparency are crucial [437,438] Determining and managing these materials’ optical conductivity are essential to attaining peak device performance.

#### 5.2.1. Ti-Doped Al_2_O_3_ Thin Film

Aluminum titanium oxide (Al-Ti-O) is a material that exhibits enhanced electrical conductivity due to titanium doping in Al_2_O_3_ thin films. This process creates oxygen vacancies and Ti^3+^ states, which function as electron donors. TCOs are frequently created using this doping technique as transparent electrodes in touchscreens, solar cells, and displays, among other uses. Al_2_O_3_’s band structure is changed by titanium doping, preserving optical transparency while improving electrical transport characteristics. Research revealing this behavior was conducted by Yang et al. [439]. They concluded that substitutional Ti^3+^ ions induce impurity band states and form Ti^3+^ clusters in Al_2_O_3_ GBs and that Ti doping can control the electrical band structures of GBs. It should be noted that the segregation of Ti^3+^ clusters in the GBs may also account for the enhanced conductivity in Ti-doped Al_2_O_3_, as reported previously [440], since the third electron of Ti^3+^ is available for electronic conduction, as demonstrated in Ti-doped Al_2_O_3_ dislocations [441,442]. Ultimately, Yang et al. [439] discovered that Ti^3+^ ions at the GBs introduce an extra impurity band in the bandgap, which may be the cause of the Ti-doped polycrystalline Al_2_O_3_’s improved conductivity through the GBs.

#### 5.2.2. CdO Thin Film

Without any intrinsic doping, CdO film is an n-type semiconductor with high electrical conductivity. Undoped CdO thin films have high electrical conductivity because of oxygen vacancies and Cd interstitials [443].

##### Effect of Doping

**Figure 66:**
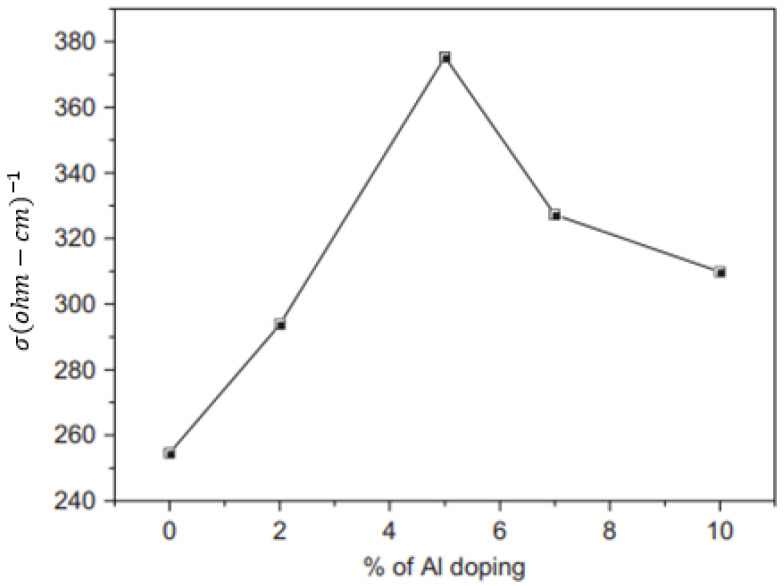
Variations in electrical conductivity with doping concentration. Reproduced from [377] under permissions from copyright clearance center.

Variations in electrical conductivity have been noted with aluminum doping. A study [377] indicated these variations, as presented in Figure 66. Initially, it was discovered that conductivity increased as aluminum concentration increased, but doping concentration ranged from 1 to 5%. It is found that conductivity decreases as the doping percentage of Al increases. In their earlier work, they observed a comparable fluctuation in the DC conductivity of Al: CdO films created using the sol–gel process [444]. The following explanation applies to the observed increase in electrical conductivity caused by aluminum doping: aluminum contains one extra valence electron compared to cadmium, so, when aluminum is doped, the concentration of free charge carriers in CdO rises. Aluminum may be considered to replace the cadmium atom or occupy the interstitial sites. In both situations, the donor material is aluminum. However, excessive Al doping may break down the crystalline structure. At greater Al concentrations, the conductivity in CdO: Al thin films decreases due to an increase in free carrier absorption. Once more, a minor amount of Al_2_O_3_ formed at a greater doping level may also decrease conductivity [377].

##### Effect of Deposition Technique

An investigation was conducted by Saha et al. [377] on different deposition techniques and their impact on the thin film produced. When comparing the RF sputtering process to their previous work [444] on the sol–gel method of producing Al-doped CdO thin films, there are a few benefits. These include generally higher film quality and the ability to fabricate device-quality films using RF sputtering. Using the RF sputtering approach, one may manage more aspects of the deposition process, such as substrate temperature, RF power, gas partial pressures, etc. Conversely, the sol–gel method is less complicated and more affordable.

Table 11 shows the overview of different factors that impacted the resistivity and conductivity of different conductive thin films through other authors’ reports.

## 6. Recent Trends of Conductive Thin Films

Novel materials such as 2D materials (graphene and transition-metal dichalcogenides), perovskites, organic semiconductors, and quantum dots are being investigated by researchers for use in thin-film applications [447]. These materials present new opportunities for thin-film applications in photonics, electronics, and energy conversion because of their unique qualities and capabilities, including tunability, flexibility, and quantum confinement effects. The development of thin-film technologies is becoming increasingly popular for wearable, conformal, flexible, and elastic electronics. Applications for thin-film materials with mechanical flexibility and stretchability, like metal oxides and polymers, are being researched for electronic skins, sensors, flexible displays, and health monitoring systems [448]. These patterns show the continuous attempts to develop thin-film science and technology and take advantage of thin films’ unique qualities for various uses. Thin-film research and development are motivated by the need for novel materials and manufacturing techniques to solve the present problems and pave the way for upcoming technological advances.

Furthermore, the search for environmentally responsible and sustainable solutions has prompted researchers to investigate conductive polymers and organic semiconductors as substitutes for conventional inorganic materials. These thin films composed of organic materials have benefits, including being lightweight, low production costs, and compatibility with flexible substrates, making them perfect for energy harvesting devices, flexible displays, and wearable electronics. Moreover, the large-scale manufacturing of conductive thin films is now possible because of developments in deposition techniques such as atomic layer deposition, roll-to-roll processing, and inkjet printing. This opens the door for the widespread use of conductive thin films in next-generation electronic devices.

### 6.1. Graphene

**Figure 67:**
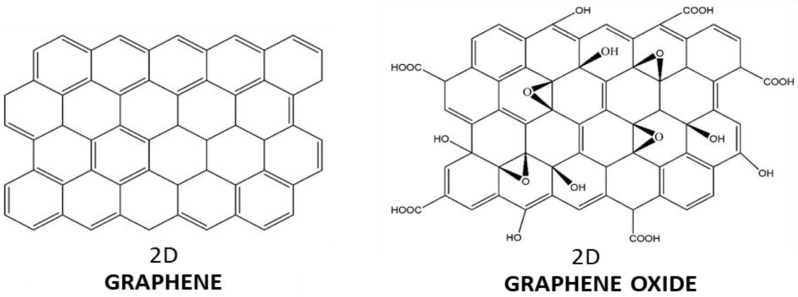
Structure of graphene. Reproduced from [449] under permissions from copyright clearance center.

Smart 2D carbon material graphene (GR) is a promising option for various energy storage applications due to its unique properties, including high specific surface area, high electrical and thermal conductivity, tunable bandgap, and superior electrochemical stability [447]. Figure 67 shows the schematic representation of the 2D graphene structure [449].

**Figure 68:**
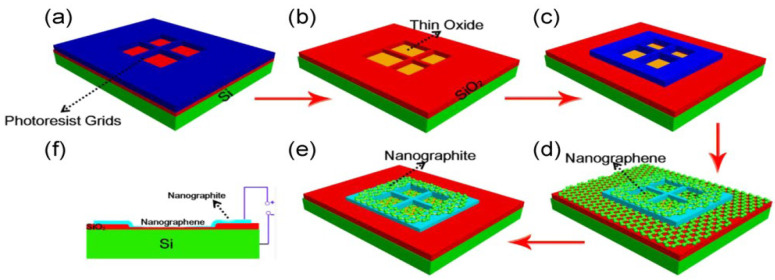
Diagram illustrating the fabrication technique of nanographene-oxide–silicon photodetectors. (**a**) Photolithography is used to selectively remove a 3 × 3 mm^2^ patch of photoresist from the wafer’s core. (**b**) The photoresist acts as protection as the exposed SiO_2_ layer is entirely removed using buffered oxide etchant. The exposed silicon develops a thin oxide layer once the photoresist is removed. (**c**) On the Si/SiO_2_ arrangement, a photoresist structure is properly aligned. (**d**) The structure is heated to 1000 °C in a vacuum quartz tube for 10 min while being shielded from a 100 sccm 5% H_2_/Ar gas flow. The photoresist grids are converted into nanographite by this heat treatment, and nanographene is also developed on the cleaned SiO_2_ and etched silicon. (**e**) Oxygen plasma is used to etch nanographene around the periphery to avoid possible interaction with the adjacent silicon. (**f**) A schematic cross-sectional view of the apparatus shows the parts and assembly. Reproduced from [450] under permissions from copyright clearance center.

Graphite materials composed of tiny crystallites, usually on the order of nanometers in size, are referred to as nanocrystalline graphite (NCG). The nanoscale structure of these materials gives rise to special characteristics like large surface area, tunable electrical properties, and mechanical flexibility. The prospective uses of NCG in a variety of industries, such as electronics, energy storage, catalysis, and sensing, have attracted a great deal of interest. In order to modify the crystallites’ size, shape, and orientation and customize their properties for particular uses, a variety of procedures are used during their synthesis. Many deposition methods, such as molecular beam epitaxy, radio-frequency magnetron sputtering, atmospheric pressure plasma deposition, pulsed laser deposition, and chemical vapor deposition, are used in the production of NCG thin films. Furthermore, techniques such as organic monolayer conversion and high-temperature pyrolysis of molecular precursors have been applied, as shown in Figure 68.

**Figure 69:**
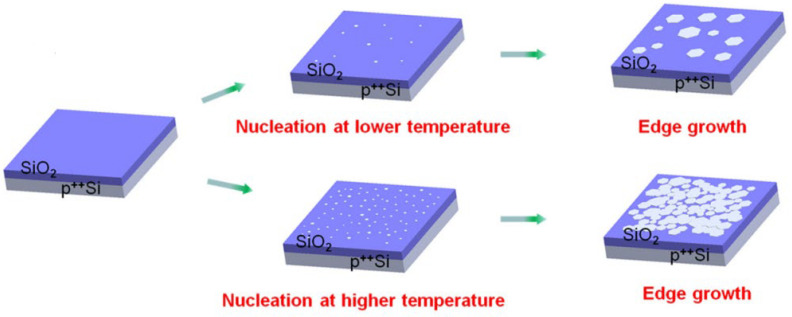
Two-step development of nanographene on SiO_2_. Reproduced from [452] under permissions from copyright clearance center.

These methods produce thin films that can be used in a variety of contexts, including the creation of devices. High-quality NCG thin films are produced by the expensive vacuum atmosphere sublimation of 6H SiC, as described by Chandrashekhar et al. [451]. The review emphasizes the importance of CVD synthesis, which uses metal catalysts to generate graphene and then transfers it onto substrates for electrical applications. All things considered, the various techniques covered provide opportunities for customized NCG film manufacturing, with CVD being essential to the synthesis of graphene. In another study, Lui et al. [452] developed a catalyst-free, direct-growth method for nanographene using chemical vapor deposition with distant plasma assistance on a variety of substrates. Figure 69 illustrates the two steps in nanographene development on SiO_2_.

It is necessary and demanded that various applications design and synthesize intelligent, economical, and efficient multicomponent nanocomposite thin films based on diverse dimension nanomaterials using an environmentally friendly process. As a result, numerous studies based on zero-, one-, and two-dimensional nanomaterials have been conducted to create novel and environmentally friendly synthesis methods for new 2D network thin films. Some related studies are mentioned below.

1. Nour et al. [453] investigated using an in-pot approach; polyaniline nanofibers (PANI-NFs) were directly produced on a glass substrate with GR sheets exfoliated from graphite in an aqueous solution. Titanium dioxide nanoparticles (TiO_2_NPs) were added for decoration, resulting in a smart 2D nanocomposite film called PANI-NF-GR-TiO_2_ NP. The intriguing 2D structure network thin-film device for various applications is then adorned with a thin layer of polypyrrole (PPY) using vapor phase polymerization. The study also showed that the novel 2D thin film has several active sites and an intriguing device structure for various applications. It comprises four layers of one-dimensional (1D) polyaniline nanofibers, 2D graphene adorned with zero-dimensional (0D) nanoparticles, and a thin polypyrrole layer.
2. With its distinct structural, physical, and electrical characteristics, graphene is predicted to favor the advancement of thin-film solar cells. The study [454] determined if graphene may produce solar cells as an active interfacial layer and front and back connections. The study found that graphene has been demonstrated to be a good substitute for the TCO layer in the CdTe and CIGS systems due to its exceptionally high carrier mobility, transparency, and appropriate work function. While the back contacts of graphene should be sufficiently thick to avoid additional current leakage, the front contacts of the material could have a monolayer structure. The experiment revealed that the graphene layer requires more carrier transportation, complete surface coverage, and higher sheet resistance in a lateral orientation. Even though graphene outperformed the traditional hole transport layer in the long-term stability test, the device parameter loss must be corrected before commercialization.
3. According to the role of graphene, which acts as an electrode material and a channel layer in FET, the recent development of flexible thin-film transistors based on graphene and graphene/semiconductor heterostructures was demonstrated in the study conducted by Zhongcheng et al. [455]. The study also stated that, although graphene still has several technological challenges to be solved, it appears to hold great promise for flexible electronic applications that are challenging to implement with the traditional materials. The large-scale integration of two-dimensional materials, particularly graphene, with materials of different dimensionalities is anticipated to significantly influence semiconductor technology in the future. Moreover, it is crucial to have single-crystalline films with consistent thickness and few voids, wrinkles, ripples, and other impurities. As a result, the direct synthesis of superior 2D materials on insulating substrates ought to be paid top attention. Overall, the enormous mixed-dimensional integration opportunities point to significant future growth potential for this sector in applied technology and basic research.
4. According to studies [6,373,455], further advancements in heterostructure synthesis, 2D material growth, and device fabrication will result in future practical applications.
5. In a study [456], two distinct graphene strategies were presented for engineering strain in graphene. These strategies used the shrinkage feature of thin films deposited on graphene in a planar shape, eliminating the requirement to bend the substrate. In a controlled manner, graphene was found to exhibit both biaxially strained states and isotropic compressive strained states. The techniques presented here promise to advance graphene strain engineering and investigate novel physics in strained graphene. Furthermore, this work broadly applies to other two-dimensional crystals, including atomically thin metal dichalcogenides.
6. According to the investigation of Shuaishuai et al. [457], graphene films with a regulated number of layers or superior monolayer graphene films have been found to grow when thin-metal films are used as the substrate. Cu (111) and CuNi (111), in particular, are single-crystal metal films and alloys that have also been reported and studied as substrates for the reported formation of single-crystal graphene. Single-crystal films with a regulated number of layers might be achievable by expanding these methods to produce other single-crystal metal or alloy films. Further investigation into binary alloys and their extension to ternary (and other) alloy films appears to have ample potential as substrates for forming n-layer graphene films, enabling the exact control of n-layers.
7. Owing primarily to its physical and chemical characteristics, graphene oxide (GO) is a versatile material that has been making waves in the biomedical industry in recent years. As mentioned throughout this study [458], many applications can benefit from incorporating GO. These applications include those that use it as a carrier given the large amount of drug it can encapsulate and transport; as a structural component that keeps the drug delivery system (DDS) intact until it reaches its target, preventing the leakage of bioactive compounds; or as a capping layer or barrier that enables the DDS to be fine-tuned and encourages the release of biomolecules in a timely and sequential manner. Furthermore, new and interesting uses of GO are being researched daily, even though its potential for medicinal applications still requires further evaluation.

#### 6.1.1. Single-Layer Graphene

A single sheet of carbon atoms organized in a two-dimensional honeycomb lattice is referred to as single-layer graphene [459]. Its extraordinary qualities, such as great mechanical strength, flexibility, and electrical conductivity, make it significant. Because of these qualities, it is a very sought-after material for many applications, such as electronics, energy storage, sensors, and more. Conductive thin films are important for single-layer graphene use [460]. Solar cells, flexible electronics, touchscreens, and other electrical devices all use these films. Next-generation electronics with increased performance and flexibility can be developed by depositing single-layer graphene onto substrates to create transparent, highly conductive thin films [461].

**Figure 70:**
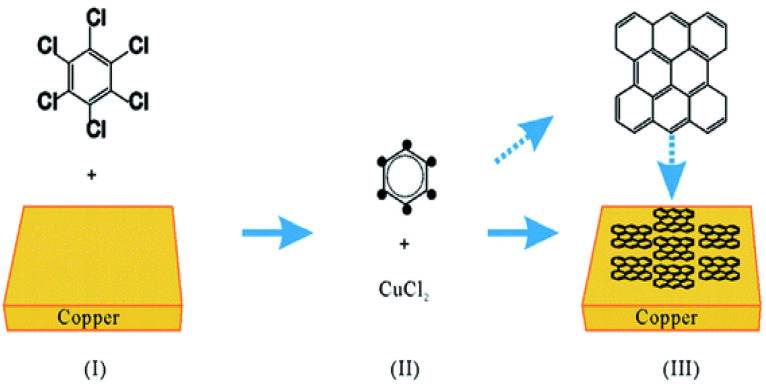
Process flow diagram for the generation of graphene flakes. (I) The first phase of copper’s exposure to hexachlorobenzene, (II) when CuCl₂ is present as a catalyst, the benzene derivative and copper complex form, (III) the last phase of the complex’s decomposition, during which graphene layers start to develop on the copper substrate. Reproduced from [466] under permissions from copyright clearance center.

There are several recognized techniques for synthesizing single-layer graphene, the most widely utilized being CVD, chemical synthesis, and mechanical exfoliation. Among them, CVD is the most promising method for generating large-area, high-quality single-layer graphene [462]. Researchers have worked to improve CVD over time using a variety of precursors in solid, liquid, and gaseous forms [463]. The potential of CVD for graphene production was first shown by early experiments, such as the breakdown of camphor on nickel foil, albeit there were difficulties in minimizing folding and reducing the number of graphene layers. Different solid carbon precursors, including PMMA, sucrose, fluorene, and polystyrene, were investigated in later research [464]. Each of these precursors had a distinct benefit in terms of the synthesis temperature and the quality of graphene that was created. Notably, high-quality single-layer graphene can now be produced at much lower temperatures due to updated CVD techniques that use solid carbon sources like hexachlorobenzene (HCB) on copper foils, as shown in Figure 70 [465]. This increases the adaptability and efficiency of the graphene synthesis procedures.

**Figure 71:**
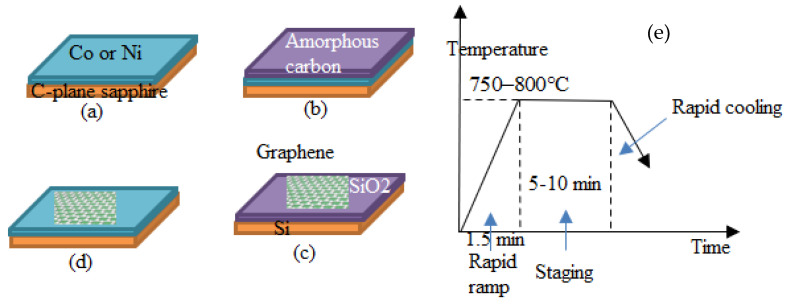
A schematic representation of the general developing graphene process. (**a**) To create a crystalline metal substrate, a thin metal coating (about 200 nm) of either Co or Ni is first sputtered onto a c-plane sapphire substrate at a high temperature. (**b**) A thin coating of amorphous carbon (a-C) is then sputtered onto the metal sheet after the substrate has been cooled to ambient temperature. (**c**) After the vacuum pressure reaches around ∼3.0 × 10^−4^ Pa, the annealing process starts. There are three essential steps in this: (1) the a-C/metal/sapphire structure is heated quickly to an annealing temperature of 750–800 °C for 1.5 min. (2) The substrate is then kept at this temperature for 5–10 min to allow the a-C to dissolve into the metal film. (3) Finally, the substrate is carefully cooled to room temperature. (**d**) Graphene forms on the metal’s surface after annealing. (**e**) In order to conduct additional analysis, the graphene layer is finally moved to SiO_2_/Si substrates.

Alternative methods have been found for the production of monolayer graphene films in addition to the CVD method. Orofeo et al. [467] presented a method for annealing amorphous carbon placed onto cobalt (Co)/sapphire and nickel (Ni)/sapphire substrates in order to produce large homogenous monolayer graphene films. The procedure, which is shown in Figure 71, entails sputtering Co and Ni metal films onto sapphire substrates. It was discovered that this is essential for improving the metals’ crystallinity and making single-layer graphene easier to generate. The production of monolayer graphene is facilitated by highly crystalline substrates that have fewer grain boundaries. Furthermore, unlike the traditional synthesis methods utilizing CVD and polycrystalline metal substrates, Cheun Lee et al. [465] demonstrated that the cooling rate during annealing did not alter the homogeneity of the monolayer graphene layer. This study broadens the range of graphene production methods by highlighting an alternate route to produce high-quality monolayer graphene sheets. This procedure offers an efficient way to create high-quality monolayer graphene films and provides information on the critical growth and transfer stages onto various substrates for further characterization.

**Figure 72:**
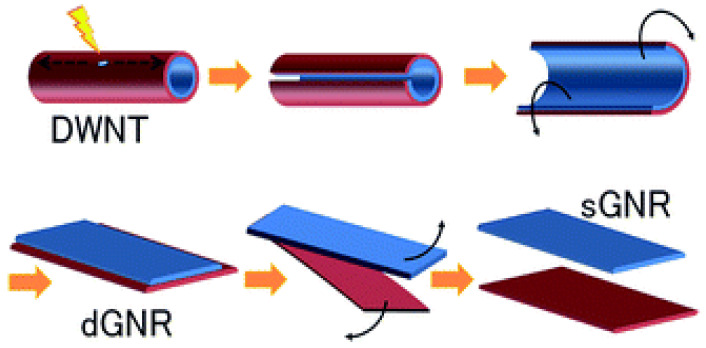
Synthesis of sGNR using DWCNTs. Reproduced from [468] under permissions from copyright clearance center.

Since each single-walled carbon nanotube (SWCNT) is effectively a rolled-up graphene layer, unzipping SWCNTs provides a method for producing graphene [465]. Graphene nanoribbons (GNRs), or thin, elongated graphene strips, are the result of this unzipping process. Tanaka et al. [468] have found that double-walled carbon nanotubes (DWCNTs) are a more effective precursor material than multi-walled carbon nanotubes (MWNTs) for the successful fabrication of single-layer graphene nanoribbons (sGNRs). The method of unzipping DWCNTs to create sGNRs is shown in Figure 72. The DWCNTs were first annealed in air at 500 °C to produce flaws, and then they were dispersed in an organic solution. A subsequent sonication treatment made the unzipping of dispersed DWCNTs into superior double-layer (d)-GNRs easier. These GNRs were subsequently further sonicated to generate individual sGNRs. These sGNRs mark a major breakthrough in the field of graphene-based electronics.

**Figure 73:**
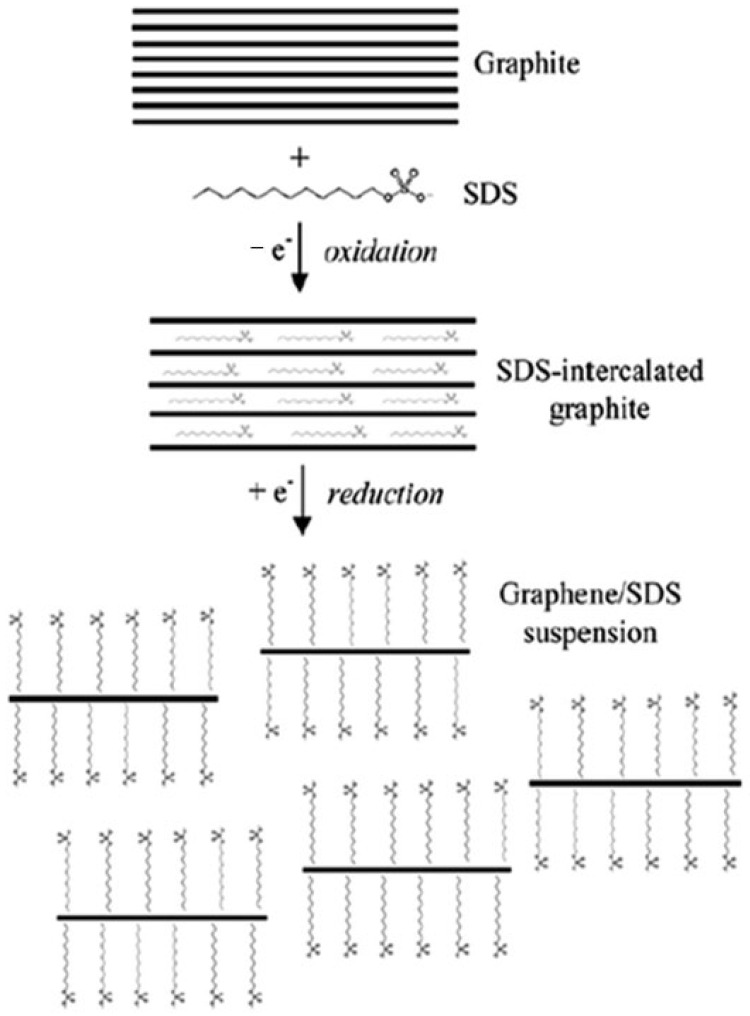
Diagram illustrating the electrochemical process used to generate a graphene/SDS solution. Reproduced from [469] under permissions from copyright clearance center.

An interesting electrochemical method for producing graphene films at monolayer thickness was presented in a study [469]. Sodium dodecyl sulfate (SDS) was electrochemically intercalated into graphite in this technique, and then the SDS-intercalated graphite was electrochemically exfoliated. As shown in Figure 73, the electrode potential during SDS intercalation had a substantial effect on the properties of the resultant graphene sheets, including the number of graphene layers, dimensions, and structural order. Interestingly, substantial intercalation potentials were needed to produce monolayer graphene sheets, and intercalation only happened at electrode potentials higher than 1.4 V [465]. Due to surfactant adsorption on the graphene film surface, the intercalation method prevented individual graphene sheets from restacking in the solution, giving it an edge over the conventional exfoliation techniques. As a result, this method offers a promising way to produce vast amounts of structured graphene films for a variety of uses.

The prospects are promising for conductive thin films and single-layer graphene in the future. Researchers are still investigating new uses and production methods to fully utilize graphene’s special qualities. The following are a few possible future trends:

1. Flexible Electronics: Graphene’s distinct mechanical and electrical characteristics, coupled with its high optical transmittance, have made it a potential material for flexible transparent conducting electrodes. Additionally, because of its adaptability, graphene is perfect for wearable and flexible electronics, like displays and sensors. A study by Han et al. [470] presented flexible electronic devices on plastic substrates as a promising technology for solar cells and displays in the future and called for graphene to take the place of the expensive and fragile ITO electrode. Because of its special qualities, graphene is a great option for transparent conducting electrodes in flexible electronics, but, before it can be used in real applications, some issues need to be resolved. The review examined the use of graphene in solar cells, FETs, and LEDs. It presented solutions to address its drawbacks and create flexible, extremely stable electronic devices. It also outlined prospective avenues for future study in this area.
2. Energy Storage: As single-layer graphene has high surface area and electrical conductivity, it has the potential to be used in batteries, supercapacitors, and other energy storage devices. Pomerantseva et al. [471] emphasized in their research how single-layer graphene, in particular, has the potential to revolutionize energy storage technologies. Electrodes based on nanomaterials have enhanced electronic conductivity and ionic transport, resulting in rapid ion diffusion and high specific capacities. Because these electrodes can tolerate high currents, they present a viable option for high-power and high-energy storage. Despite the current obstacles, a wide range of nanomaterials, from oxides to carbon-based structures, are available. These materials can be used to create novel energy storage solutions, such as wearable and structural energy storage technologies, which were previously impractical to achieve with the conventional materials.
3. Sensors: Graphene’s sensitivity to environmental changes allows it to be used in a variety of sensing applications, such as gas detection, biological sensing, and environmental monitoring. Tung et al. [472] emphasized the necessity for sensors with qualities like low cost, high selectivity, and quick response times, pointing out the growing interest in next-generation sensor devices across a range of industries in the study. Because of their remarkable qualities, graphene and its derivatives are positioned as the perfect materials to satisfy these needs. The research investigated the latest developments in the synthesis of graphene-based superstructures in a range of forms and dimensions, such as fibers, thin films, foams, and aerogels. These materials’ excellent sensing capabilities and adaptability are demonstrated by the variety of applications they are used in, including piezoresistive sensors, gas sensors, and biological sensors.
4. Composite Materials: Adding graphene to composite materials can improve their mechanical, electrical, and thermal qualities. This can result in the creation of high-performance, lightweight materials for the building, automobile, and aerospace industries. The state of composite materials containing graphene and carbon nanotubes, long viewed as promising developments in nanotechnology applications, has been critically examined by different authors [471,472]. There are still unanswered practical questions about the efficacy of these composites after almost 20 years of research. Their broad use is hampered by problems such as poor load transfer, challenges with interfacial engineering, and processing difficulties. Furthermore, rules for choosing graphene or nanotubes based on particular application needs are lacking. The study by Kinloch et al. [473] provides insights into the future trends regarding this subject by outlining different strategies to tackle these problems and highlighting the prospects and challenges in the development of high-strength, low-density, and high-conductivity materials incorporating nanotubes or graphene.
5. Biomedical Applications: Drug transport, tissue engineering, and biosensing are just a few of the biomedical applications that graphene’s special qualities and biocompatibility make possible. Because of its special physicochemical qualities, graphene is gaining attention for a variety of uses in the physical, chemical, and biological sciences according to [474]. The study by Yang et al. [475] examines the new developments in graphene-based materials for biomedical applications, with a particular emphasis on drug delivery, bioimaging, biosensors for biomolecule detection, and photothermal therapy. The review offers insights into the future trends of graphene in biological applications and also addresses the opportunities and potential obstacles in this quickly developing field.

All things considered, conductive thin films and single-layer graphene have enormous potential to transform many industries and spur innovation in the years to come. Further research and development are anticipated to lead to even more fascinating uses and business prospects for these materials.

#### 6.1.2. Double-Layer Graphene

**Figure 74:**
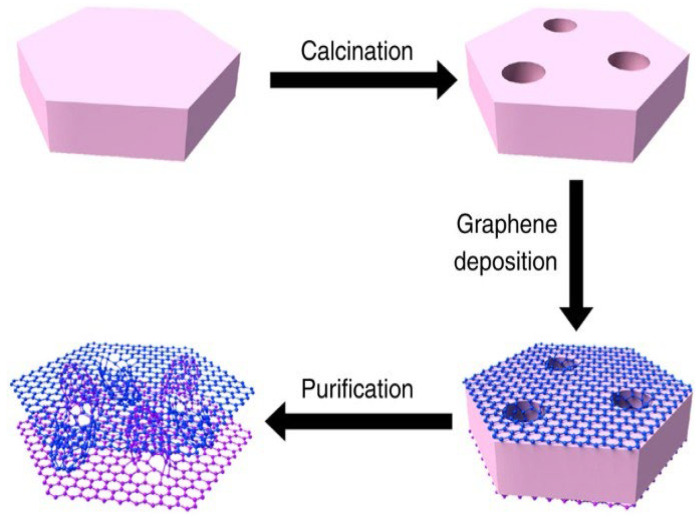
A strategy for creating unstacked DTG. Reproduced from [479] under permissions from copyright clearance center.

Two layers of graphene layered on top of one another are referred to as double-layer graphene [476]. Its distinctive electronic characteristics, which may be adjusted by applying an electric field or by twisting the layers in relation to one another at particular angles, make it significant. The formation of exotic electronic states, including correlated insulators, superconductors, and topological phases, can result from the phenomenon surrounding moiré superlattices [477]. These states are very interesting for fundamental research and may have technological uses in the future. Double-layer graphene is one method for producing conductive thin films that are both highly transparent and conductive, making it useful for solar cells, flexible electronics, and touchscreens [478]. The future direction of this material is to optimize its integration into useful devices for cutting-edge electrical and optoelectronic applications, as well as to continue investigating and comprehending the rich electronic phenomena resulting from moiré superlattices. Figure 74 represents the schematic diagram of a double-layer graphene sheet.

**Figure 75:**
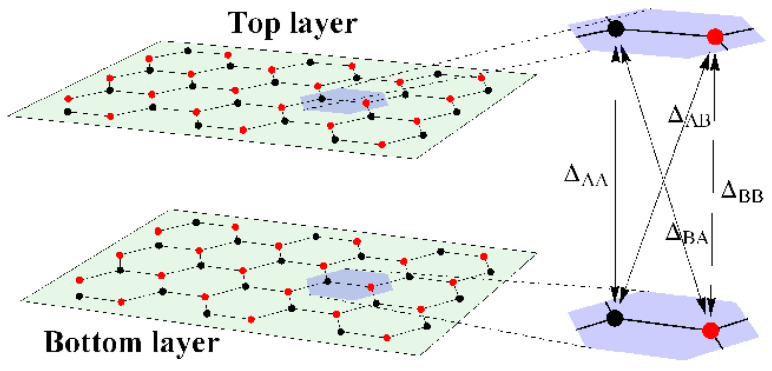
Two graphene sheets arranged in parallel constituting double-layer graphene. Reproduced from [480] under permissions from copyright clearance center.

Zhao et al. [479] described a new way to create double-layer graphene (DTG) with intrinsic protuberances that can be used in high-power lithium–sulfur (Li–S) batteries. The method is based on the use of mesoporous metal oxide nanosheets as epitaxial growth templates for unstacked graphene layers, as shown in Figure 75. Graphene protuberances are formed, and the graphene layer stacking is inhibited by the deposition of carbon atoms onto the mesoporous template. Once the template is removed, two graphene layers with many protuberances comprise the unstacked DTG. Because of its large surface area and electrical conductivity, this DTG structure is anticipated to be a useful electrode material for Li–S batteries.

### 6.2. Tungsten Disulfide (WS_2_)

**Figure 76:**
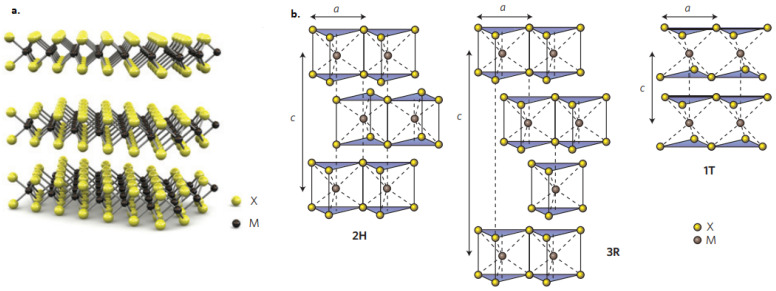
(**a**) MX_2_’s typical three-dimensional schematic representation, where M is metal, and X is chalcogen atoms ) WS_2_’s general structure; (**b**) different prototypes of WS_2_ including hexagonal symmetry (2H), rhombohedral symmetry (3R), tetragonal symmetry (1T). Reproduced from [482] under permissions from copyright clearance center.

WS_2_ has excellent potential for conductive thin-film applications because of its unique electrical properties and adaptability. WS_2_ belongs to a group of two-dimensional materials known as transition-metal dichalcogenides (TMDs), with special mechanical, optical, and electrical characteristics [481]. Figure 76 depicts the general structure, including trigonal (1T), hexagonal polymorph (2H), and rhombohedral (3R) structures [482]. The study of WS_2_ thin films is a significant movement investigating 2D materials for photonics, optoelectronics, and electronics applications. The application of WS_2_ thin films in optoelectronic and electronic devices, including photodetectors, FETs, LEDs, and photovoltaics, is being researched. Because of its direct bandgap and semiconducting nature, WS_2_ is a good fit for electronics and photonics applications [483]. Although WS_2_ thin films often behave as semiconductors, they have attracted interest due to their possible application in a wide range of conductive thin-film applications. Through the utilization of WS_2_’s remarkable mechanical flexibility, elevated thermal stability, and superior optical characteristics, scientists are investigating methods to augment its electrical conductivity. The electronic characteristics of WS_2_ thin films can be customized using defect engineering, heterostructure integration, and doping methods, which may enable them to be used as conductive components in next-generation electrical and optoelectronic systems. Wearable electronics, flexible displays, and energy-harvesting devices are just a few applications requiring lightweight, bendable, and high-performance conductive thin films. WS_2_’s compatibility with flexible substrates and scalable synthesis methods further enhances its appeal in these areas.

Enhancements in the manufacturing processes, like ALD, CVD, and solution-based techniques, have made it possible to produce superior WS_2_ thin films with exact control over their thickness, shape, and crystallinity. Scholars persistently investigate novel approaches for the scalable manufacturing and incorporation of WS_2_ thin films into apparatuses [484,485]. These patterns show the wide range of prospective uses and research avenues for WS_2_ thin films, underscoring their capacity to facilitate the development of next-generation electronics, photonics, sensing, and energy technologies. The research endeavors are presently concentrated on tackling obstacles associated with scalability, repeatability, and device integration to fully realize the potential of WS_2_ thin films in practical uses.

**Figure 77:**
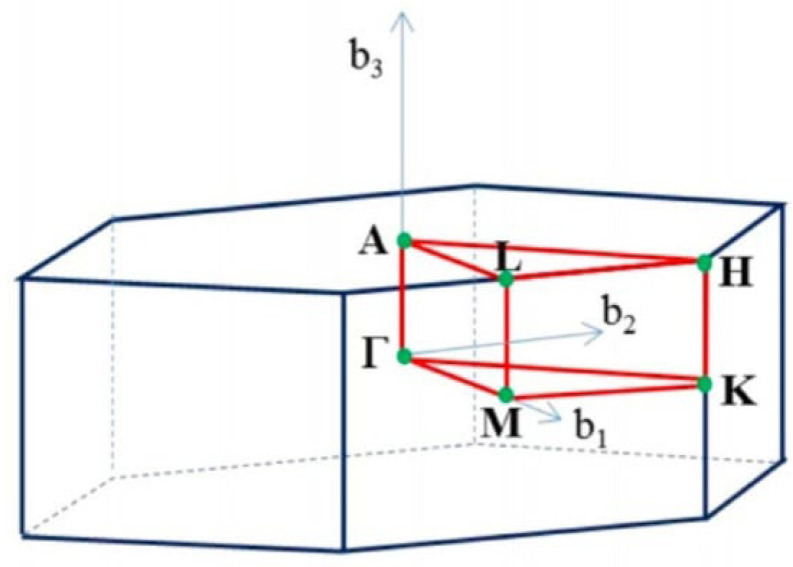
The Brillouin zone and unique point of WS_2_’s hexagonal lattice structure. Here, Γ (Gamma) is the point of reference of the Brillouin zone, and A, H, K, L, and M are high symmetry points. Reproduced from [487] under permissions from copyright clearance center.

Studying materials such as WS_2_ requires an understanding of the Brillouin zone as this enables the band structure of a single lattice to be used to reflect the band structure of the entire solid. In essence, it reduces the intricate behavior of the electrons within a crystal lattice to a simple structure. The features of the primitive cell of the lattice may be succinctly described because, as Theimed et al. [486] pointed out, the Brillouin zone represents it in reciprocal space. The researchers used the representative Brillouin zone of the WS_2’_s hexagonal lattice to demonstrate this idea in their study. The WS_2_ hexagonal lattice with a typical Brillouin zone is shown in Figure 77.

**Figure 78:**
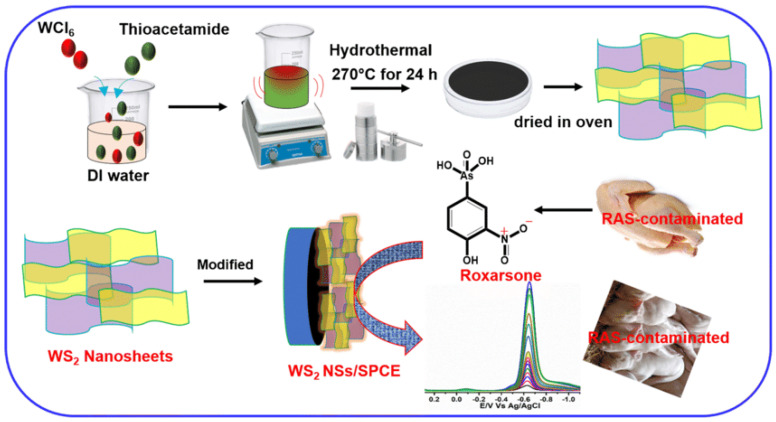
WS_2_ nanosheets’ synthesis and roxarsone’s electrochemical detection. Reproduced from [488] under permissions from copyright clearance center.

Especially for hazardous roxarsone, the production of WS_2_ nanosheets offers a potential path for electrochemical detection. These three stacked atom layer nanosheets have distinct nanostructures with partially unbound sulfur at the flaws and edges, which improves their interaction with analytes. This interaction enhances the sensor performance and stability along with WS_2_’s outstanding electron conductivity and electrocatalytic activity. When WS_2_ nanosheets are used as the foundation for electrochemical sensors, exceptional sensitivity for roxarsone detection is illustrated in Figure 78.

#### 6.2.1. Conductivity

The crystalline structure, doping, and ambient conditions of WS_2_ can all affect its conductivity. In general, WS_2_ demonstrates a variety of conductivity characteristics, ranging from insulating in bulk to semiconducting or even metallic in monolayer or few-layer configurations. Additionally, WS_2_’s conductivity can be altered by doping it or by subjecting it to outside stimuli like strain or electric fields. Doping with specific elements, which introduce charge carriers, can change its conductivity qualities. Transistors, sensors, and photovoltaics are just a few of the technical applications that can benefit from WS_2_’s adaptable material. Its conductivity can vary greatly depending on its form and the surroundings.

Nitrogen-doped conductive carbon/WS_2_ nanocomposites (WS_2_–NCs) are presented in a study as a potential anode material for sodium-ion batteries (SIBs) that improves WS_2_’s conductivity and cycling performance [489]. Nitrogen-doped carbon is added to WS_2_ nanosheets to boost the interfacial reactions and performance. This suggests that high-capacity sulfide-based anode materials for SI may be developed in the future.

Another study by Han et al. [490] examined the thermal and electrical conductivity of composites that have different amounts of WS_2_ filler, which resembles flakes distributed throughout an ethylene–propylene–diene monomer matrix. The findings demonstrate that the WS_2_ exfoliation and testing temperature affect the conductivity of the composites, with lower-WS_2_-content composites displaying non-linear conductivity. Furthermore, the inclusion of WS_2_ greatly enhances the composites’ thermal conductivity, rising by around 45% at 25 °C and 40% at 75 °C.

Zhang et al. [491] investigated how monolayer WS_2_ interacts with substrates and temperature in relation to its thermal conductivity. To guarantee measurement precision, temperature–Raman coefficient effects and laser absorption were eliminated by employing a dual-wavelength flash Raman technique. The findings indicate that, when WS_2_ is supported on a substrate as opposed to being suspended, its thermal conductivity is significantly reduced. This reduction is attributed to the suppression of acoustic phonons by molecular dynamics simulations. Understanding the substrate-induced modifications to optical phonon behavior by Raman spectroscopy helps to understand the phonon transport behavior of WS_2_. This work advances our understanding of WS_2_’s conductivity under various circumstances, which is important for the development of nanodevice applications in the future.

The study by Yu et al. [492] on the effects of partially replacing the tungsten atoms in WS_2_ and WSe_2_ with niobium provides insight into the conductivity behavior of these materials. Their results show some interesting trends in the electrical conductivity of WS_2_ and WSe_2_. Both increase exponentially with temperature, which is typical of semiconductor behavior. As an example of the typical metallic behavior, samples of W_1−x_Nb_x_Se_2_ exhibit a linear reduction in conductivity with temperature. The W_1−x_Nb_x_S_2_ samples, interestingly, show a linear increase in conductivity with temperature, indicating a unique response caused by the niobium atom addition. This work highlights the substantial influence of dopant atoms on the electrical characteristics of transition-metal dichalcogenides, leading to an astounding four-fold increase in electrical conductivity [492].

#### 6.2.2. Transmittance

WS_2_ is a well-known 2D material whose transmissivity is essential for many optical applications [493]. Some interesting optical characteristics of WS_2_ include transparency in the near-infrared range and significant absorption in the visible range. Factors including crystal quality, layer thickness, and ambient conditions affect its transmissivity. According to studies, WS_2_ can have high transmittance, which qualifies it for use in transparent conductive coatings, photodetectors, and optical modulators, among other applications [494,495]. Moreover, external stimuli like strain or electric fields can modify the transmissivity of WS_2_, opening up possibilities for dynamic control in optoelectronic systems [496]. By comprehending and utilizing WS_2_’s transmissivity, new opportunities for the development of sophisticated photonic and optoelectronic technologies with improved functionality and performance arise [497].

Bin Rafiq et al. [494] investigated the transmissivity of WS_2_ using RF magnetron sputtering on glass substrates. They produced films with a bandgap of 2.2 eV by optimal deposition. These films demonstrated promising photovoltaic performance when incorporated as window layers in CdTe/WS_2_ solar cells, suggesting that WS_2_ may be useful for increasing device efficiency.

Ye et al. [498] examined the impact of annealing temperatures (200–500 °C) on the characteristics of WS_2_ thin films formed by RF magnetron sputtering on Si and glass substrates. They used an ultraviolet spectrophotometer and a hall tester, respectively, to investigate the optical and electrical qualities. According to the results, the WS_2_ film characteristics show non-linear changes with temperature. Furthermore, the ratio of sulfur to tungsten was quite close to the optimal stoichiometric ratio of WS_2_.

**Figure 79:**
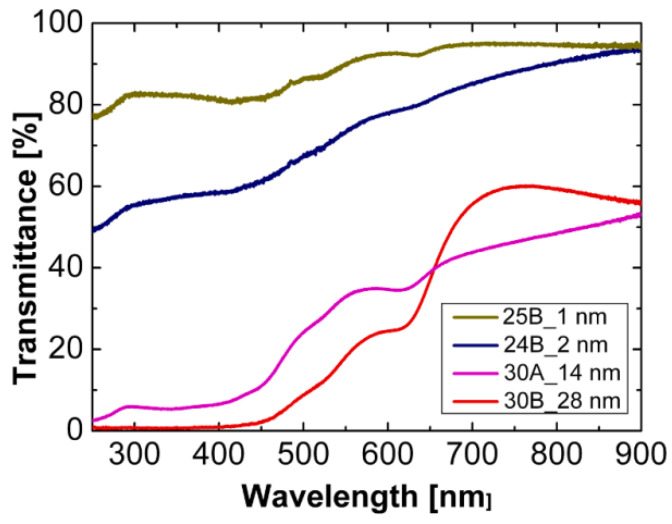
Variations in transmittance due to changes in the thickness. Reproduced from [449] under permissions from copyright clearance center.

A thorough investigation into the structural, optical, and electrical characteristics of multilayer WS_2_ films produced on tungsten substrates was conducted by Hotovy et al. [499]. They provide important insights into the behavior of these films by their analysis of optical parameters such as transmittance, absorption coefficient, and energy bandgap (E_g_). The optical characteristics were measured using c-plane sapphire substrates from 250 to 900 nm in wavelength. With transmittance values ranging from 18.3% to 89.6%, the results notably show that all the WS_2_ films demonstrate transparency. Remarkably, transmittance falls as one should expect as tungsten thickness rises, as shown in Figure 79. In addition, the (αhϑ)^2^~E − E_g_ dependence’s optical bandgap values demonstrate the complex link between film thickness and optical properties, emphasizing the layered WS_2_ films’ subtle behavior. This thickness-dependent behavior highlights the firm relationship between film thickness and optical transparency. Furthermore, the identification of these materials’ optical bandgap values provides vital information on their energy levels, enabling deeper comprehension of their optical behavior and possible uses in optoelectronic devices.

Another study by Hotovy et al. [500] examined the impact of sulfurizing sputtered W film thicknesses to create WS_2_ films on sapphire substrates. WS_2_ films with different W film thicknesses (from 4 nm to 12 nm) were investigated for their structural, morphological, and optical properties. According to the findings, all the WS_2_ films that were studied kept their transparency levels between 30 and 78% over the 500–900 nm spectral range. Furthermore, the films showed a 2.3 eV straight bandgap. Highly dense horizontally aligned flakes were observed in the thinner WS_2_ films (20 and 24 nm), but a granular surface with WS_2_ crystallites developing perpendicular to the substrate was observed in the thicker films (33 and 42 nm). This study by Hotovy et al. [500] also provides insights for enhancing the optical characteristics of WS_2_ films in a variety of applications by highlighting the significance of film thickness in altering the transmittance of these films.

### 6.3. Molybdenum Disulfide (MoS_2_)

Scientists are always looking for new materials that have the potential to completely change the industry in an effort to make electronic devices more efficient and smaller. Among these, MoS_2_ has shown great promise, mainly when applied as thin coatings [501]. MoS_2_, a member of the TMD family of transition-metal dichalcogenides, is extremely desirable for various applications in optoelectronics and electronics due to its exceptional optical, mechanical, and electrical properties [502].

**Figure 80:**
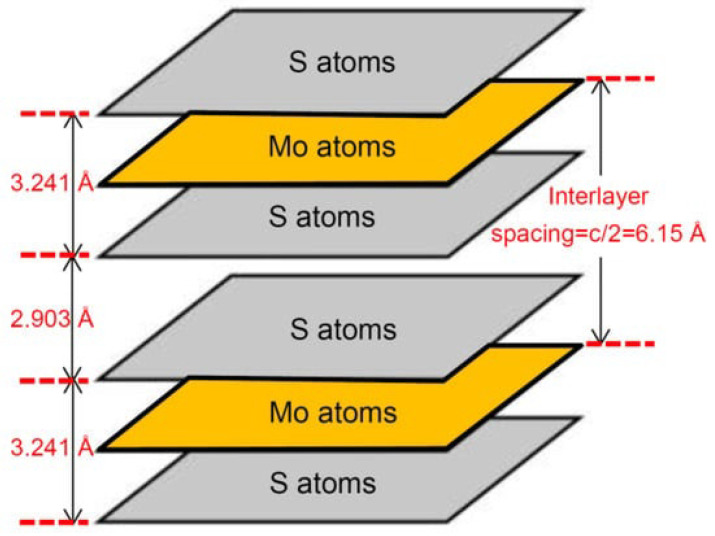
Distances in 2H MoS_2_ between several layers. Reproduced from [505] under permissions from copyright clearance center.

Different polytypes of MoS_2_ exist, with distinct structural configurations and characteristics displayed by each. The molybdenum and sulfur atoms in 2H-MoS_2_ create a hexagonal lattice structure, which is one example of this type of polytype [503]. The symmetry and layer stacking order inside the crystal structure are indicated by the “2H” designation. Layers one and two of molybdenum atoms are layered with two layers of sulfur atoms arranged hexagonally in the 2H-MoS_2_ structure, as shown in Figure 80. Regarding two-dimensional materials and nanotechnology, in particular, this polytype, one of the most prevalent structures in MoS_2_, is extensively researched due to its distinct electrical and optical properties [504].

**Figure 81:**
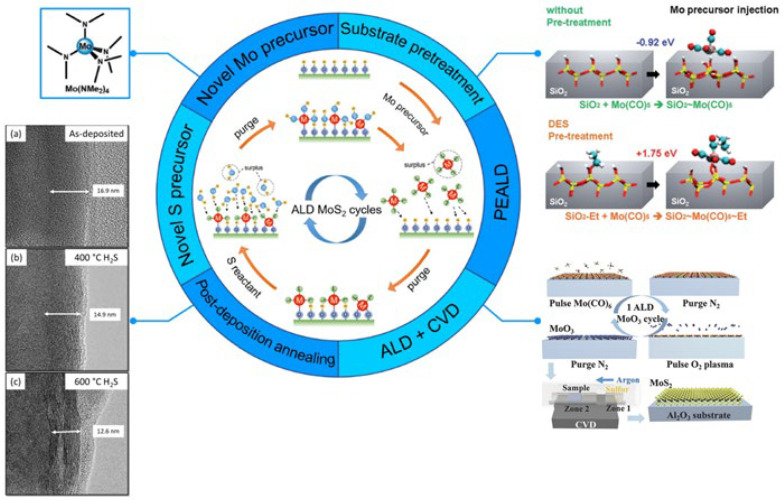
A schematic representation of the ALD synthesis process and the methods used to improve the quality of the MoS_2_ film. Cross-sectional TEM images of MoS₂ thin films following different treatments are shown in the images on the left (**a**–**c**). (**a**) MoS₂ thin film as deposited (**b**) Annealed MoS₂ thin film in H₂S at 400°C (**c**) MoS₂ thin film annealed in H₂S at 600°C. Reproduced from [506,508,509,510,511,512] under permissions from copyright clearance center.

ALD is a process that is particularly advantageous over the other synthetic methods for the preparation of 2D MoS_2_ thin films [506]. ALD offers unique advantages, such as precise layer controllability, wafer-scale homogeneity, and good conformality, even though mechanical exfoliation and chemical vapor deposition approaches have been investigated [507]. Because ALD uses two half-reactions instead of one straight gas-phase reaction, as in chemical vapor deposition methods, MoS_2_ films can be produced under more controlled conditions. One full ALD cycle is composed of the following four steps in the ALD process, as shown in Figure 81: injection of Mo precursors, purging, injection of S precursors, and purging. To precisely and consistently produce MoS_2_ thin films with the required thickness, repeat this cycle, which consists of two half-reactions and two evacuations. As a result, Figure 81, showing the ALD synthesis of MoS_2_ thin films, emphasizes the special steps in the process as well as the benefits of using this technique to achieve exact control over the properties of the film [506].

Because of their unique qualities and possible uses, MoS_2_ thin films have attracted a great deal of interest. Researchers can make use of the material’s fascinating quantum confinement characteristics by creating MoS_2_ films with nanoscale thicknesses, which will improve the material’s electrical and optical qualities over bulk MoS_2_ [513]. The electrical characteristics of MoS_2_ thin films are among their most promising features. Because of its straight bandgap, MoS_2_ is a semiconductor that facilitates effective electron transport, making it easier to create high-performance electrical devices [513]. MoS_2_-thin-film-based FETs have shown remarkable properties, such as outstanding on/off ratios, low off-state current, and great carrier mobility. Because of these characteristics, MoS_2_ FETs can be used in flexible electronics, low-power electronics, and digital logic circuits [514]. MoS_2_ thin films have fascinating optical features in addition to their electrical ones, which are essential for optoelectronic and photonic applications. Because of its straight bandgap, MoS_2_ is a good fit for photovoltaic, photodetective, and LED applications [487]. It also permits efficient light emission. Through the integration of MoS_2_ thin films into photonic structures like resonators and waveguides, scientists can create new devices for quantum information processing, sensing, and on-chip optical communication [515].

Both WS_2_ and MoS_2_ have comparable chemical structures and are widely used in a wide range of real-world applications [516]. Both materials have a variety of uses, from being viable cathodes in lithium rechargeable solid-state batteries to functioning as solid lubricants and catalysts in hydrodesulfurization procedures [517]. But, even with these commonalities, there is a major difference in how they exfoliate. Even though the methods for MoS_2_ exfoliation have proven to be effective, thoroughly exfoliating WS_2_ is still difficult [518]. This divergence emphasizes the necessity of customized strategies to successfully utilize the special qualities of WS_2_ [519].

Even with the incredible developments in the field of MoS_2_ thin films, several issues still need to be resolved. For industrial-scale applications, the large-scale synthesis of superior MoS_2_ films with consistent thickness and flawless shape is essential [520]. Optimizing the device performance and reliability also requires knowing and managing the interface qualities between MoS_2_ and other materials. MoS_2_-thin-film technology looks to have a promising future because research is being conducted to solve these issues and realize the material’s full potential [521]. With further progress regarding the fabrication methods, device architecture, and basic knowledge, MoS_2_ thin films are expected to revolutionize the creation of next-generation electronics, photonics, and other fields.

#### 6.3.1. Conductivity

MoS_2_ thin films’ unique conductivity characteristics, which result from their two-dimensionality and semiconducting behavior, have attracted a great deal of attention. The electrical conductivity of these films may be adjusted, and they can switch between metallic and semiconducting states based on many factors, such as layer thickness, doping, and ambient conditions. MoS_2_’s intrinsic bandgap facilitates effective electron transport, rendering it appropriate for use in transparent conductive coatings, photodetectors, and FETs. Moreover, MoS_2_’s potential for high-speed and low-power electronics is highlighted by its outstanding on/off ratios and strong carrier mobility in FET devices. Research activities are still being conducted to improve the conductivity and maximize the performance of MoS_2_ thin films and prepare them for incorporation into next-generation electrical and optoelectronic devices.

Wang et al. [522] emphasized how important conductivity is to enhancing the MoS_2_-based anode materials’ electrochemical performance for lithium-ion batteries. The researchers successfully overcame the problem of low electronic conductivity in pure MoS_2_ by creating Mn-doped MoS_2_ nanosheets. The electrode showed improved electrochemical characteristics, such as a high-rate capacity, long-term cycle stability, and initial discharge capacity. The enhanced performance is ascribed to the combined effects of Mn doping and the modified composite structure. In addition to offering a greater surface area for efficient electrolyte penetration, the hierarchical carbon skeleton inhibits nanosheet aggregation during cycling. Moreover, by enabling electronic injection, the Mn doping raises the electronic conductivity. This work opens the door for improvements in lithium-ion batteries by highlighting the vital role that conductivity plays in maximizing the performance of MoS_2_-based anode materials [522].

Another study showed difficulties in making electrical contact with very anisotropic layer topologies, such as MoS_2_ [523]. It provides clarification on experimental results using natural molybdenite crystals through analysis. According to the study, annealed contacts on MoS_2_ facilitate contact with all the layers by distributing the material parallel to the c-axis, resulting in good electrical contact. It provides information about conductivity behavior by locating activation energies parallel and perpendicular to the c-axis. The results obtained from this study also harmonize with the published data on MoTe_2_ thick and thin crystals, improving our comprehension of the electrical characteristics of these materials [523].

Mao et al. [524] investigated the effects of vanadium doping on the thermoelectric characteristics of MoS_2_, a material that shows great promise due to its high Seebeck coefficient and low thermal conductivity. In terms of acceptors, V doping dramatically increases electrical conductivity by orders of magnitude in carrier concentration. V doping also introduces many point defects and encourages grain refinement, thus lowering lattice thermal conductivity. However, the optical and electrical characteristics of MoS_2_ and Ni-MoS_2_ crystal layers created using the chemical vapor transport method were investigated by Ko et al. [525]. The measurements of the temperature-dependent optical reflectance for both samples show two excitonic transitions. In comparison with MoS_2_, Ni-MoS_2_ displays n-type behavior, with higher resistance and lesser mobility, according to the Hall effect tests. Ni doping at 1.2 eV was revealed in the photoconductivity spectra, which increases photocurrent production in Ni-MoS_2_. Temperature-dependent conductivity reveals two shallow levels with activation energies, suggesting that Ni doping causes smaller activation energies, reduced mobility, and increased resistance. These results shed light on how Ni doping affects the optical and electrical characteristics of MoS_2_, offering important directions for the creation of MoS_2_ optoelectronic devices.

In a study by Yang et al. [526], the thermal conductivity and rectification impact of MoS_2_ were investigated. They tested the thermal conductivity of monolayer and multilayer MoS_2_ films and calculated the thermal rectification coefficient using suspended microelectrodes. The differences between the monolayer and multilayer MoS_2_ films were compared using numerical simulations. Furthermore, the correlation between geometrical asymmetry and the thermal rectification coefficient was investigated on samples with varying angles. The results showed that the thermal conductivity of monolayer MoS_2_ is higher than that of multilayer MoS_2_ and that both decrease with increasing temperature and layer thickness. The research by Yang et al. [526] clarified the thermal behavior of MoS_2_ and its possible uses in nanoscale heat transfer and thermal control. Moreover, a published article by Yuan et al. [527] provided insights into the electrical characteristics of TMDs and their possible uses in optoelectronic devices, furthering our understanding of how defects in crystal structure impact conductivity.

#### 6.3.2. Transmittance

MoS_2_, a two-dimensional semiconducting material, presents an exciting area of research due to its fascinating optical characteristics. Its layer thickness, doping, and ambient conditions are among the elements that affect its transmittance, or capacity to let light through. The transparency of MoS_2_ thin films has been thoroughly investigated across a broad spectrum, spanning from ultraviolet to infrared. These films have demonstrated potential for use in optoelectronics, where the creation of transparent conductive coatings, photodetectors, and light-emitting diodes is made possible by their tunable transmittance. For MoS_2_ to be used in next-generation optical devices and technologies, its transmittance must be understood and controlled.

Zhang et al. [528] revealed the effects of the annealing temperature on the characteristics of films of MoS_2_ grown on silicon and glass substrates by RF magnetron sputtering. The MoS_2_ films’ surface morphology, structure, transmission, and reflectivity were evaluated using spectrometry, XRD, and SEM methods. The findings show that the annealing temperature highly influences the MoS_2_ thin film’s surface shape and XRD diffraction peak position. Elevated annealing temperatures enhance growth quality and MoS_2_ film transmission and reflection. This study clarifies the variables affecting MoS_2_ film transmittance and offers suggestions for improving its characteristics for a range of uses. Chen et al. [529] investigated the effects of annealing temperature and deposition on the optical characteristics of MoS_2_ films deposited by radio-frequency magnetron sputtering. Increased deposition temperatures result in better crystallization and stronger coupling, but annealing modifies the structure of the film, especially the bonding associated with sulfur. The optical bandgaps show semiconductor properties ranging from 0.92 eV to 1.15 eV. After annealing at 200 °C, there is a modest reduction in the optical bandgaps because of blue shifts in the K point of the Brillouin zone. These results provide information on how to regulate the optical characteristics of MoS_2_ films, which are important for a variety of optoelectronic applications, by optimizing the annealing and deposition conditions.

**Figure 82:**
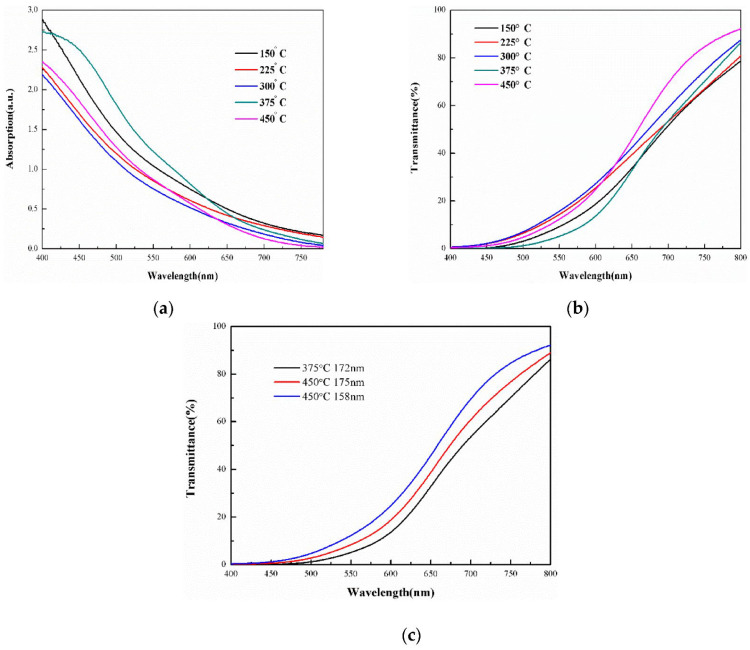
The absorption and transmission spectra of thin-film MoS_2_ deposited at different temperatures due to a decrease in layer thickness; the absorption spectra in (**a**) show a decrease in absorption as the deposition temperature rises up to 300 °C. As the deposition temperature rises, the transmittance spectra in (**b**) show a proportional increase in transmission. Transmittance spectra for three samples are also shown in (**c**), indicating that Mo-O bond formation, in addition to thickness, affects transmittance variations at higher temperatures. Reproduced from [530] under permissions from copyright clearance center.

The deposition temperature of MoS_2_ thin films affects their optical properties, especially transmittance, as revealed by Wang et al.’s study [530]. They detected notable changes in the absorbance and transmission spectra by applying the RF magnetron sputtering method to deposit MoS_2_ thin films at different temperatures, as shown in Figure 82. It is noteworthy that the absorbance reduced and transmission increased as the deposition temperature increased from 150 °C to 300 °C. This is likely because the decreased deposition rates led to a reduction in the film thickness. The curves for the samples at 375 °C and 450 °C crossed the earlier curves, indicating departures from this pattern at temperatures above 300 °C. The subsequent tests showed that the creation of Mo-O bonds contributed to changes in the transmittance spectra at higher temperatures in addition to differences in film thickness, as shown in Figure 82c. This underlines the complex relationship between deposition conditions and film properties by indicating that both thickness and the creation of Mo-O bonds control the optical properties of MoS_2_ thin films.

Park et al. [531] revealed that MoS_2_ films with a critical thickness demonstrated high optical transparency (>90%). This suggests that the MoS_2_ films retain exceptional transparency even at multilayer thicknesses, which qualifies them for use in transparent electronics applications. MoS_2_ nanostructure transmittance varies from 0.92 to 0.68 depending on the film thickness, as demonstrated by Sachidanand et al. [532]. Lower transmittance values were observed in thicker films, suggesting that film thickness affects MoS_2_’s optical characteristics.

Another study by Yoon et al. [533] showed that the developed transistor had a high optical transmittance of roughly 74%. It had exfoliated MoS_2_ as the channel and CVD-grown graphene as the source and drain electrodes. This shows that the transistor functions well while maintaining outstanding transparency, which qualifies it for uses where transparency is required, including transparent electronics or display technologies.

Since transmittance directly correlates with optical characteristics, it is essential for MoS_2_ thin films. Because MoS_2_ is a semiconductor material with unique optoelectronic properties, transmittance is crucial in devices, including photodetectors, solar cells, and optical modulators [534,535]. For various optoelectronic applications, improving the device performance and efficiency requires understanding and focusing on maximizing the transmittance in MoS_2_ thin films.

### 6.4. Silver-Nanowire-Based Electrodes for Flexible TCFs

AgNW-based electrodes are critical for developing flexible TCFs due to their excellent conductivity and transparency. The deposition technique and the intrinsic characteristics of the AgNWs have significantly impacted these TCFs’ performance. AgNW TCFs, or silver nanowire transparent conductive films, have garnered more interest recently because of their potential uses in organic optoelectronic devices, transparent sensors, film heaters, and other applications [536]. The traditional use of ITO transparent conductive films is due to their comparatively high optoelectrical characteristics and well-established production procedure [40]. However, there are certain drawbacks to using ITO TCFs, such as their high cost of fabrication, resource scarcity, inherent brittleness, and high work function, which reduces charge carrier injection, particularly in organic optoelectronic devices [537,538].

#### Tips for Optimizing AgNW-Based TCFs

The performance of TCFs largely depends on the deposition method and intrinsic properties of AgNWs. For AgNW-based TCFs to have the best possible properties, take into account the suggestions that follow:

1. Length of Nanowire:
  - Long AgNWs: These improve conductivity and lessen electrical resistance by reducing the number of junctions in the network. They are perfect for situations where strong conductivity is essential [539].
  - Short AgNWs: They are more straightforward to handle but often have more connections, which could lead to increased resistance. To lessen this, make sure the density is higher or maximize the uniformity of the deposition [540].
2. Arrangement of Nanowires:
  - Uniform Arrangement: The electrical conductivity and optical transparency increase when AgNWs are distributed uniformly throughout the substrate. Methods such as controlled spin-coating can achieve this homogeneity [541].
  - Random Arrangement: When nanowires are positioned randomly, hotspots of high resistance and inconsistent performance could exist across the film. This can be resolved by applying post-treatment techniques to enhance the connection or streamline the deposition process [542].
3. Density of Deposition: Careful control of the AgNW content in the solution and the deposition conditions is necessary to balance transparency and conductivity. A network that is too dense could be less transparent, whereas one that is too sparse might not have enough conductivity [543].
4. Surface Coatings: Applying coatings like graphene (AgNW-G) or zinc oxide after deposition strengthens the electrical connectivity at the nanowire junctions and increases mechanical stability, both of which are advantageous in flexible or stretchable applications [544].

AgNW properties like length and arrangement significantly influence how well they can be deposited using different techniques. These elements affect the final TCFs’ conductivity, transparency, and homogeneity. To guarantee that the films match the intended performance criteria, each deposition method requires careful optimization of the process parameters to consider the unique characteristics of the AgNWs being utilized.

## 7. Challenges and Opportunities

Thin-film technology, at the frontier of materials science and engineering, offers an exciting landscape of prospects and problems. Researchers face many challenges as they continue to explore thin films, from improving the electrical conductivity and contact stability to precisely controlling the film quality and thickness. To overcome these obstacles, creative thinking and state-of-the-art methods are required. But, these difficulties also present many opportunities for scientific and technical progress. Thin films have uses in electronics, energy storage, catalysis, and other fields. They are incredibly versatile and functional. Researchers have the opportunity to transform the current technologies and open the door for ground-breaking discoveries with significant effects by utilizing the potential of thin-film materials.

### 7.1. Challenges

Conductive thin films lead modern technological breakthroughs because of their outstanding characteristics and wide range of applications. Their adaptability across various industries, from solar cells to flexible electronics, fuels innovation. However, the prospects are accompanied by problems in each emerging aspect, so researchers and engineers must traverse challenging areas to realize their full potential. Many authors have addressed different challenges, and their possible solutions are also suggested, as shown in Table 12.

### 7.2. Opportunities

There are several applications for conductive thin films in many different sectors. With their abundance of prospects in several fields, these films represent the heights of innovative research. These films have paved the path for innovation and technological growth and have transformed many industries. They are distinguished by their exceptional electrical conductivity and frequent nanoscale thickness. Below are some of the most promising sectors that have significant potential.

1. Flexible electronics: Flexible electronics represent one of the most promising applications of conductive thin films. Their natural flexibility opens the door to a new era of wearable and flexible electronics by creating roll-up solar cells, bendable screens, and wearable sensors [551].
2. Energy technologies: Conductive thin films are essential for improving the efficiency of batteries, supercapacitors, and solar cells [552]. These films help to improve energy conversion and storage efficiency by enhancing electron mobility and charge transport.
3. Biomedical applications: Due to their electrical properties and biocompatibility, conductive thin films have unique possibilities for various applications in biomedical devices. These films have enormous potential to transform healthcare technology, from drug delivery systems to biosensors and neurological probes [553,554].
4. Manufacturing advancements: The printability of conductive thin films makes large-scale manufacturing processes like inkjet and screen printing easier [555]. This paves the way for the affordable and scalable manufacturing of electronic components, opening up new opportunities.
5. Photonic devices: Photonic devices, such as OLEDs, LEDs, and photodetectors, use conductive thin films. Their capacity to regulate the flow of photons makes improvements in lighting, displays, and optical communications possible, thus also improving these devices’ efficiency and performance [556,557].
6. Flexible sensors: Stretchable and flexible sensors are being developed for various uses, and conductive thin films play a vital role in this process [558]. Utilizing the characteristics of thin films, these sensors identify and transfer essential data in wearable health monitors, fitness trackers, environmental sensors, and human–machine interfaces.
7. Aerospace and defense: Conductive thin films are essential for temperature management, radar-absorbing materials, and electromagnetic interference (EMI) shielding in these sectors [559]. Because of their conductive, lightweight, and firm characteristics, they are perfect for improving the dependability and performance of military hardware, satellites, and airplanes [560].
8. Automotive electronics: Conductive thin films are helpful in the automotive industry for touch-sensitive controls, transparent conductive coatings for windows and displays, and parts for electric vehicles [561]. These films fuel innovation in the automobile sector, improving safety systems, energy economy, and vehicle connectivity.
9. Environmental monitoring: Conductive thin films are an essential component of environmental monitoring systems. They make it easier to identify and analyze pollutants, gases, and contaminants. Thin-film sensors support environmental conservation and protection initiatives by helping with environmental surveillance, water quality evaluation, and air quality monitoring [562].
10. Smart textiles: Incorporating conductive thin films into textiles makes wearable electronics and smart clothing possible. With their seamless integration of electronics into regular clothing, these textiles transform sports, fashion, and healthcare. They can track physical activity, monitor vital signs, and provide interactive functions [563].
11. Transparent conductors: Transparent conductive films provide the conductivity and transparency needed for responsive touch interfaces. They are utilized in touchscreens found in smartphones, tablets, and other devices [555]. They also play a crucial role as transparent electrodes in optoelectronic devices, including OPVs and LEDs.
12. Radio-frequency identification (RFID) and antennas: When the conventional rigid antennas are impractical, conductive thin films are employed to create flexible antennas for wearable technology, drones, and Internet of Things (IoT) applications [564]. These films can also create RFID tags, which are necessary for contactless payments, supply chain tracking, and inventory management.
13. Imaging sensors: Infrared and UV light sensors are essential for applications including night vision, environmental monitoring, and medical imaging. These sensors are composed of conductive thin films [565]. In terms of high-resolution images, conductive thin films can be utilized to improve the sensitivity and resolution of the detectors in image sensors, especially for industrial or medical inspection, enabling more precise and detailed imaging.
14. Anti-counterfeiting and security and safe packaging: To provide tamper-evident seals or integrate RFID technology for tracking and identification, conductive thin films can be used in packing materials. This helps to ensure product integrity and prevent counterfeiting. These films can be utilized to create smart labels, providing additional security for expensive or delicate goods by changing the color or displaying information in response to specific stimuli [566].

These additional opportunities demonstrate the wide-ranging influence of conductive thin films on various cutting-edge technologies, spurring innovations in sectors like computers, healthcare, and aerospace. These films’ adaptability creates new opportunities for electronics, energy, and other fields in the future. As materials science and nanotechnology research progresses, the possibilities for conductive thin films and their new technological developments are virtually limitless. Seizing these opportunities could influence the future directions of electronics, energy, healthcare, and other industries.

## 8. Conclusions

This review elaborately discussed different conductive thin films, their preparation methods, optical properties, electronic properties, and the possible future trends regarding thin films. The summary of the review paper is presented below:

1. The review delved into the preparation of thin films through various methods. The preparation methods included the most commonly used techniques: PVD, CVD, ALD, CBD, spin-coating, electrodeposition, sol–gel deposition, spray pyrolysis, and PLD. Each method was explored thoroughly, highlighting the respective advantages and drawbacks. A comprehensive understanding of the techniques used in conductive thin-film preparation was outlined.
2. The optical properties, including the absorption coefficient, transmittance, reflectance, and refractive index, were discussed for the different conductive thin films in this article. The variations in the aforementioned properties with the changes in the atomic concentrations, doping concentrations, temperature (annealing temperature, substrate temperature, etc.), surface morphology, bandgap, packing density, porosity, wavelength, molarity, deposition techniques, film thickness, power and deposition time, etc., were discussed briefly. Numerous research studies were investigated and discussed to understand the underlying factors responsible for the alterations observed in the optical characteristics of the different thin films.
3. The electronic resistivity for the different conductive thin films was discussed thoroughly. The variations in the electronic resistivity due to various influencing parameters, including temperature, deposition techniques, film thickness, power and deposition time, grain size, sputtering voltage, carrier concentration, etc., were discussed. Additionally, electronic resistivity’s inverse relationship with electronic conductivity was also highlighted in this article.
4. The review article also identified the potential future trends regarding conductive thin films, focusing on 2D TCF materials such as graphene, WS_2_, MoS_2_, and AgNW. Some challenges related to conductive thin films were discussed, along with some potential solutions by other authors.

## Figures and Tables

**Figure 59:**
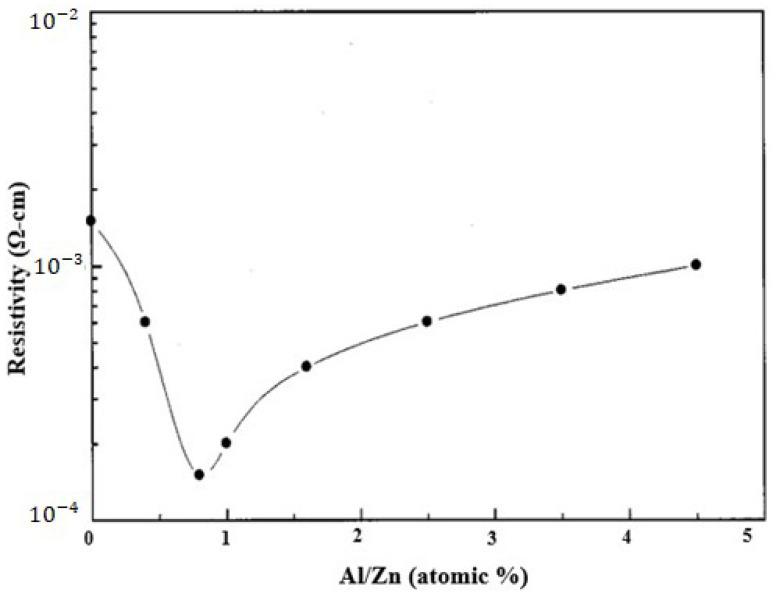
The correlation between AZO thin films’ resistance and aluminum dopant concentration in the solution. Reproduced from [214] under permissions from copyright clearance center.

**Table 1:** Different types of thin-film deposition techniques.

Vacuum-based	Physical Vapor Deposition
	Low-Pressure Chemical Vapor Deposition
	Plasma-Enhanced Chemical Vapor
	Hot-wall and cold-wall CVD
	Atomic Layer Deposition
Solution-Processed (non-vacuum)	Atmospheric Pressure Chemical Vapor Deposition
	Chemical Bath Deposition
	Spin-coating
	Electrodeposition
	Sol–gel Process
	Spray Pyrolysis
	Anodization Process
Mixed Category	Metal–Organic Chemical Vapor Deposition

**Table 2:** Comparison of different evaporation processes that are used in preparing conductive thin films.

Deposition process	Resistance heating evaporation	Laser evaporation/laser ablation	MBE	EBE	Thermal evaporation
Source of energy	Electrical resistance heating	Laser	E-beam or thermal heating	Electron beam bombardment	Thermal Heating
Substrate heating	Generally, not required	Heating can be required	Generally, not required	Generally, not required	Heating can be required
Commonly used substrate	Quartz [74], Glass [75], silicon [76]	Glass [77,78], Silicon [79,80]	GaAs [81], Silicon Wafer [82]	Glass [83]	Glass [84], Silicon [85]
Rate of deposition	Moderate to high	High	Moderate to high	High	Moderate to high
Uniformity and deposition quality	Good	Good	Excellent	Good	Good
Control of film thickness	Good	Good	Excellent	Excellent	Good
Vacuum requirements	High (~10^−6^ Torr) [86]	High	High vacuum to Ultra-high (10^−8^ to 10^−12^ Torr) [87]	High (7.5 × 10^−5^ Torr) [88]	High (~10^−6^ Torr) [86]
Complexity in the process	Mostly low to moderate	Moderate	Generally high	Moderate	Mostly Low to moderate

**Table 3:** Comparison of various sputtering processes and their impact on the quality of thin films.

Sputtering Method	Thin-Film Quality	Key Characteristics
RF Sputtering	Strong adhesion and high homogeneity; similar to Magnetron but more versatile for substrates that are heat sensitive.	Versatile, suitable for heat-sensitive substrates [118,120].
Reactive Sputtering	Controlled stoichiometry is better than DC but less effective than Magnetron; it is perfect for compound synthesis.	Precise control over the composition of the film, particularly for nitrides and oxides [106].
Ion Beam Sputtering	Finer control than RF and Reactive; exceptional precision, very uniform, and defect-free.	Ideal for films without defects, with the best control over film characteristics [123,124].
DC Diode and Triode Sputtering	Good homogeneity and adherence, optimal for conductive materials, limited control over complex formulations.	Effective for conductive films, easy to set up [117].
Magnetron Sputtering	Better than DC in complicated formulations, it combines scalability and quality with high uniformity, great adhesion, and efficient deposition.	Scalable, effective, and featuring improved control over ionization [128,129].

**Table 4:** Different types of anodization methods.

Classification Type	Different Anodization Methods
Electrolyte type	Chromic Acid Anodizing
	Sulfuric Acid Anodizing
	Hard Anodizing or Hardcoat Anodizing
	Phosphoric Acid Anodizing
	Oxalic Acid Anodizing
Anodization Conditions	Low-Voltage Anodization
	High-Voltage Anodization
Temperature of the Anodization Process	Room-Temperature Anodization
	Low-Temperature Anodization
	High-Temperature Anodization

**Table 5:** Conductive thin films with their most commonly used methods.

TCFs	Authors	Deposition Methods	Major Applications
CuI	Shi et al. [55], Moditswe et al. [54], Kaushik et al. [56]	Thermal evaporation	Nanowires (Nanodevices and Sensors), Nanoscale electric circuits, Solar cells
	Tanaka et al. [187], Yang et al. [188]	Sputtering	
	Sirimanne et al. [189], Zhu and Zhao [190]	PLD	
	Amalina et al. [191]	Spray pyrolysis	
	Sankapal et al. [192], Kui Zhao [193]	CBD	
	Inudo et al. [194]	Spin-coating	
ITO	Du et al. [195]	Thermal evaporation	CIGS solar cells, Batteries, Flat panel displays, Smart windows, LEDs, Optoelectronic devices
	Chu et al. [196], REN et al. [197], Parida et al. [198], Liu et al. [199], Gwamuri et al. [200]	Sputtering	
	Fallah et al. [201], Senthilkumar et al. [202]	EBE	
	Rozati et al. [203], Parthiban et al. [204]	Spray pyrolysis	
	Ma et al. [205]	Reactive evaporation	
	Adurodija [206]	PLD	
	Savu et al. [70], Coutal et al. [207], Kim et al. [208], Ngaffo et al. [71]	Laser ablation	
	Machet et al. [209]	Ion Plating Technique	
AZO	Gareso et al. [210], Mia et al. [211], Silva et al. [212], MAMAT et al. [213], Alam et al. [214]	Sol–gel	Solar Cells, OLED displays, General illumination, Transparent heaters
	Caglar et al. [215], Muiva et al. [216], Babu et al. [217], Lee et al. [218]	Spray pyrolysis	
	Barhoumi et al. [219], Leem et al. [220], Mosbah et al. [221], Miao et al. [222], Reddy et al. [223], Minami et al. [224], Wang et al. [225], Suzuki et al. [226], Ghorannevis et al. [227], SHEN et al. [228], Fang et al. [229]	Sputtering	
	Kaur et al. [230]	PLD	
	Tynell et al. [231]	ALD	
Si-doped GaAs	Tok et al. [232], Ploog et al. [233], Georgakilas et al. [234], Galiev et al. [235], Cortas et al. [236], Briones et al. [237], Skromme et al. [238], Li et al. [239], Ruhstorfer et al. [240], Shimanoe et al. [241]	MBE	High-speed electronic devices, Telecommunications, Optical fiber communication, Photovoltaic applications, Monolithic Microwave Integrated Circuits (MMICs), Integrated circuits
	Müller et al. [242]	PLD	
	Şenay et al. [243]	Thermionic Vacuum Arc	
TiN	Pomar et al. [244], Kavitha et al. [245], Solovan et al. [246], Yang et al. [247], Jeyachandran et al. [248], Yu et al. [249], Kharitonov et al. [250], Mascaretti et al. [251], Ponon et al. [252], Elstner et al. [253], Su et al. [254], Saoula et al. [255]	Sputtering	Decorative coatings, Tool coatings, Hard coatings, Dental tools, Surgical instruments, Infrared filters
	Chou et al. [256]	Ion plating	
	Snyder et al. [257]	ALD	
Ga doped ZnO	Yamada et al. [258], Yamamoto et al. [259]	Ion plating	LEDs, solar cells, Gas sensors, wearable electronics, Photovoltaic devices, Flexible displays and electronics

**Table 6:** Different conductive thin films with their properties.

Carrier concentration	High, around 9.41 × 10^19^ cm^3^ [269]	High, in the range of ∼10^20^ to ∼10^19^ cm^−3^ [270]	High	High, carrier density is found in the range of 10^15^ to 10^19^ cm^−3^ [271]	High, mobilities around 0.5–2 cm^2^ V^−1^ s^−1^ [194]	High, around 1.07 × 10^21^ cm^−3^ [272]
Electrical resistivity	Zhou et al. found that the lowest electrical resistance was 3.2 × 10^2^ Ω cm with 1% Al [269]	Low, 2^−4^ × 10^−4^ Ω cm [273]	Single crystal TiN shows resistivity of 23 μΩ, whereas polycrystalline shows 1000 μΩ [274]	As low as −2.6 × 10^−3^ Ω cm is obtained [275]	The resistivity is of the order of 10^−2^ Ω cm [56,196]	Low around 1.84 × 10^–4^ Ω cm [276]
Intrinsic/Extrinsic	Extrinsic	Extrinsic	Intrinsic	Extrinsic	Intrinsic	Extrinsic
Bandgap	3.3–3.4 eV, direct bandgap [221,224]	~3.6 eV, direct bandgap [277,278,279,280,281]	3.35–3.45 eV, direct bandgap [250]	Depending on the doping concentration, it shows the Burstein—Moss shift. Typically have narrow band direct bandgap of 1.25 eV [247]	~3.1 eV, direct bandgap [282]	~3.43 eV [283]
Transparency region	High optical transmittance in the near-infrared. Region approximately 300–1500 nm [284]	Transparent in the visible spectrum. Region approximately 400–700 nm [285]	Opaque, low transmittance in the visible spectrum. Region approximately 350–800 nm [286]	High transparency over a wide spectrum. Region approximately 800 nm to 1.7 μm [287]	Extremely high transmittance (>70%) in the visible region, approximately 400–700 nm [288]	Highly transparent, shows >80% visible light (400–800 nm) transmittance [289]
Material	Zinc oxide with aluminum dopant	INO with tin dopant	Titanium and nitrogen	Gallium arsenide with silicon Si dopant	Copper and iodine	Zinc oxide with gallium dopant
Thin film	AZO	ITO	TiN	Si-doped GaAs	CuI	GZO

**Table 8:** An overview of the impact of major factors on the transmittance of conductive thin films.

Thin Films	Factors	Effects on Transmittance	Authors
AZO	Doping	With increasing doping concentrations, the transmittance usually decreases.	Wang et al. [347], Alam and Cameron [214], Babu et al. [217]
		Higher doping levels (>2 at. %) may cause more photons to be scattered by doped crystal defects, causing a drop in transmittance Photons’ free carrier absorption causes a decrease in the optical transmission of strongly doped materials	Lee et al. [350]
		No matter the doping concentration, all films have high transparency in the visible (400–750 nm) optical spectrum, with values as high as 85% Bulk ZnO’s higher energy bandgap causes its transmittance to be higher in the visible spectrum	Peng et al. [382], Caglar et al. [215]
		The doped film containing 2 at. % Al has a transmittance over 90% higher than the doped film containing 3 and 4 at. %. This could be because there are more voids in the film with 2 at % than in the films with 3 and 4 at % doping. This resulted in an increase in transmittance and a decrease in optical scattering.	Babu et al. [217]
	Temperature	ZnO: Al films exhibit enhanced optical transmission in the visible spectrum upon annealing at elevated temperatures.	Alam and Cameron [214]
		Elevated substrate temperatures have the potential to enhance film adherence and impact the formation of crystalline formations, which in turn affects the optical qualities. During film growth or annealing, a significant temperature differential between the substrate and the surrounding air could introduce thermal stress. Thermal stress can affect the structural integrity of the film and, in turn, its optical performance.	Babu et al. [217]
		As substrate temperature increased, the average optical transmittance changed from 78% to 90%. The average grain size of the film may have increased, which could be the cause of the transmission increase. The increased homogeneity and crystallinity of AZO thin films can be credited with improving transmittance.	Barhoumi et al. [219]
		The visible transmittance (∼85%) stays almost constant as the annealing temperature rises from 500 to 650 ºC, while the infrared transmittance (from 780 to 2500 nm) significantly improves from 22% for the as-deposited film to 58% at 600 ºC and 71% at 650 °C for the annealed films. The films’ transmittance and crystallinity were enhanced by the high-temperature annealing	Gao et al. [348], Takci et al. [349]
	Thickness	Transmittance may decrease due to increased light absorption in the film caused by thickness.	Subba Reddy et al. [223], Lin et al. [346]
		The transmittance dropped to 50–70% from 70–90% when the deposited film thickness increased.	Hoon et al. [346]
		The films’ optical transmittance rose as the film thickness increased to 231 nm and dropped to a higher thickness of 398 nm.	Subba Reddy et al. [223]
	Surface morphology	The enhanced photon scattering caused by doping-induced crystal defects may cause the transmittance to drop at higher doping levels Film thickness can influence ZnO-thin-film surface morphology. Grain and roughness patterns on the surface may be more noticeable in thicker coatings.	Babu et al. [217]
	Coating	The transmittance is independent of the type or process of coating. The quantity of coatings had no appreciable impact on transmittances or bandgaps	Silva and Darbello Zaniquelli [212]
	Tilted angle	Despite the distorted structure, an average transmittance of >90% was achieved, demonstrating the generally high transparency of AZO film on a glass substrate.	Leem and Yu [220]
ITO	Film thickness	As ITO thin-film thickness increases, its transmittance decreases. The influence of grain size may be connected to this phenomenon. Light scattering results from an increase in grain size brought on by a thin film’s thickness. This phenomenon also could be attributed to free carrier absorption, which raises the concentration of carriers in thick films and enhances light absorption.	Eshaghi and Graeli [278]
		The measurements reveal strong transmittance throughout the visible spectrum with decreasing thickness. The transmittance decreases because of the high carrier concentration at higher wavelengths.	Maniscalco et al. [351]
	Changing compositions	When Sn doping concentration rises, optical tests indicate a modest decrease in transmittance. In the visible spectrum, the films’ transmittance showed a noticeable rise. The transmission peaked for undoped In_2_O_3_ and declined as the quantity of Sn increased. The reduction in light transmission was linked to (i) scattering at grain boundaries and (ii) oxygen vacancies. Since every film was deposited in an oxygen-rich environment and under identical processing circumstances, it was anticipated to experience the same transmission loss from oxygen flaws.	Senthilkumar et al. [202]
	Grain size	With the increasing concentration of Sn, the grain size decreased, resulting in a decrease in the transmittance. More grain boundaries and increased Sn dopant content increased scattering centers, leading to a reduction in transmission.	Senthilkumar et al. [202], Ogihara et al. [353], Katsube et al. [354]
	Deposition power and time	With the increment of the deposition time or power, a decrease in the average transmission is observed. The film thickness’s increment causes the transmission decrement The average transmittance fluctuates between 70% and 95% and decreases with increasing RF strength and deposition time.	Tchenka et al. [325]
CuI	Deposition technique	Thermal evaporation increases transmission by 70% in the 550–1000 nm wavelength range compared to other techniques. PLD technique provides the highest amount of transmittance	Kaushik et al. [56], Potts et al. [356], Burns et al. [357]
	Temperature	With the increasing substrate temperature, the transmittance increased. CuI thin-film structure and characteristics deteriorate with rising substrate temperature, as demonstrated by phase structure breaking and average transmittance falling.	Zi et al. [359], Zhu and Zhao [190]
		The transmittance decreased with the increasing annealing temperature. With an average transmittance of roughly 70–80%, all films are extremely transparent in the visible spectrum.	Moditswe et al. [54]
	Doping concentration	I_2_ doping of 1–5% shows high transparency in the visible range of the optical spectrum. The enhanced photon scattering by doping-induced crystal defects may cause a transmittance drop of up to 3 at. %. Transmittance increased after 4 at. % I_2_ doping, parallel to the resistivity value	Amalina et al. [191], Muiva et al. [216]
Polymer	Wavelength	20% of the composites are the ideal concentration, resulting in films with good visual transparency in polymeric PMMA that are 0.1 mm thick.	Carboni et al. [361]
	Doping concentration	According to the UV–vis transmittance spectra of PVDF pure and PVDF-ZnO nanocomposites, the transmittance of ultraviolet light reduced as the ZnO content in the nanocomposite films increased. The increase in surface roughness is the cause of the observed lower transmittance.	Indolia and Gaur [362]
	Polymer type and molecular structure	Polyethylene has a linear molecular structure comprising repeated ethylene units (-CH_2_-CH_2_-), so light can travel through it without absorption or scattering, resulting in outstanding optical transmittance.	Lahlouh [364]
		PS is composed of an ethylene-linked chain of aromatic benzene rings. The stiff and planar structure of the benzene rings facilitates the efficient transmission of visible light through the polymer matrix. When compared to its lower-molecular-weight competitors, high-molecular-weight PS shows better optical transmittance. PS thin films with longer polymer chains are better suited for high-optical-clarity applications like transparent packaging and optical lenses because they are less flawed and show less light scattering.	Zhou and Burkhart [365], Zhao et al. [366]
		PMMA thin films have a homogeneous molecular structure and little chain branching or crosslinking; as a result, they offer optical clarity and high transmittance throughout the visible spectrum	Klinger et al. [367]
	Film thickness	Reduced transmittance is often the result of thicker polymer sheets’ tendency to absorb light and scatter photons. In the visible spectrum, thin polymer films (less than 100 nm) could transmit higher. Photons may interact with molecular bonds, functional groups, or contaminants in the polymer film during light transmission, resulting in photon absorption and decreased transmittance. The polymer molecules’ absorption of light energy reduces the intensity of transmitted light.	Good et al. [368], Tsilingiris [369]
		As the PET film thickness rises, the polymer material absorbs more incident light, lowering optical clarity and transmittance.	Zhang and Zhang [370]
INO	Temperature	In the visible spectrum, all films exhibited high transparency with different annealing temperatures. Transmittance values drop in the longer wavelength range as the sintering temperature increases. The sample’s fluctuating oxygen content could be the cause. Films sintered at 500 °C displayed a narrower transparent window than those sintered at 300 °C, which may have exceeded the device’s detection limit.	Solieman [372]
		This film has the maximum thickness value. Its high transmittance may be due to the smooth surface feature. The following elements generally cause a decrease in the film’s transmittance: (i) mixed phases, (ii) thickness growth, (iii) defects and oxygen vacancies, (iv) surface roughness, (v) porous nature of the films, (vi) grain boundary scattering, etc. When substrate temperature rises, oxygen desorption may occur, creating oxygen vacancies. Therefore, the film created at T_s_ = 573 K should have the lowest transmittance value and more oxygen vacancies. The transmittance of films created at 300 K, which was the lowest substrate temperature, is high, approximately 96%.	Beena et al. [342]
		As substrate temperature rises, the films’ transmittance also increases.	Gupta et al. [344]
		As the annealing temperature rises from 350 to 500 °C, there is a notable increase in average transmittance. Upon annealing, the crystallinity of thin films increases. Their crystallinity likely influences the transparency of the films’ light scattering. Better transparency results from decreased light scattering as the films’ crystallinity increases.	Senthilkumar et al. [345]
		Crystallization improves the electrical and transmittance qualities of thin films.	Han et al. [373]
	Doping concertation	The transmittance of undoped In_2_O_3_ was the lowest, and it rose as the V doping level increased. The oxygen lost during evaporation is the reason for the poor transmittance of the undoped In_2_O_3_. Following doping, the V-doped In_2_O_3_ films exhibit an increase in transmittance and a decrease in reflectance,	Alqahtani et al. [374]
CdO	Oxygen pressure	The films formed at lower oxygen pressures display poorer transmittance values. Oxygen deficits and the absence of stoichiometry in the film can cause an inadequate oxygen ambiance. A stoichiometric film devoid of oxygen deficits can be achieved at elevated ambient oxygen levels. However, the quality of the film may decrease if the background oxygen pressure rises over this threshold because of the increased collision between laser plume species and background oxygen atoms. While the films deposited at an oxygen pressure of 0.2 mbar exhibit lesser transmittance, the films formed at an oxygen atmosphere of 0.02 mbar show very high transmittance. The film deposited at 0.02 mbar demonstrated significantly higher transmittance than the other films, indicating its superior quality.	Beena et al. [342]
	Temperature	The transmittance of deposited and annealed films rises non-linearly with wavelength The transmittance of deposited films is higher than that of annealed films at the same wavelength.	Ullah et al. [333]
		When a film is annealed at a higher temperature, the absorption edge changes to higher wavelengths.	Santos-Cruz et al. [334]
	Doping concentration	With 10 wt. % Al doping, the transparency falls between 70 and 80 percent in the visible spectrum.	Saha et al. [377]
		The transparency for the pure CdO film is 88%, increasing to 90% for the CdO film doped with 1% Mn. Higher Mn doping concentrations (more than 1 at. %) decrease transparency; the film coated with 4 at. % Mn achieves the lowest value of 78%. As the Mn doping concentration rises, the transmission edge changes to the longer-wavelength side, indicating a decrease in the optical bandgap of the CdO: Mn films. The transparency of CdO: Mn films with 2–4 at. % Mn concentration decreased due to the addition of Mn to the CdO lattice and an increase in the concentration of free charge carriers.	Manjula et al. [378], de Biasi and Grillo [379]
		Adding Al dopant to CdO thin films improves transmittance. The transmittance of the undoped films is 62%, which increased to 77% with 4 wt. % Al doping.	Kumaravel et al. [335]
		The addition of Al to CdO enhances transmission. The transmittance of the doped film containing 1 at. % Al is close to 90%, which is higher than that of the doped film containing 5 at. %. 1 at % doped film has comparatively lower optical scattering than the 5 at % doped films.	Ziabari et al. [339]
		The CdO film’s transmittance increased to 0.5 wt. % of La doping, but it decreased at 0.75 and 1 wt. (%) of doping. Compared to undoped CdO films, La-doped CdO thin films have comparatively high transmittance, suggesting their optoelectronic applications	Velusamy et al. [340]
		The transparency increases significantly with CdO film coated with 2 at. % Mg. At wavelengths longer than 500 nm, the maximum transmittance of the CdO: Mg film coated with 8 at. % Mg is almost equivalent to 90%. The film coated with 2 at. % Mg doping may have a higher thickness, which could account for its low transparency. A higher crystallite size value for this film may cause the increased absorption by free carriers brought about by increased film thickness.	Usharani et al. [338]

**Table 11:** An overview of the impact of major factors on the electrical resistivity and conductivity of conductive thin films.

Thin Films	Factors	Effects on Electrical Resistivity and Conductivity	Authors
AZO	Doping	The film’s resistivity reduced as the Al concentration increased to 0.8.% concentration. The resistivity began to rise with higher doping	[214]
		The resistivity shows minimum values at a certain doping level. Doping Al^3+^ at the Zn^2+^ site creates a free carrier. Increasing the doping level produces more carriers, but, at a certain level, Al atoms cannot replace all Zn atoms, leading to poor donor functionality and causing electron scattering. Aluminum atoms can segregate to grain boundaries as Al_2_O_3_, increasing the grain boundary barrier. This leads to a peak in doping concentration when aluminum is substituted into zinc oxide, but mobility decreases due to scattering and grain boundary barrier effects.	Goyal et al. [406]
		At higher doping concentrations, the increased resistivity can be attributed to the ionization of a small quantity of Al into Al^3+^, which replaces Zn^2+^ in the ZnO film. Up to 3 at. %, carrier concentration increases with an increase in Al concentration. At concentrations greater than 3 at. %, carrier concentration drops due to the creation of neutral defects by growing Al atoms, which do not release free electrons.	Babu et al. [217]
		At 6 at. % Al doping, the resistivity began to rise with the doping concentration. At 2 at. % Al doping, the resistivity achieved a minimum resistivity. Al was ionized into Al^3+^ and substituted for Zn^2+^ when a tiny quantity of Al was added to the ZnO layer. As a result, one zinc atom was exchanged for one aluminum atom to produce one free electron. Conductivity rises due to the enormous number of free electrons that small amounts of aluminum bring into the doped films. Conductivity does not increase with additional increases in aluminum concentration (>2 at. %). Due to its low solubility, excess aluminum cannot be incorporated into the ZnO lattice; instead, it forms neutral aluminum oxide and separates at the grain boundaries. When Al doping is high, there are fewer electrically active Al atoms in the film. Too much dopant causes carrier traps to form in the lattice, lowering the mobility of the Al atoms.	Muiva et al. [216], Lee and Park [445], Nunes et al. [446], Gómez-Pozos et al. [410]
	Surface morphology	Surface leakage reduces the accuracy, impacts, and other characteristics of a high-resistive film’s resistivity values.	Majumder et al. [323]
	Temperature	The resistivity decreased with increasing temperature up to 500 °C; after that, the resistivity began to increase.	Alam et al. [214]
		The films’ resistivity increases, and their crystallinity decreases when annealed beyond 500 °C. This could be connected to the decline in carrier mobility caused by an increase in grain boundary scattering for free electrons	Ning et al. [213]
		The film’s resistivity is reduced when the growth temperature rises from 450 °C to 475 °C. The resistivity then increases to 500 °C from 475 °C The growth temperature impacts the films’ mobility and carrier concentration. When the growing temperature rises, the carrier concentration and the mobility of the films first rise and subsequently fall. A decrease in zinc interstitials or oxygen vacancies at higher temperatures results in a more stable material with high resistivity.	Babu et al. [217]
	Deposition technique	According to the study analysis, the sol–gel films perform significantly better.	Alam et al. [214]
	Film thickness	As film thickness increases, the resistivity of sprayed ZnO thin films decreases. Greater film thickness promotes improved film stacking, which raises grain size and reduces grain defects and grain boundary scattering, both of which are detrimental to carrier mobility.	Achour et al. [413]
ITO	Film thickness	Conductivity increases as grain size and grain boundary decrease, reducing resistivity.	Eshaghi and Graeli [278], Ohhata et al. [405]
		Thinner films might be more vulnerable to substrate effects, which could alter the film’s resistivity.	Bélanger et al. [416]
	Power and deposition time	As a function of power, the rise in carrier concentration causes the resistivity to grow.	Yu et al. [417]
		Similar electrical experiments showed that the resistivity falls when the RF power and the deposition time increase.	Tchenka et al. [325]
	Temperature	The resistance decreases as substrate temperatures increase. An increase in substrate temperature causes an increase in grain size, crystallization, and carrier concentration. As a result of the rise in temperature, resistivity values may also decrease positively due to the shift in the preferred crystallization orientation.	Tuna et al. [324]
	Grain size	The resistivity is inversely proportional to the grain sizes. For DCMS- and RFMS-generated samples, the resistivities decreased by 4.5 and 3 times, respectively, with increased substrate temperature.	Tina et al. [324], Nishimoto et al. [419]
	Bandgap	As substrate temperature rises, the bandgap increases, and resistivity falls.	Geng et al. [420].
	Sputtering voltage	A lower collision energy of negative ions might decrease the resistivity of ITO films. A lower sputtering voltage can effectively decrease the ITO film resistivity.	Ishibashi et al. [421], Sunde et al. [415]
	Carrier concentration	An increase in carrier concentration explains the decreased resistivity at lower sputtering voltage. The carrier concentration rose while the sputtering voltages were decreased, and the mobility remained constant for all substrate temperatures. Negative ion collisions cause damage that mainly affects the concentration of charge carriers and does not affect mobility. An increase in bivalence causes the mechanism of carrier concentration drop by negative ion damage.	Tchenka et al. [325]
CuI	Temperature	When the thin films were annealed in an Ar environment, they showed resistivities between 1 and 5 Ω cm. Compared to films annealed in Ar at the same temperature, the films annealed in air at temperatures below 200 °C show somewhat lower resistivities. At about 130 °C, the lowest resistivity (0.3 Ω cm) was achieved during the annealing process. When the annealing temperature rises, the resistivities of the films annealed in air tended to rise as well, becoming too high to detect at temperatures beyond 250 °C.	Kaushik et al. [56], Inudo et al. [194], Zhu and Zhao [190]
		A resistance of 10 mΩ cm was found for thermally evaporated CuI thin films produced at 120 °C substrate temperature. The increased carrier concentration at lower temperatures is the reason for the as-deposited film’s reduced resistivity	Zi et al. [359]
		The resistivity rises to the order of 1 Ω cm as the substrate temperature rises to 350 °C.	Zhu and Zhao [190]
		The CuI thin-film resistivity increased with increased annealing temperature.	Moditswe et al. [54]
	Cu/I ration	For PLD-prepared CuI thin films, resistivity rose with the I/Cu ratio. Cu^+^ is the dominant carrier, which is why the low resistivity at a lower I/Cu ratio was observed. γ-CuI thin films should achieve high conductivity at a high I/Cu ratio.	Rusop et al. [425], Tanaka et al. [187], Tennakone et al. [426]
	Addition of oxygen	When comparing the PLD-prepared CuI thin films, the addition of oxygen causes a resistivity drop of the order of 10^4^ Ω cm. Adding oxygen to CuI films enhances their conductivity [424]	Herrick and Tevebaugh [427]
	Doping concentration	The resistivity rose for films manufactured with a 3 at. % doping concentration. Iodine doping can raise or lower the resistivity, which relates to where the iodine-induced surface traps are located in the CuI band position. Since the interstitial I_2_ atoms in the lattice operate as a charge trap for electrons, the resistivity likely increases as the concentration of I_2_ ions increases.	Amalina et al. [191]
		The thin-film cracking brings on the maximum resistance; The formation of fracture coatings reduces grain boundaries, which enhances carrier mobility and is responsible for the reduced resistivity at 4 and 5 at. % doping concentrations due to carrier scattering.	Perera et al. [428], Amalina et al. [191]
		High optical conductivity and lower electrical resistance were reported for CuO thin films doped with Zn.	Nesa et al. [429]
		Ti dopant improves the conductivity of the films while maintaining the samples’ optical qualities and crystallinity.	Saied et al. [430]
		As the Ni concentration of the CuO thin film increases, both the optical bandgap and electrical resistance increase.	Uddin et al. [431]
INO	Oxygen pressure	The INO films created in an oxygen-free atmosphere show a low value of electrical resistivity.	Beena et al. [342], Yamada et al. obtained [435]
		A lower DC electrical resistivity value (1.7 × 10^−6^) Ω m for INO films created at an oxygen pressure of 10^−3^ mbar has been found.	Gupta et al. [436]
		The films created at 0.02 mbar have a somewhat greater oxygen pressure. The films produced in this manner can be nearly stoichiometric, as indicated by their high optical transparency and improved stoichiometric character, thus leading to a comparatively higher DC electrical resistivity analysis value. These films have incredibly few oxygen shortages.	Solieman [372]
	Temperature	With the increasing temperature, the resistivity decreases.	Beena et al. [342]
Ti-doped Al_2_O_3_	Doping	Ti-doped Al_2_O_3_ thin films show improved electrical conductivity due to the creation of oxygen vacancies and Ti^3+^ states, which act as electron donors. Ti^3+^ doping induces impurity band states, forms Ti^3+^ clusters in Al_2_O_3_ GBs, and can control their electrical band structures. This segregation of Ti^3+^ clusters in GBs may also enhance the conductivity in Ti-doped Al_2_O_3_. Ti^3+^ ions at the GBs introduce an extra impurity band in the bandgap, which may be responsible for the Ti-doped polycrystalline Al_2_O_3_’s improved conductivity through the GBs.	Yang et al. [439], Unno et al. [440]
CdO	Doping	The conductivity increases as aluminum concentration increases, but doping concentration ranges from 1 to 5%. It was found that conductivity decreases as the doping percentage of Al increases by more than 5%. Aluminum doping increases the concentration of free charge carriers in CdO due to the extra valence electron it contains. It can replace the cadmium atom or occupy interstitial sites, acting as the donor material. However, excessive doping may damage the crystalline structure. At greater Al concentrations, the conductivity in CdO: Al thin films decreases due to an increase in free carrier absorption. Once more, a minor amount of Al_2_O_3_ formed at a greater doping level may also decrease conductivity.	Saha et al. [377], Maity and Chattopadhyay [444]
	Deposition technique	RF sputtering generally has higher film quality and the ability to fabricate device-quality films. Using the RF sputtering approach, one may manage more aspects of the deposition process, such as substrate temperature, RF power, gas partial pressures, etc. Conversely, the sol–gel method is less complicated and more affordable.	Saha et al. [377], Maity and Chattopadhyay [444]

**Table 12:** Challenges and their possible solutions associated with thin films are addressed by different authors.

Authors	Challenges Addressed		Possible Solutions
	Issues	Abbreviation	
Naghdi et al. [30]	Sintering process and substrate compatibility	The selection of substrates is limited by the sintering process’s temperature requirements, which are necessary to enhance the conductivity of thin coatings. Although raising the sintering temperature can improve conductivity, it also increases the risk of pore formation and reduced functionality. To increase the variety of substrates, the sintering temperature must be lowered.	Reducing the size of nanomaterials can increase film conductivity, increase substrate possibilities, and decrease the sintering temperature.
	Problem associated with film thickness	A thicker film has better electrical conductivity but less optical transparency. The ideal thickness must be determined to balance these opposing qualities.	Determining the ideal thin-film thickness that balances optical transparency and electrical conductivity should be maintained.
	Aspect ratio	Increasing the aspect ratio lowers the percolation threshold, which is essential for cutting production costs without sacrificing mechanical qualities.	Conductive metal nanoparticles can be incorporated into polymers or mixed with other nanomaterials to generate hybrid materials with improved mechanical properties, conductivity, and customized functionality.
	Adhesion problem	Poor adhesion between the substrate and the thin film during deposition can cause problems when the film is stretched or bent, jeopardizing its integrity and performance.	By employing polydopamine, the substrate’s wettability can be changed. The hydrophilic condition of the substrate surface facilitates the deposition of Ag NWs, which improves the adhesion between the thin film and the elastomeric substrate. This method may create a transparent Ag NW thin film with improved adhesion, excellent optical transparency, minimal sheet resistance, and stability, even after the sample has been stretched.
Litzelman et al. [545]	Variations in the properties	The characteristics of nanostructured electrode and electrolyte components produced using thin-film fabrication techniques can differ greatly from those of bulk materials.	Minimizing differences in characteristics from bulk materials by improving thin-film fabrication methods.
	Variations in ionic conductivity	The performance of SOFCs can be affected by changes in ionic conductivity caused by internal interfaces within the nanostructured components.	Designing interfaces to improve ionic conductivity or reduce adverse effects, maybe by changing the materials’ surface or chemistry.
	Long-term stability	Increased cation diffusion along grain boundaries may affect the devices’ long-term stability. Enhancing membranes’ mechanical stability and thermomechanical dependability, especially when exposed to greater temperatures and for extended periods of time, is crucial.	Finding the sources of lingering tensions and the progression of stresses in the films is crucial to achieving this. Furthermore, using stress-tolerant designs is a possible remedy to alleviate these problems.
Zhang et al. [546]	Achieving high performance and stability at a low cost	The technical challenge is to minimize costs while maintaining high performance and stability. This requires creating large cells with low grain boundary resistance and thin, gas-tight electrolytes.	Using theoretical insights from density functional theory investigations, and machine learning can be used in material design to expedite the process and find useful characteristics for innovative materials.
Kalinina et al. [547]	Creating conductivity on non-conductive substrates	Conductivity must be established on the surface of non-conductive substrates in order to apply electrophoretic deposition (EPD).	Developing methods for the successful deposition of the Ce_0.8_Sm_0.2_O_1.9_ (SDC) electrolyte film, such as the formation of conductive sublayers such as PPy and Pt on the front surface of the non-conductive substrate.
	Mechanical stresses in the formation of bilayer films	The EPD of CuO-modified BaCe_0.5_Zr_0.3_Y_0.1_Yb_0.1_O_3−δ_ (BCZYYbO-CuO/SDC) and SDC films on reduced Ni-SDC substrates results in significant mechanical strains that cause cracks in the bilayer film and disintegration of the single SDC film during the oxidative co-sintering process that follows.	Changing the anode substrate’s composition or altering the deposition process’s characteristics
Patil et al. [548]	Achieving high energy density and power density	A difficulty in developing lithium-based thin-film rechargeable batteries is achieving higher energy and power densities without sacrificing appropriate power densities.	By increasing the energy density of thin-film batteries, concentrating on technology development and improvements in the electrode process, within the next five to six years, it is possible to obtain energy densities exceeding 500 Wh/L and 200 Wh/kg by investigating novel active materials and electrode technologies.
Liu and Wöll [549]	Thin-film quality	For MOF thin films to find widespread use, their surfaces must be smooth, crystalline, aligned, compact, uniform, and free of pinholes, yet this is still a difficult task.	The film can be improved and oriented by employing techniques such as ALD and Liquid Phase Epitaxy (LPE).
	Defect control	Flaws must be controlled for MOF thin films to function as well as possible, yet characterizing and managing defects is difficult.	Advanced characterization methods, such as electron microscopy, spectroscopy, and synchrotron X-ray diffraction, can be utilized to study and manage flaws in MOF thin films. Techniques like “defective linkers” or controlled heating introduce defects in a controlled manner.
	Enhancing electrical conductivity	The intrinsic features of most <span id=“zotero-drag”/> MOFs make them poor electrical conductors, which restricts their use in energy storage systems.	Techniques include loading organic guest molecules into MOF voids, creating conductive polymers inside MOF frameworks, or creating MOFs with intrinsic conductive qualities by utilizing highly conjugated linkers, which can all be used to improve electrical conductivity.
Eslamian [550]	Development of Continuous and Integrated Thin Films	Spray coating presents a difficulty in producing thin layers that are consistent, high-quality, and integrated. The efficiency of current approaches is frequently lower than that of spin-coating, indicating a need for improved spray coating techniques.	Applying spray-coating-specific optimal processing conditions can improve the homogeneity and quality of thin films. Fine-tuning variables can enhance film morphology and device performance, including substrate temperature, nozzle distance, and spray rate.
	Effective Charge Separation, Transfer, and Collection	One of the largest challenges in spray-on layers is to ensure effective charge separation, transfer, and collection.	Using numerous spray passes increases the device’s overall efficiency by improving the coating’s thickness and coverage. Iterative deposition methods enable improved control over the film’s shape and characteristics.

## Data Availability

No new data is created for this review paper. However, the tables and figures created for this review paper will be available.
